# Recent Advances in Sensor-Based Upper-Limb and Hand Exoskeletons for Post-Stroke Rehabilitation: A Technical and Biomedical Review

**DOI:** 10.3390/s26175373

**Published:** 2026-08-25

**Authors:** Alberto Borboni, Matteo Verzeletti, Alireza Rastegarpanah, Jorge Hugo Villafañe

**Affiliations:** 1 Department of Mechanical and Industrial Engineering, Università degli Studi di Brescia, 25123 Brescia, Italy; 2 School of Engineering and Physical Sciences, Aston University, Birmingham B4 7ET, UK; a.rastegarpanah@aston.ac.uk; 3 Casa de Villafañe, 10045 Piossasco, Italy; mail@villafane.it

**Keywords:** sensor-based upper-limb and hand exoskeletons, post-stroke rehabilitation, wearable robotic orthoses, sensor fusion, sensor-driven control, home-based telerehabilitation, artificial intelligence in rehabilitation robotics

## Abstract

**Background:** Recent advancements in enabling technologies, including artificial intelligence and telemedicine, alongside robust clinical study outcomes, have led to significant progress in upper limb and hand exoskeletons utilised for post-stroke rehabilitation. **Objectives:** This review aims to synthesize the recent scientific literature (2010–2025) on post-stroke upper-limb and hand exoskeletons, with particular attention to the sensing architectures—sensing modalities, signal processing, sensor fusion, and sensor-driven control—that integrate technical and biomedical domains to examine device architecture, clinical context, and outcome selection. **Methods:** A search of PubMed and Scopus was conducted on 10 November 2025, cross-checked against IEEE Xplore, Web of Science, Embase, and ACM Digital Library. We included studies evaluating wearable exoskeletons or robotic orthoses for the upper limb/hand in post-stroke rehabilitation. Two independent reviewers screened records and extracted data, with disagreements resolved by consensus. Data were synthesised using a predefined label-based taxonomy. The review protocol was not registered. **Results:** From 1889 identified records, 219 studies met the inclusion criteria. The synthesis reveals a transition from rigid, laboratory-centered systems to lighter, soft, and home-oriented solutions. Available evidence suggests potential impairment-level benefits, particularly for proximal motor control, but certainty remains limited due to heterogeneity, small samples, blinding limitations, inconsistent dosing, and limited long-term follow-up; gains in hand/finger dexterity appear even more variable. **Discussion:** While exoskeleton-assisted therapy appears associated with impairment-level gains, transfer to activities of daily living (ADLs) and real-world function remains inconsistently documented and insufficiently powered to support firm conclusions.

## 1. Introduction

The burden of stroke continues to be a major public health concern due to its high prevalence of death and disability worldwide. The Global Burden of Disease 2019 report indicates that there are more than 12 million first-time strokes every year and that an estimated 100 million people currently alive have had a stroke [1,2]. Additionally, the World Health Organization reports that stroke is the second leading cause of death and third leading cause of death and disability combined, resulting in over 143 million Disability-Adjusted Life Years (DALYs) lost annually [1,2,3]. Stroke accounts for approximately 11% of all deaths globally, with a lifetime risk that has increased by nearly 50% over the last two decades and currently impacts one in four adults aged 25 and older [1,4,5]. The burden varies geographically, with approximately 70% of all strokes and almost 90% of all stroke-related deaths occurring in low- and middle-income countries, where incidence has more than doubled over the last decade [6,7]. Beyond mortality, the total cost of stroke exceeds $700 billion annually, representing greater than 1% of world GDP when combining direct medical expenses and lost productivity [1,8].

Stroke is increasingly recognised as a chronic condition resulting in lasting impairments for survivors, their families, and health systems. Over 100 million people alive today have experienced a stroke, with a large proportion acquiring disability before the age of 70 [1,2,5]. A significant number of stroke survivors live for years or even decades with lingering impairments that limit their ability to perform independently, participate in activities, and return to work [5,9]. Interventions aimed at reducing post-stroke disability rather than only mortality are therefore essential to mitigating the individual and societal burden of stroke.

One of the most common and disabling sequelae of stroke is motor impairment of the upper limb, which affects a substantial proportion of survivors and often persists beyond the acute phase. Between 75% and 85% of survivors experience upper-limb motor impairment, and less than half regain normal upper-limb function [10,11,12]. Longitudinal studies have demonstrated that 50–60% of stroke survivors retain impaired arm and hand function at six months post-stroke, and a significant proportion remain without functional use of the paralysed upper limb in the chronic phase [10,12,13,14]. Hand and finger dexterity impairments are particularly problematic: two-thirds of stroke survivors are unable to fully integrate the affected hand into activities of daily living (ADLs) at six months, and only about one-fifth regain sufficient upper-limb function for regular bimanual activities [13,15]. Impaired upper-limb and hand function are strongly correlated with diminished capabilities in both basic and instrumental ADLs, reduced participation, and overall diminished quality of life [15,16,17]. Patients consistently rank recovery of arm and hand function among the top rehabilitation priorities following stroke [18].

Traditional post-stroke rehabilitation for the upper limb consists primarily of physiotherapy and occupational therapy, encompassing impairment-focused treatments, task- and goal-oriented training, constraint-induced movement therapy (CIMT), bilateral arm training, sensory and motor relearning programmes, mirror therapy, and mental imagery [19,20,21,22,23,24,25,26,27]. Modern neurorehabilitation principles emphasise early intervention and high-intensity, repetitive, task-specific practice to capitalise on activity-dependent neuroplasticity [20,21]. While these approaches can improve motor function and, to a lesser degree, functional independence, they are frequently limited by available resources, therapist availability, and the challenge of delivering sufficient dose over extended periods in community settings following discharge from inpatient facilities [19,28]. These limitations have prompted the development of numerous technologies to provide complementary forms of technology-enabled upper-limb training [29].

Over the past two decades, a wide range of technology-enabled rehabilitation modalities have been developed. These include robot-assisted therapy, virtual and augmented reality environments, serious games, and sensor-based systems (inertial, force, and pressure) [30,31,32,33,34,35,36]. Systematic reviews and meta-analyses of robotic upper-limb therapy (encompassing both end-effector and exoskeleton robots) have found that this approach produces at least equal and in many cases greater improvement in motor impairment compared to equivalent doses of conventional therapy, particularly in the chronic phase [30,37,38,39,40,41]. However, findings regarding the impact on ADLs and participation remain inconsistent and are complicated by methodological heterogeneity across trials [30,37,38]. Virtual reality and game-based therapies have been employed to increase patient engagement and dose of practice, while wearable sensors and tele-rehabilitation platforms allow for remote monitoring and coaching, facilitating therapy delivery outside the clinical setting [31,32,33,42]. A large number of exoskeleton-based technologies have emerged for upper-limb and hand rehabilitation, attracting significant attention within rehabilitation robotics. Exoskeletons are wearable robotic devices consisting of mechanical structures configured according to the anatomy of the wearer’s upper limb and hand, fitted with actuators and sensors to assist, impede, or enhance voluntary motion while controlling joint kinematics [33,43,44,45,46]. There exists a wide variety of upper-limb and hand exoskeletons ranging from rigid, multi-degree-of-freedom devices to cable-driven systems, soft robotic systems, and increasingly lightweight, ergonomic, and modular devices. It is worth noting that terminology in this field is not uniformly standardised: terms such as “exoskeleton”, “exosuit”, “robotic orthosis”, and “soft glove” are used with varying meanings across engineering and clinical disciplines, and even across research groups. For instance, the ISO definition of a robot requires at least two independently controlled actuators, which distinguishes a robotic orthosis from a simpler powered splint; a soft glove may or may not meet this threshold depending on its actuation architecture. The present review adopts operational definitions for each device class that are detailed in Section 3 alongside the label taxonomy, and acknowledges that some boundary cases exist. Sensing is a central enabler of exoskeleton function and a primary analytical focus of this review. Exoskeleton systems integrate multiple sensing modalities to close the loop between device mechanics, human biomechanics, and voluntary user intent. Kinematic sensing—most commonly through inertial measurement units (IMUs) and encoder-based joint angle measurement—provides the baseline for feedback control and movement monitoring. Force and torque sensing at the human–robot interface enables compliant control strategies (impedance and admittance control) and safe physical human–robot interaction. Electromyographic (EMG) sensors decode voluntary muscular intent to drive effort-coupled and assist-as-needed assistance. Electroencephalographic (EEG) sensors and brain–computer interfaces (BCIs) extend intent decoding to patients with severely limited voluntary motor output. Vision-based systems (RGB-D cameras, optical motion capture) serve both technical validation and environmental perception roles. The integration of multiple modalities into sensor fusion architectures—combining, for example, IMU kinematics with EMG intent signals and force/torque safety monitoring—represents the current direction of the field and directly determines the quality of intention detection, assist-as-needed control, safety monitoring, clinical outcome assessment, and suitability for home deployment. The purpose of exoskeleton devices is to provide high-intensity, repetitive, and task-specific training to address reaching, grasping, manipulating objects, and coordinating multi-joint movements of the upper limb. Advanced control strategies such as assist-as-needed, impedance control, and cooperative modes enable the mechanical assistance to be adjusted based on the user’s residual capacity, thereby encouraging active participation in therapy and promoting neuroplasticity [47,48,49,50,51,52].

Over time, there has been a growing trend of adopting exoskeleton technology in both research and clinical practice for post-stroke upper-limb rehabilitation. Numerous devices target different segments of the upper limb—shoulder and elbow, wrist, hand, and fingers—and several cover the entire upper limb to support coordinated reaching and grasping [33,43,53,54,55,56,57]. Clinical trials and systematic reviews have reported improvements in upper-limb motor function following exoskeleton-assisted therapy, particularly as measured by the Fugl-Meyer Assessment for the upper extremity (FMA-UE) and the Action Research Arm Test (ARAT), with the most consistent gains observed at proximal joints [40,58,59]. A smaller and more variable degree of improvement in hand and finger dexterity has been reported, underscoring the need for continued innovation in hand-specific exoskeleton design and control. Studies using neurophysiological methods have demonstrated changes suggesting increased corticospinal excitability and activity-dependent plasticity following exoskeleton-based interventions [41,60,61,62]. Despite this progress, significant barriers to widespread clinical application remain, including device cost and maintenance, physical size and weight, user comfort, and the limited translation of impairment-level gains to sustained improvements in ADLs and real-world participation [46,62,63].

As the scientific literature continues to expand rapidly, aggregated database searches have identified approximately one thousand relevant papers published since 2007, with the vast majority of technological innovations and clinical trials concentrated in the past fifteen years [33,43,44,53,64,65,66,67,68]. A significant increase in exoskeleton-related publications began around 2012, corresponding to the availability of more compact actuators, improved human–robot interfaces, and integrated virtual or augmented reality environments. Prior to 2010, work focused primarily on laboratory prototypes with limited degrees of freedom, whereas more recent devices are increasingly designed for clinical deployment, home use, and rigorous evaluation in controlled trials [33,43,44,53,64,65,66,67,68]. Each successive generation has introduced qualitatively new capabilities—soft wearable architectures, AI-based adaptive controllers, and hybrid BCI-EMG control—not represented by older systems [33,69]. At the same time, the cumulative evidence base across multiple systematic reviews and meta-analyses, many with overlapping but non-identical inclusion criteria, creates a complex landscape with varying study designs, patient characteristics, intervention dosages, and outcome measures [70,71,72].

To address these rapid advancements and the volume of available studies, it is methodologically appropriate to limit the synthesis to a focused and recent time frame. Prior comprehensive reviews of rehabilitation robotics have similarly limited their searches to articles published from 2010 onward, citing that early work involved mostly small trials and devices that bear no resemblance to current systems [30,44,45,73]. Accordingly, the present review covers studies published from 2010 to November 2025. The present work is designed as a narrative review, informed by aspects of the PRISMA workflow (structured search strings, dual independent screening, standardised extraction, PRISMA flow diagram) without constituting a formal systematic review or umbrella review. It integrates evidence from primary studies (clinical trials, observational studies, technical engineering studies, case studies, and protocol papers) as well as previous reviews and meta-analyses within a unified label-based synthesis framework. As such, clinical effectiveness estimates reported here are drawn from the conclusions of included primary studies and existing reviews; this narrative review does not independently compute pooled effect sizes or conduct meta-analytic statistical synthesis; clinical claims in Section 4.2 are restricted to studies classified as RCTs or clinical trials. The included studies span three functionally distinct categories: (i) clinical studies involving stroke patients in rehabilitation settings, (ii) technical validation studies conducted with healthy subjects or in laboratory conditions, and (iii) engineering prototype studies developed for rehabilitation applications without human participant testing. This distinction is maintained throughout the synthesis to prevent conflation of clinical efficacy evidence with technological development trends. Specifically, the present review intends to: (i) summarise the types and technical features of upper-limb and hand exoskeletons utilised for post-stroke rehabilitation since 2010, with particular attention to sensing architectures and their role in control and clinical assessment; (ii) synthesise the reported clinical evidence regarding the efficacy of these technologies, emphasising manipulative function and hand dexterity; (iii) describe the training paradigms and modes of human–robot interaction; and (iv) identify methodological limitations, research gaps, and future directions. Compared with recent reviews, the added value of the present work lies in four specific elements: exclusive focus on post-stroke upper-limb and hand exoskeletons; integration of engineering descriptors (anatomical target, structure, actuation, sensing, control, and AI/telerehabilitation features) with biomedical descriptors (stroke stage, setting, study design, and outcome domain) within a single label-based synthesis; explicit proximal-versus-distal distinction; and examination of translational readiness across laboratory, inpatient, outpatient, and home settings. The novelty of this review lies in connecting device architecture, sensing systems, clinical context, and outcome selection within a unified cross-disciplinary framework designed for both technical and biomedical audiences.

## 2. Materials and Methods

**Figure 1:**
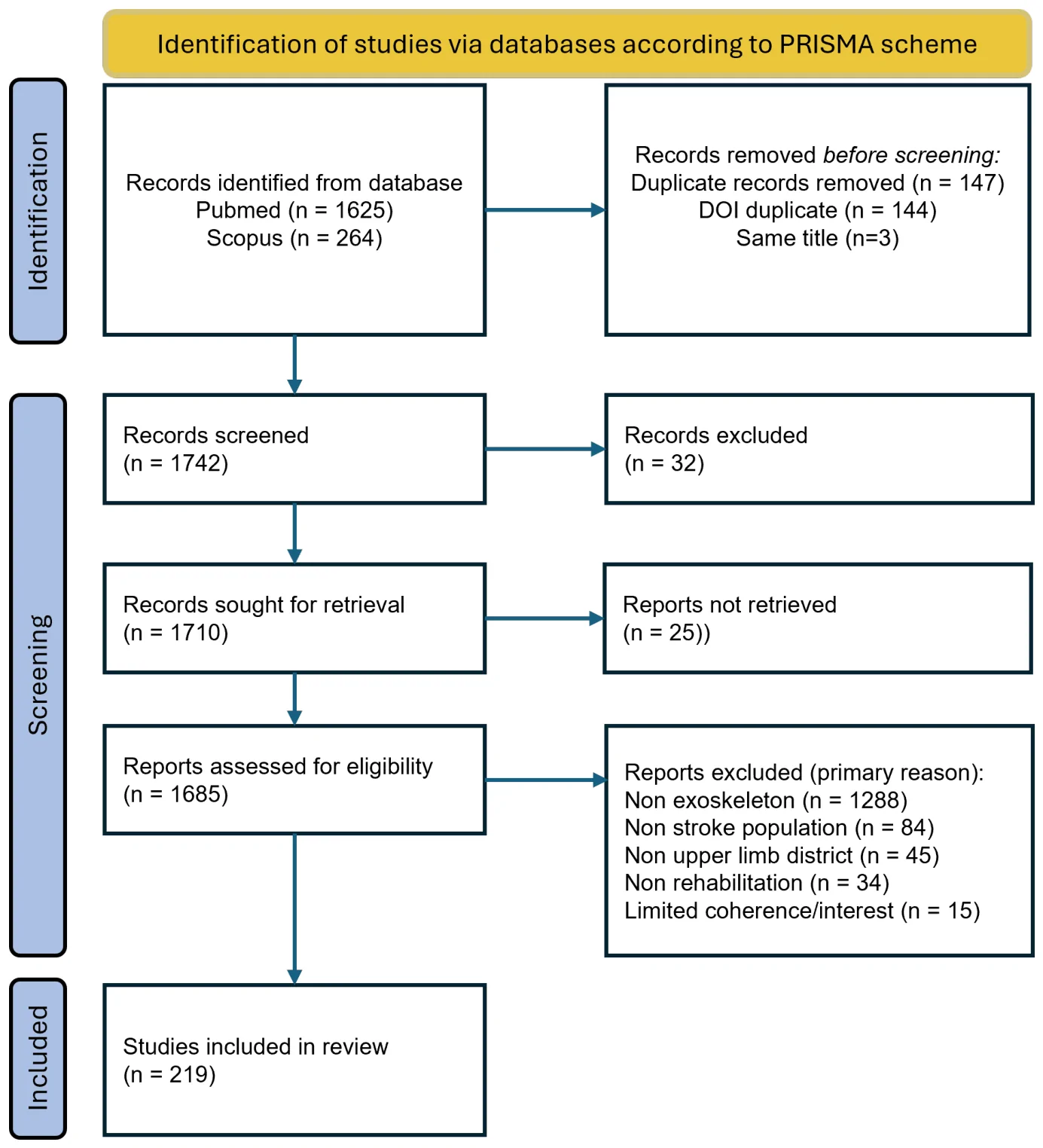
PRISMA flowchart of the study selection process.

A systematic literature search was undertaken using the PubMed (via National Library of Medicine) and Scopus (via Elsevier) databases. The search was conducted on 10 November 2025, covering literature published from 1 January 2010 to 10 November 2025. Only peer-reviewed articles published in English were considered eligible for inclusion. Conference proceedings, preprints, and grey literature were excluded. Scopus provides comprehensive indexing coverage of major engineering and biomedical publication venues, including IEEE Xplore, Web of Science, Embase, and ACM Digital Library. To verify the adequacy of this indexing coverage, supplementary exploratory searches using the same core search terms were additionally run directly in IEEE Xplore, Web of Science, Embase, and ACM Digital Library; these did not identify eligible records beyond those already captured through PubMed and Scopus, and were therefore not incorporated as separate sources in the PRISMA flow (Figure 1). Grey literature, preprints, and clinical trial registries were not searched: consistent with the narrative (non-systematic) design of this review, the synthesis was deliberately grounded in peer-reviewed literature to preserve methodological and citability consistency. Each database used the query string from Table S1 (Supplementary Material) with the appropriate database-specific syntax (i.e., controlled vocabulary and free-text terms in PubMed; Title/Abstract/Keyword fields in Scopus). All retrieved citations were exported and subjected to screening and eligibility assessment. The process of selecting studies followed a structured PRISMA-based workflow, shown in Figure 1.

### 2.1. Protocol and Registration

The present work is designed as a narrative review of heterogeneous evidence—including clinical trials, randomised controlled trials (RCTs), observational studies, technical engineering studies, case studies, and protocol papers, as well as previous reviews and meta-analyses—within a unified label-based synthesis framework. The review was informed by elements of the PRISMA workflow but was not conducted as a formal systematic review or umbrella review: no protocol was registered and no standardised risk-of-bias instrument was applied as a mandatory step (Section 2.5). As a literature synthesis study not involving human participants or animal experimentation, formal prospective registration in a clinical trial registry (e.g., PROSPERO) was not applicable. No formal protocol document was prepared or published prior to the review, and no material deviations from the initial research plan occurred during the conduct of the review. This absence of prior registration is acknowledged as a transparency limitation.

### 2.2. Eligibility Criteria and Study Classification

Included studies had to satisfy all four of the following core eligibility criteria: (1) Exoskeleton/Robotic Orthosis: the study described or evaluated a wearable robotic assistive device (e.g., exoskeleton, exosuit, wearable robot, robotic glove) or a powered/robotic orthosis. (2) Upper Limb/Hand: explicit mention of the upper extremity (arm, forearm, hand, wrist, or finger). (3) Stroke: explicit mention of stroke or post-stroke conditions (stroke, post-stroke, CVA, hemiplegia, hemiparesis, and related terms). (4) Rehabilitation Context: explicit rehabilitation framing (rehabilitation, therapy, training, neurorehabilitation, physiotherapy, occupational therapy, telerehabilitation, or home-based). References to sensors, actuators, control systems, or AI were annotated when present but were not required for inclusion. Common exclusions included lower-limb/gait exoskeletons, studies focused on non-stroke populations without clear post-stroke application, and non-wearable systems (e.g., end-effector devices) not identifiable as exoskeletons or robotic orthoses. For the purposes of synthesis, all included studies were classified into three functionally distinct categories: (i) clinical studies, involving stroke patients in rehabilitation settings; (ii) technical and laboratory validation studies, conducted with healthy subjects or in controlled laboratory conditions without stroke patients; and (iii) engineering prototype studies, describing device development and bench-level testing without human participant testing of any kind. A single study could belong to more than one category where its scope spanned multiple contexts. This classification is maintained throughout the synthesis to prevent conflation of clinical efficacy evidence with technological development trends.

### 2.3. Screening and Study Selection

Two reviewers independently screened the titles, abstracts, and keywords of all retrieved records. A record was retained at the screening stage if its available metadata indicated a plausible match with all four core eligibility domains, even when device class or population could not be confirmed with certainty from the abstract alone. This intentionally high-sensitivity strategy concentrated exclusions at the full-text stage, where non-exoskeleton systems, non-stroke populations, non-upper-limb applications, and non-rehabilitation reports could be identified with greater reliability. Any disagreements during screening or full-text assessment were resolved through discussion and consensus; a third senior reviewer was consulted when necessary. Inter-rater agreement was not formally quantified (e.g., via Cohen’s kappa); this is acknowledged as a methodological limitation in Section 4.2.5. No automation tools were used in the selection process. A total of 1889 records were identified (PubMed: *n* = 1625; Scopus: *n* = 264). Prior to screening, 147 duplicate records were removed (144 DOI duplicates; 3 identical-title duplicates), yielding 1742 unique records for screening. At the title/abstract/keyword screening stage, 32 records were excluded—primarily on grounds of manifestly irrelevant subject matter (e.g., lower-limb rehabilitation devices, non-rehabilitation engineering applications) identifiable from the title alone—and 1711 records were advanced to full-text retrieval. Of these, 25 reports could not be obtained, allowing 1686 full-text reports to undergo eligibility assessment. Of those, 1466 reports were excluded at full-text assessment for the following primary reasons: non-exoskeleton interventions (*n* = 1288), non-stroke populations (*n* = 84), non-upper-limb applications (*n* = 45), non-rehabilitation settings (*n* = 34), and limited coherence with the review question (*n* = 15). In the end, 219 studies met the eligibility requirements and were included in the review.

### 2.4. Data Extraction and Labelling

**Figure 2:**
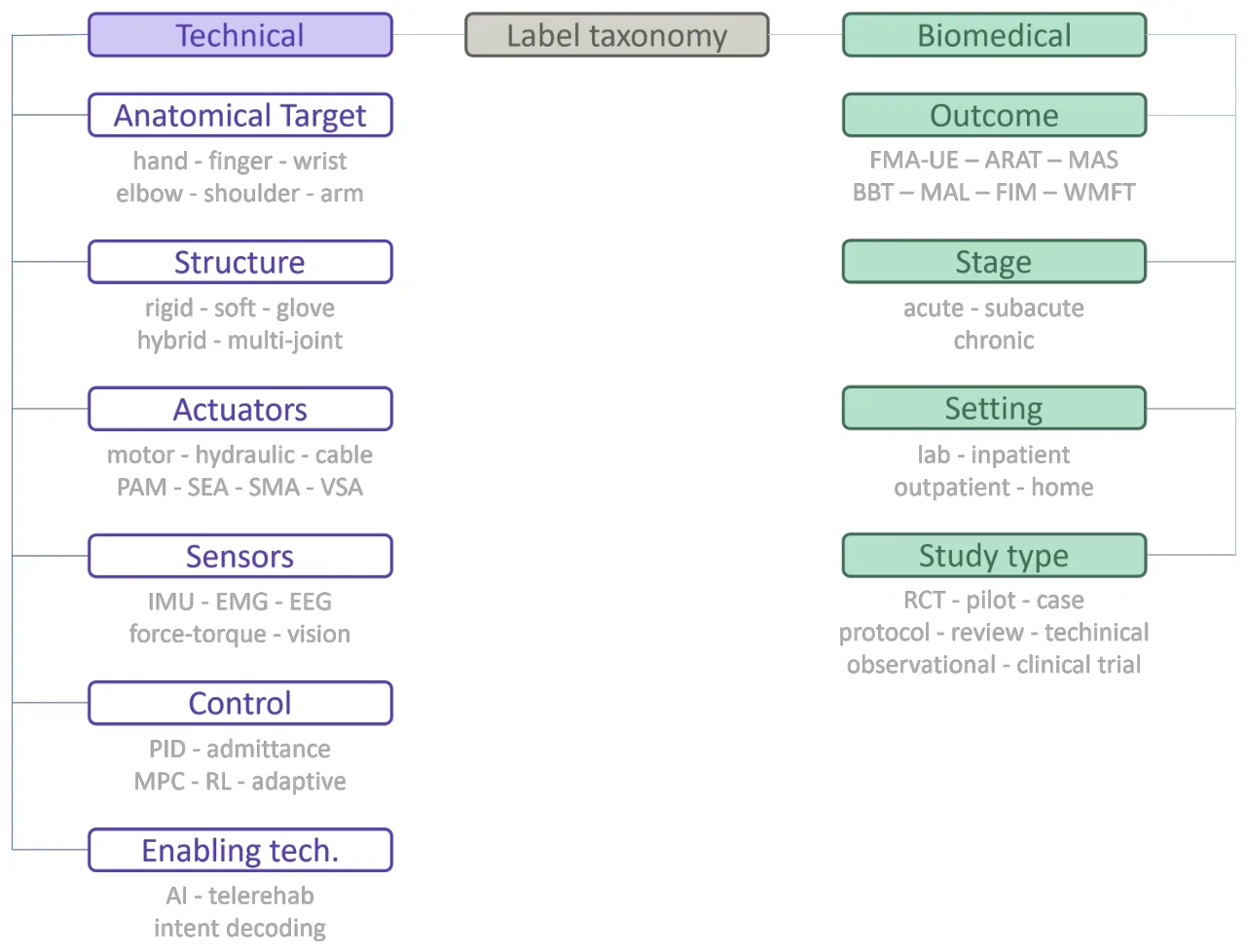
Label taxonomy.

Key information was abstracted into a structured dataset covering bibliographic metadata, study design and location, participant demographics, device and intervention characteristics, and outcome domains where applicable. Data extraction was performed independently by two reviewers using a standardised, pre-piloted data collection form. Discrepancies were resolved through discussion and consensus, with adjudication by a third reviewer when necessary. As with screening, inter-rater agreement for data extraction and label assignment was not formally quantified. Study authors were not contacted to request missing or unconfirmed data; the review relied solely on published reports. Labels were assigned manually according to the predefined schema described in Figure 2. Label assignment followed a strict explicit-evidence rule: a label was applied only when the corresponding feature was explicitly reported in the title, abstract, or—when classification remained uncertain—in the full text; a feature was recorded as absent when information was missing or unclear, rather than inferred from context. This conservative rule likely introduces a systematic under-reporting bias directionally skewed against infrequently documented technical details (e.g., sensor calibration procedure, validation method, and sampling rate) relative to routinely reported features (e.g., device actuation type); prevalence estimates for the former should therefore be read as lower bounds rather than true population frequencies. This limitation is revisited in Section 4.2.5. Multiple labels were permitted for multi-component devices or studies reporting more than one setting, stage, or outcome. For outcome domains, all compatible measures and time points reported in each study were recorded. The label categories and their operational definitions are provided alongside the taxonomy figure (Figure 2).

### 2.5. Methodological Quality Considerations

As this work is a review rather than a formal systematic review, no standardised risk of bias instrument (e.g., RoB 2 for RCTs, MINORS for non-randomised studies) was applied as a mandatory assessment step. The methodological quality of the included evidence is instead evaluated narratively throughout the Discussion, with explicit acknowledgement of the predominance of small, non-randomised, and non-blinded study designs in the clinical subset of the literature. Common methodological shortcomings—including absence of prospective sample size calculation, lack of an unbiased endpoint assessment, and inadequate follow-up durations—are noted where they bear on the interpretation of reported clinical findings. These considerations are consolidated in the Limitations section (Section 4.2.5) and are used to contextualise, rather than exclude, the evidence synthesised in this review.

### 2.6. Synthesis Approach

Given the methodological heterogeneity of the included studies—spanning engineering prototypes, pilot feasibility studies, observational cohorts, and RCTs across diverse device architectures—a formal quantitative meta-analysis was not appropriate. Instead, a descriptive, label-based narrative synthesis was conducted. To display results and identify trends over time, simple frequencies of the assigned technical and biomedical labels per publication year were calculated and plotted as bar charts and distribution graphs. All counts in the results figures represent the number of included studies carrying the corresponding label; since a single study may carry multiple labels, the same study may appear across multiple category counts. Percentages are calculated over the total of 219 included studies.

The label-based taxonomy adopted in this review classifies included devices into four structural categories, whose operational definitions are provided here to ensure consistent interpretation across disciplines. These definitions reflect the conventions most widely used in the reviewed literature, while acknowledging that terminology is not uniformly standardised across engineering and clinical communities and that boundary cases exist. An exoskeleton is a wearable robotic device whose rigid or semi-rigid mechanical structure is configured in parallel with the user’s limb segments, transmitting assistive or resistive forces through anatomically aligned joints. By ISO 8373 [74] convention, a robot requires at least two independently controlled actuators; an exoskeleton satisfying this criterion is therefore a robotic device in the strict sense, capable of independently actuating multiple degrees of freedom. The term exosuit (or soft exoskeleton) refers to a wearable assistive system built on a compliant textile or fabric substrate—rather than rigid links—that transmits forces through tendons, cables, or pneumatic elements routed along the limb. Exosuits prioritise low distal mass, comfort, and alignment tolerance at the cost of reduced kinematic precision compared to rigid structures. A robotic orthosis is a powered orthotic device that augments or substitutes joint function; when it meets the ISO two-actuator threshold it qualifies as a robotic device, but the term is also used in the literature for single-actuator powered splints that do not technically constitute robots under that definition. A soft glove (or robotic glove) is a hand-specific wearable device—typically fabric or silicone-based—that actuates finger flexion and/or extension through tendon routing, pneumatic chambers, or shape-memory elements. Soft gloves may or may not meet the ISO multi-actuator threshold depending on whether fingers are driven by independent actuators or coupled through shared tendons; studies in this review include both configurations, and the label “glove” is applied to all hand-worn compliant devices regardless of actuator count. A hybrid device combines a robotic exoskeleton or exosuit with functional electrical stimulation (FES), exploiting the complementary mechanisms of electromechanical and neuromuscular actuation within a single intervention. In cases where a study described a device that did not clearly fit a single category—for example, a rigid-frame device with a soft glove hand module—the most prominent structural feature of the device as described by the authors was used to assign the primary label, and secondary labels were applied where additional features were explicitly reported. The taxonomy figure (Figure 2) illustrates the full two-branch label schema covering both engineering and biomedical dimensions.

## 3. Results

**Figure 3:**
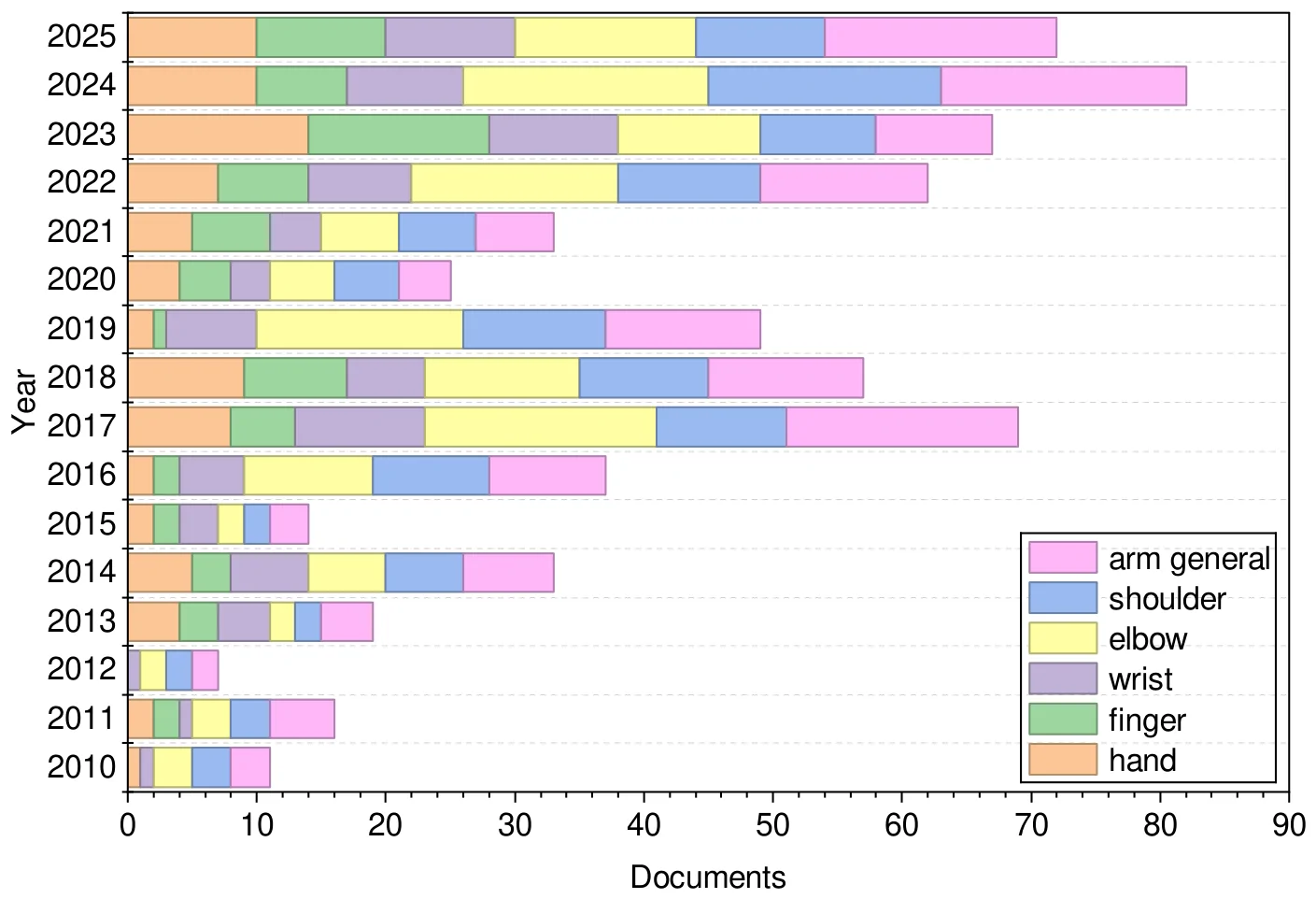
Anatomical target of the exoskeleton.

**Figure 4:**
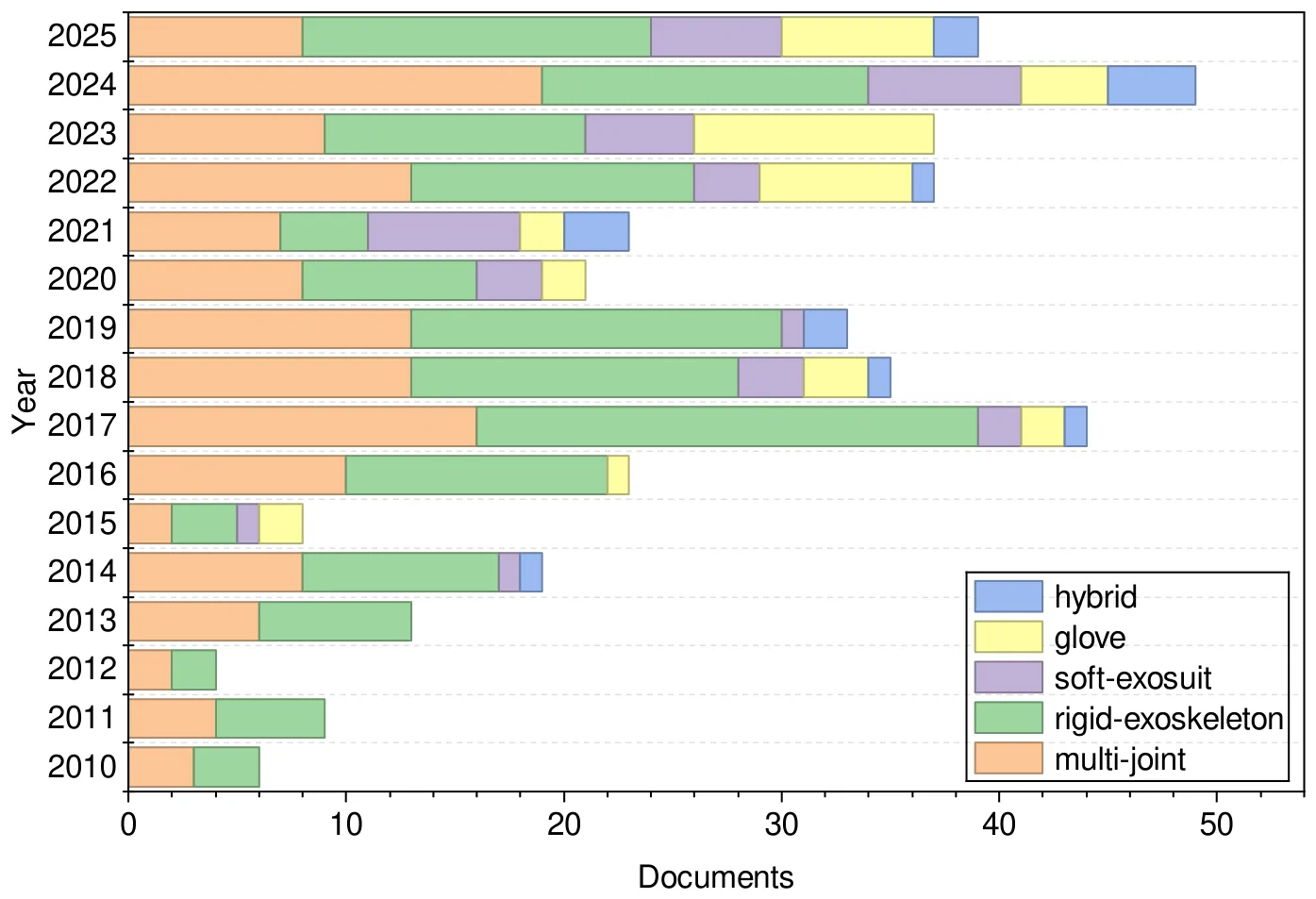
General structure of the exoskeleton.

**Figure 5:**
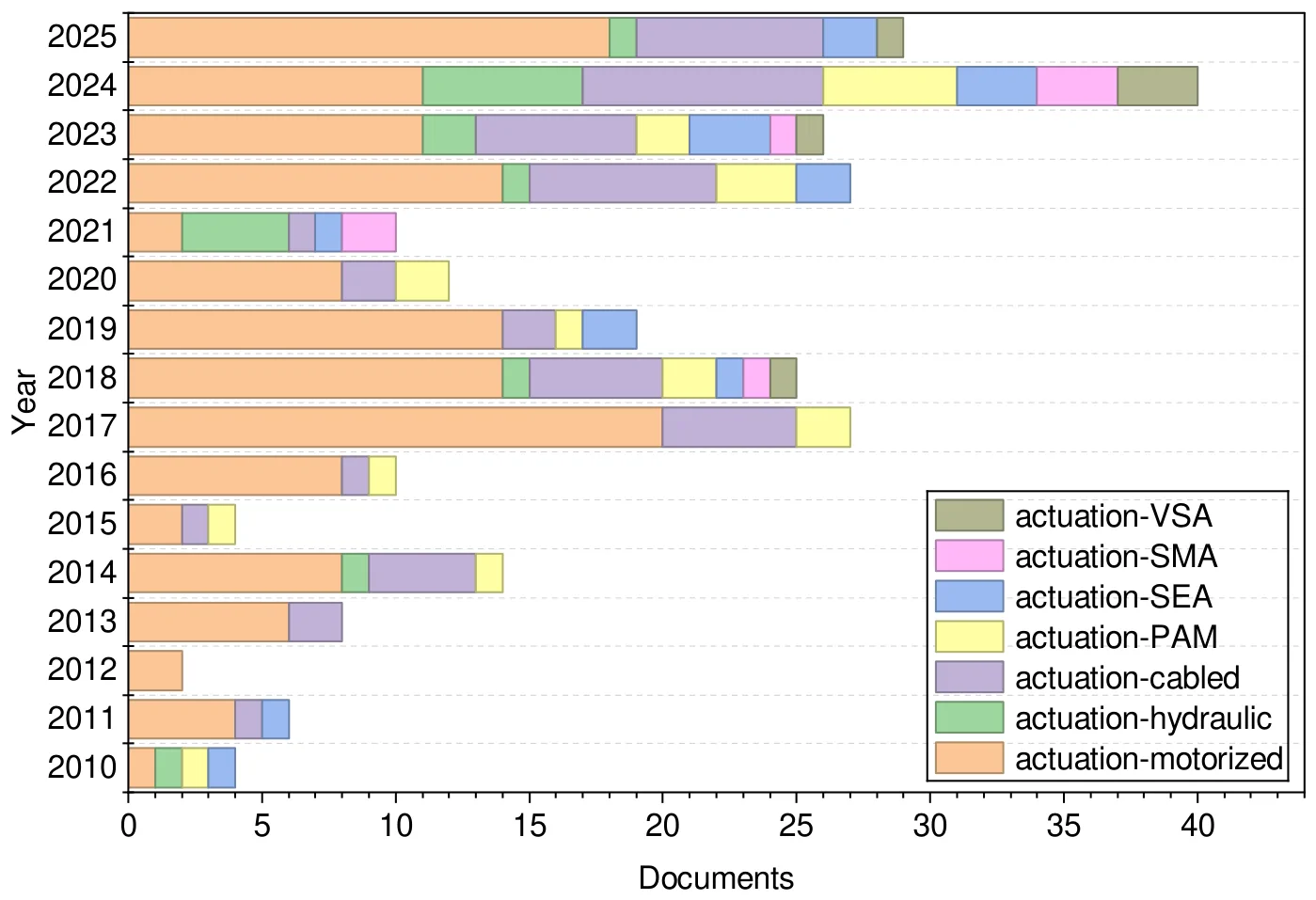
Adopted actuator technologies.

**Figure 6:**
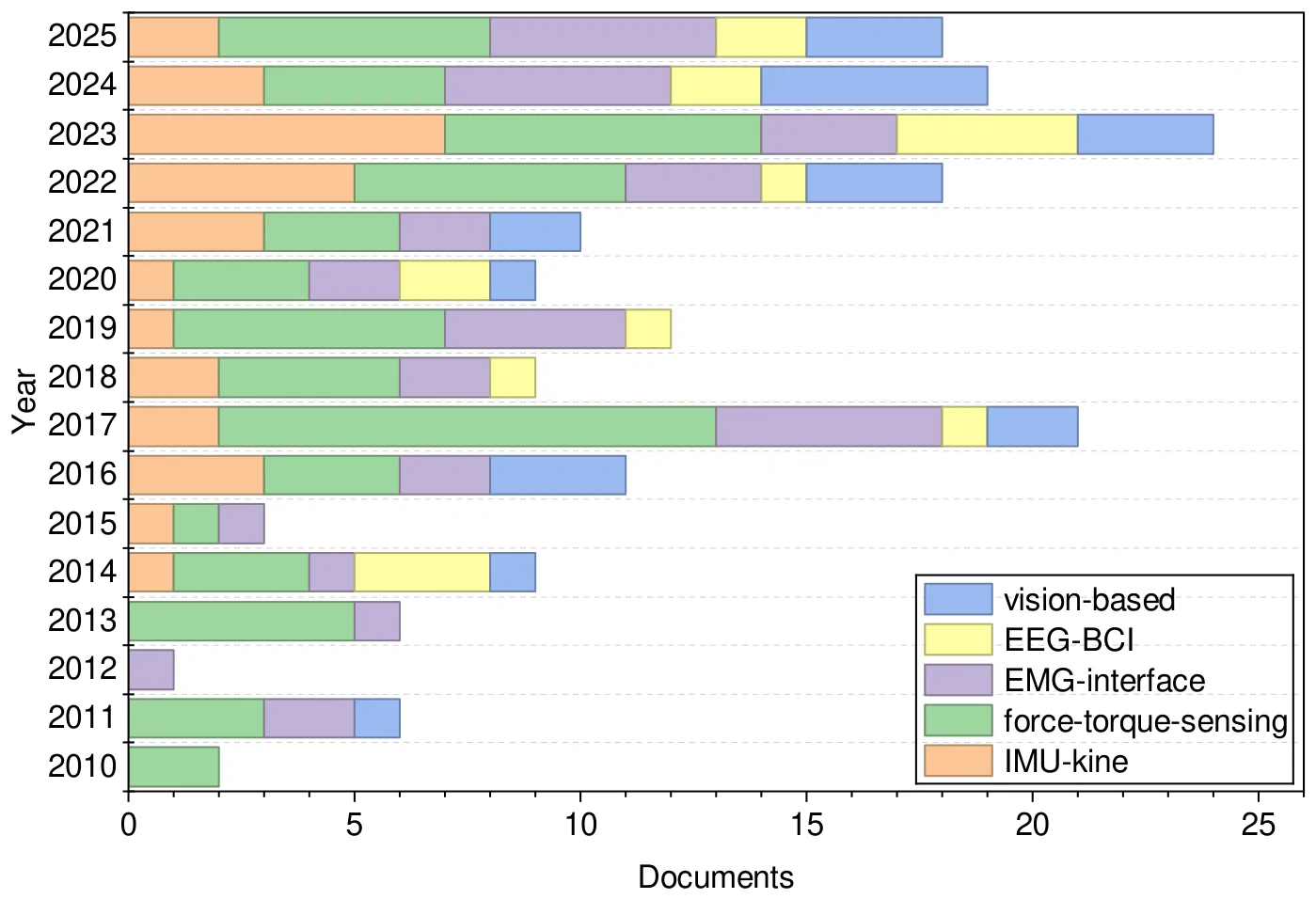
Adopted sensor technologies.

**Figure 7:**
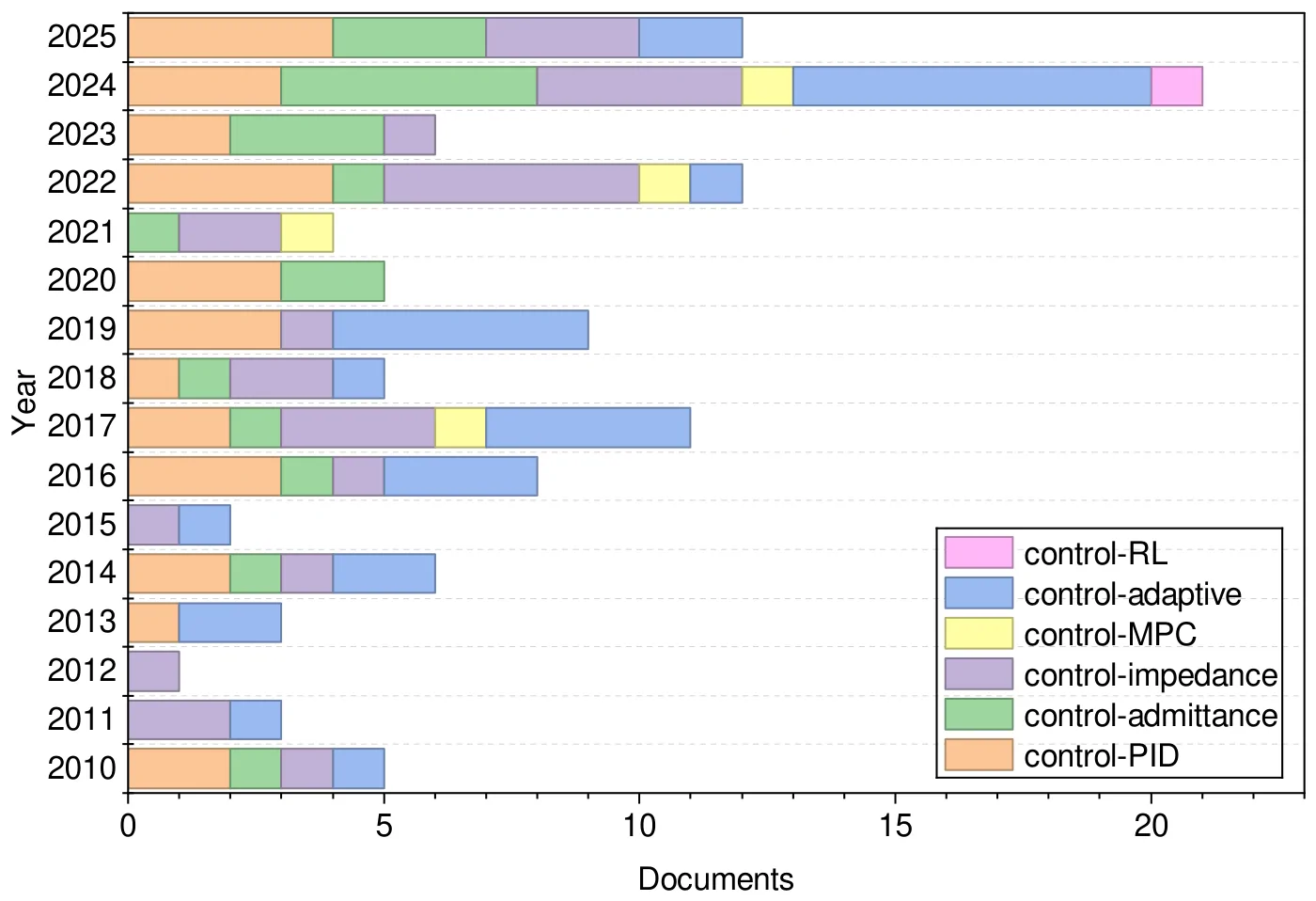
Control techniques.

**Figure 8:**
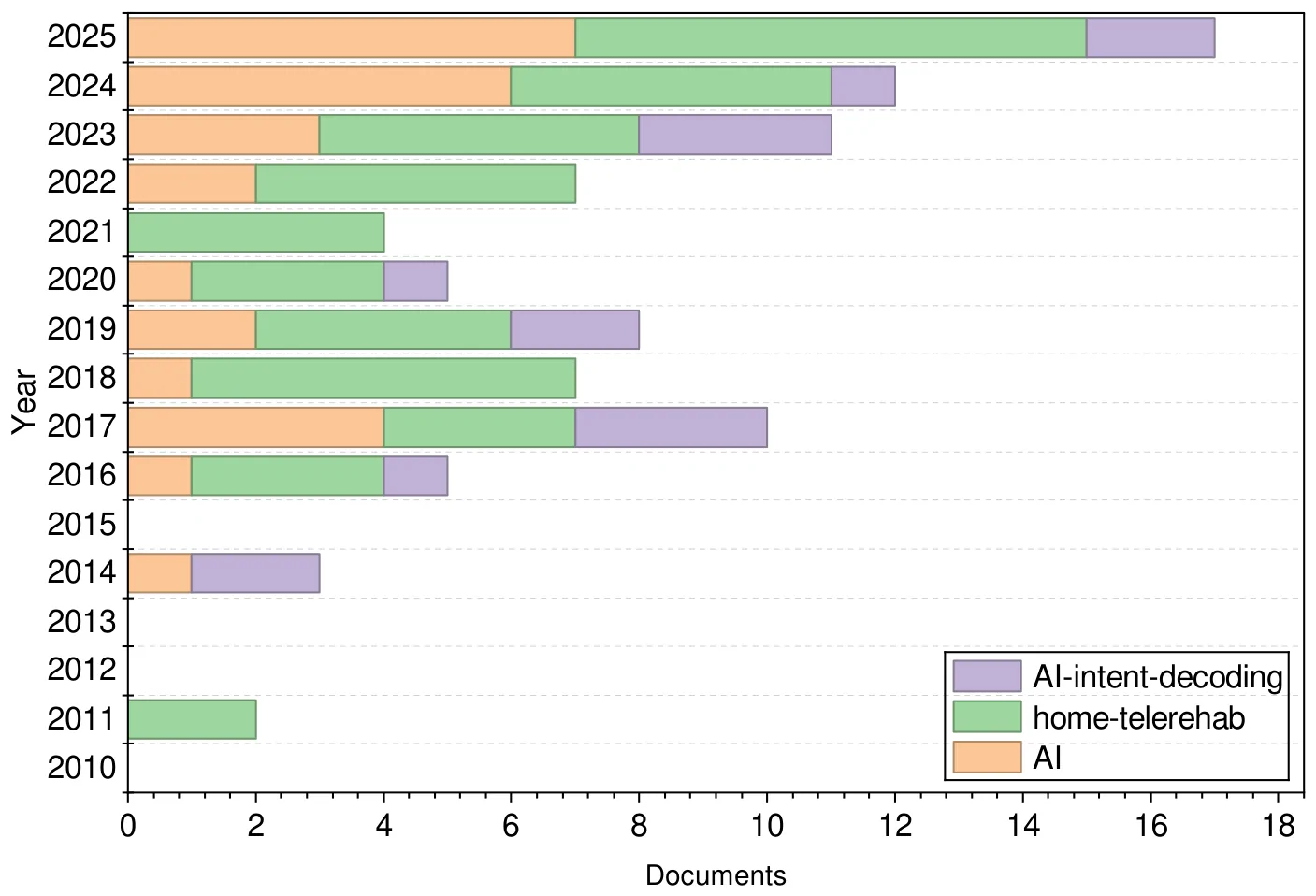
Enabling technologies driving translational innovation.

**Figure 9:**
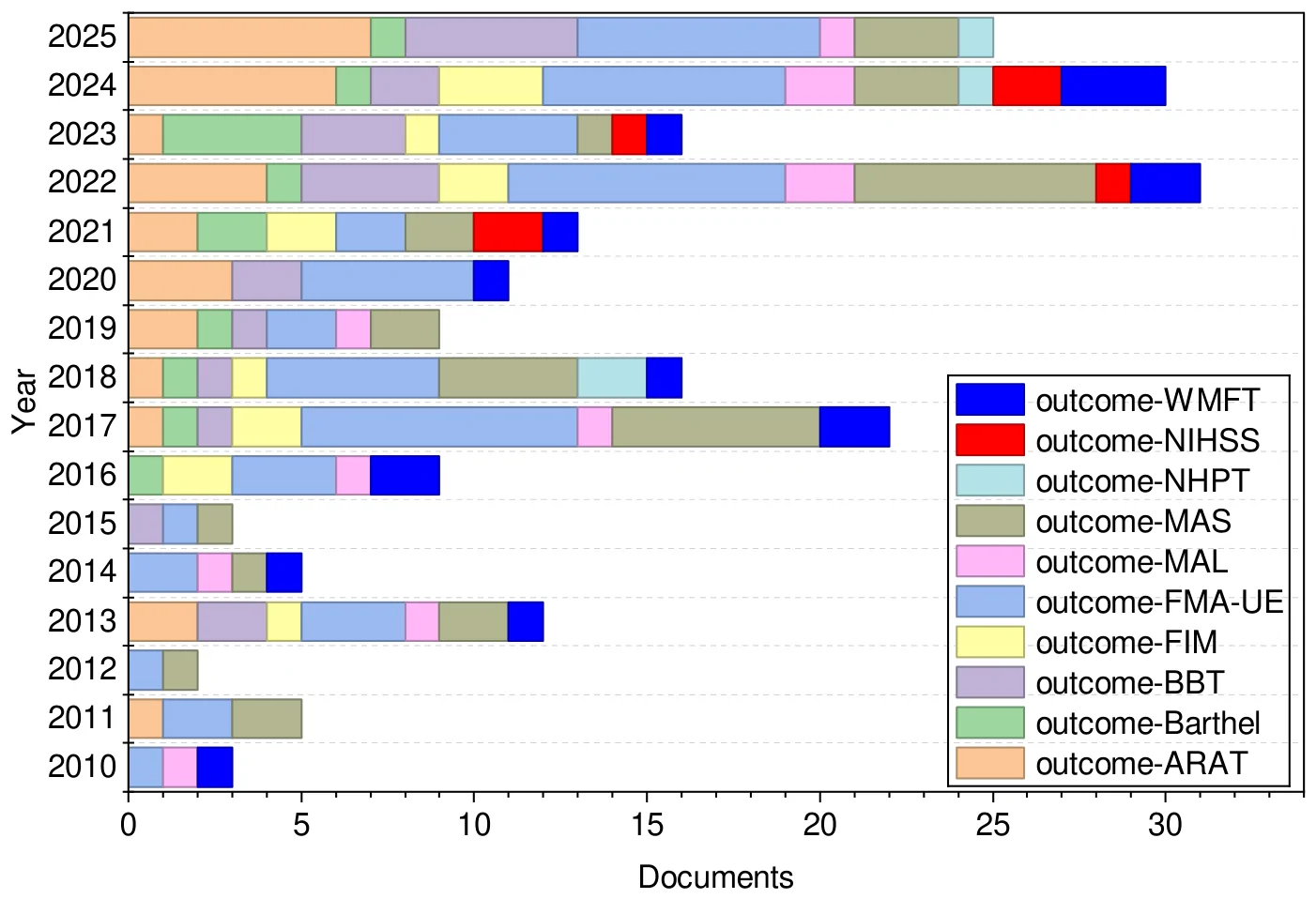
Common medical outcomes.

**Figure 10:**
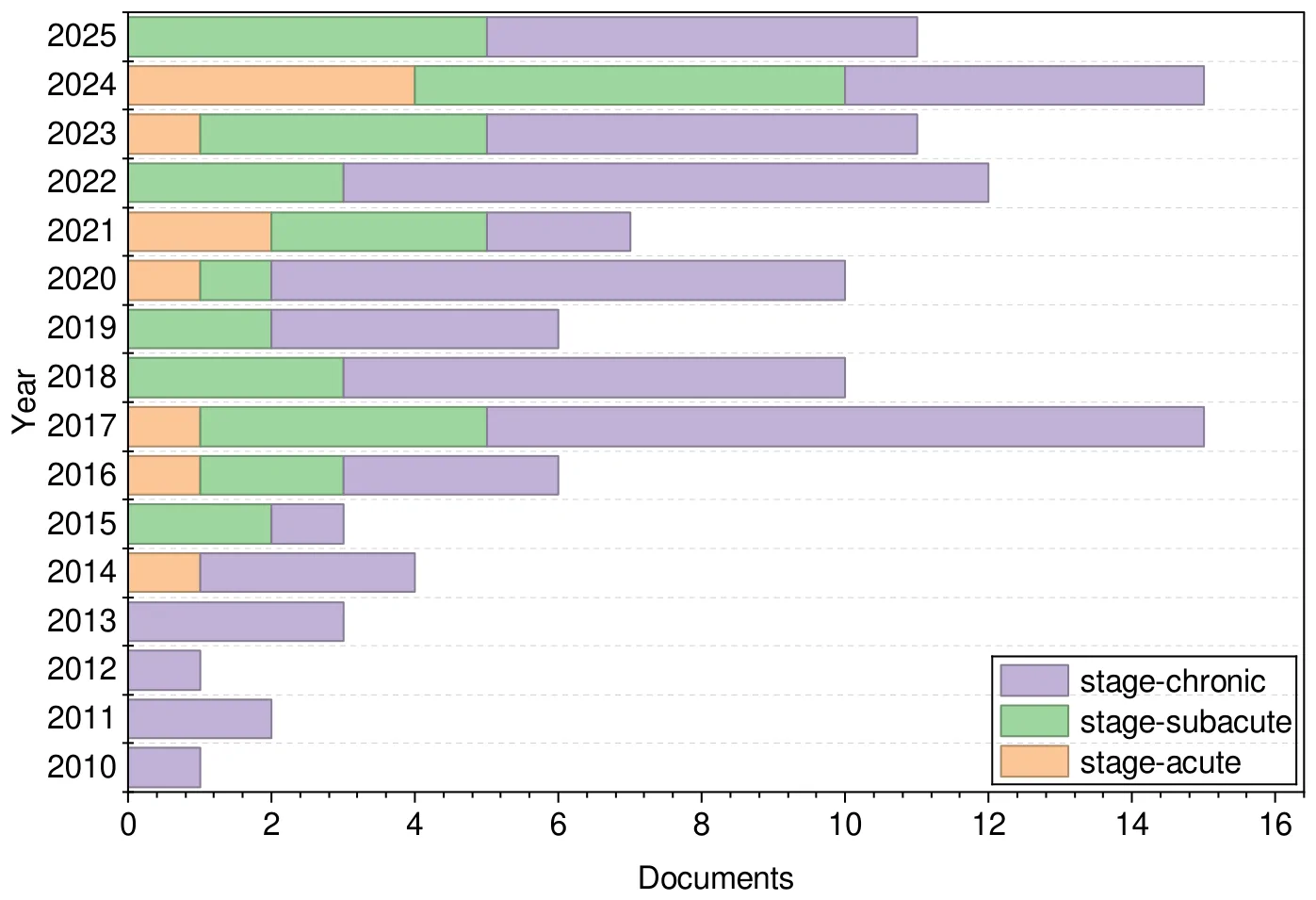
Stages of pathology.

**Figure 11:**
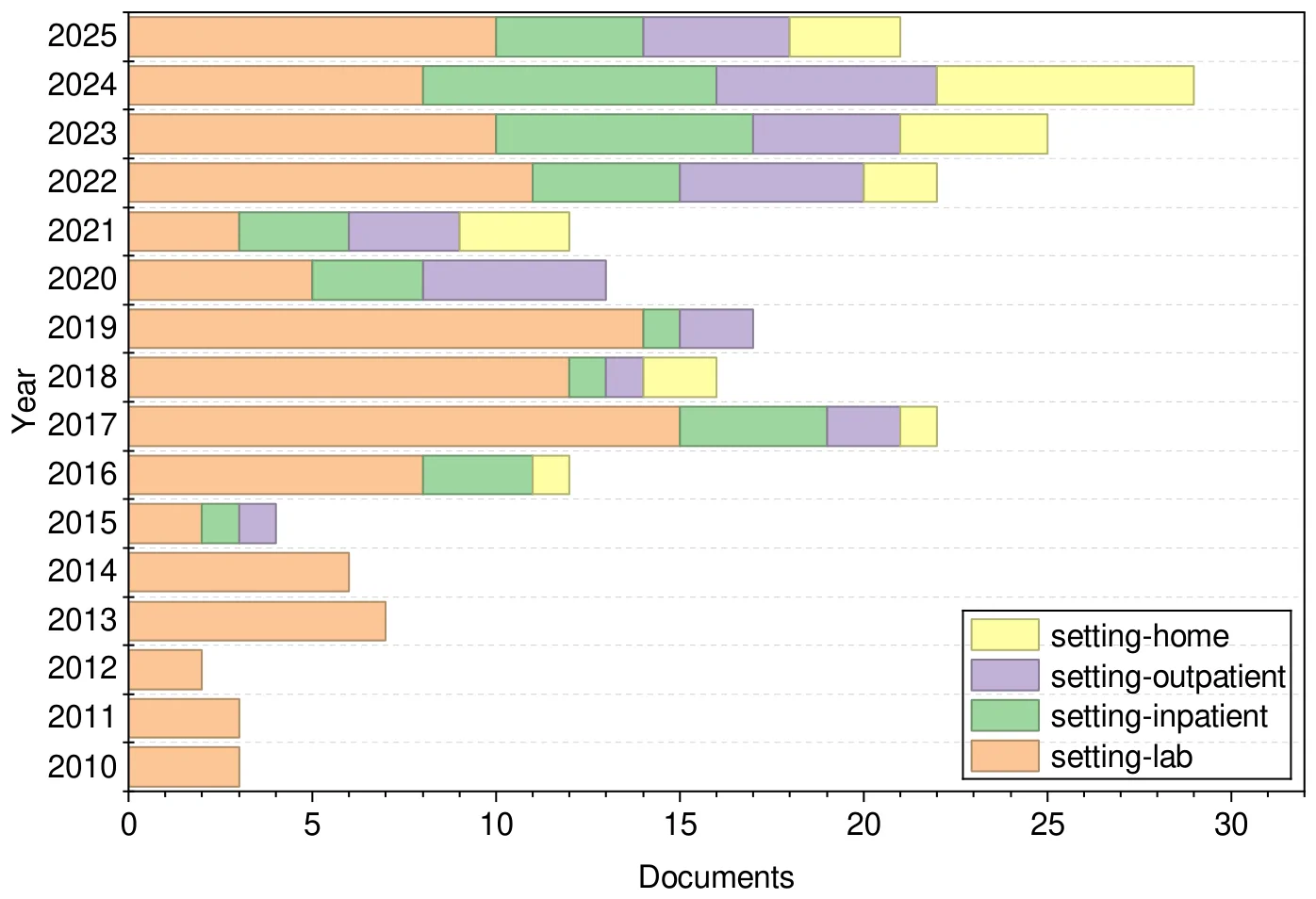
Experimental setting.

**Figure 12:**
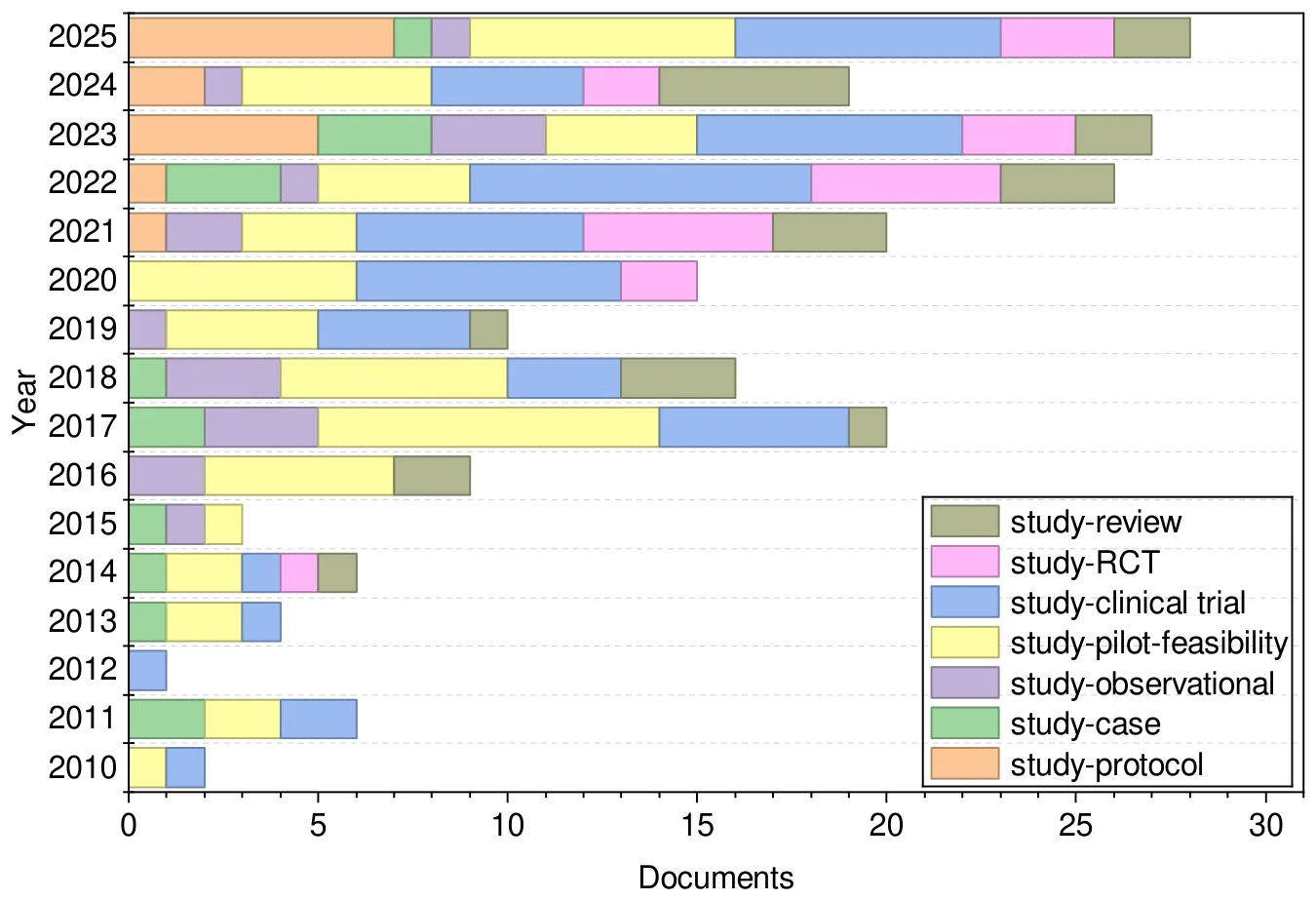
Type of biomedical study.

Figure 3, Figure 4, Figure 5, Figure 6, Figure 7, Figure 8, Figure 9, Figure 10, Figure 11 and Figure 12 graphically represent the trends over time of the technical and biomedical characteristics of the included studies. All counts displayed in these figures represent the number of included studies (out of 219 total) carrying the corresponding label for that year; because a single study may carry multiple labels, the same study may appear in the counts of more than one category within a figure or across figures. Percentages cited in the narrative below are calculated over the 219 included studies. Of the 219 included studies, 16 were RCTs, 42 were non-randomised clinical trials, 16 were observational, 12 were case studies/series, 12 were protocol papers, 22 were reviews/meta-analyses, 68 were technical/engineering studies, and 47 were pilot/feasibility studies (categories are not mutually exclusive). Clinical outcome statements in Section 4.2 are restricted to the RCT and clinical-trial subsets; protocol papers and reviews are cited for context only and do not contribute outcome data. Figure 3, Figure 4, Figure 5, Figure 6, Figure 7 and Figure 8 represent technological characteristics, while Figure 9, Figure 10, Figure 11 and Figure 12 describe biomedical and clinical characteristics. Across all figures, two periods of concentrated scientific production are observable, centred approximately around 2017 and 2024. The two production waves show partial overlap between technical and clinical output, although the clinical production waves are broader and less acute than the technical ones, reflecting the time lag inherent in translating engineering advances into clinical evidence.

Regarding anatomical target (Figure 3), hand and finger segments are the most represented categories across the included studies, accounting for studies in which the device targets the most neurologically dense and functionally critical segments for post-stroke recovery. A notable growth in whole upper-limb coverage—integrating shoulder, elbow, wrist, and hand—is also evident, reflecting the clinical relevance of coordinated reach-to-grasp training. Regarding the overall structure of the exoskeleton (Figure 4), rigid and multi-joint architectures remain the most represented design category, while soft and glove-like structures show substantial growth in recent years, as do hybrid exoskeletons combining robotic and non-robotic elements. Regarding actuation (Figure 5), motorised electric actuation and cable-driven transmission together dominate design choices across the included studies. From a sensing perspective (Figure 6), research is shifting from kinematic and dynamic sensing alone toward the integration of advanced biological intent signals—EMG interfaces, EEG-BCI systems, and vision-based sensing—reflecting the field’s move toward multimodal sensor fusion architectures. From a control perspective (Figure 7), a transition from classical position control toward adaptive and assist-as-needed strategies is evident, though reinforcement learning-based control remains under-represented in this application domain. Enabling technologies—artificial intelligence and telerehabilitation—have grown substantially since 2016 and continue to show marked growth in recent years [75] (Figure 8); the term “exponential” is not used here as no formal growth-curve analysis was conducted, but the trend is clearly accelerating and is likely to drive significant innovation in home-based rehabilitation delivery.

Regarding clinical outcomes (Figure 9), four instruments emerge as the most widely reported across the included studies: the Action Research Arm Test (ARAT), the Box and Block Test (BBT), the Fugl-Meyer Assessment for the Upper Extremity (FMA-UE), and the Modified Ashworth Scale (MAS). The prominence of these four instruments reflects their psychometric robustness and sensitivity to upper-limb motor change, and underscores the importance of selecting outcomes whose measurement level is matched to the device’s anatomical target and therapy goal.

Regarding the stage of pathology targeted (Figure 10), subacute-stage studies are the most represented, consistent with the neurobiological rationale for intensive rehabilitation during the period of peak spontaneous recovery. The substantial representation of chronic-stage studies reflects two complementary rationales: chronic patients are clinically stable and undertreated, making them an ethically appropriate population for experimental intervention; and sustained therapeutic activity in this group may reduce functional decline and improve quality of life—goals that are particularly aligned with home-based, autonomous exoskeleton use enabled by AI and telerehabilitation. Acute-stage studies are growing but remain limited, as clinical stabilisation priorities and the complexity of the acute care environment present barriers that are often difficult to overcome with current exoskeleton systems.

Regarding experimental setting (Figure 11), laboratory-based studies predominate, which is expected in a field still characterised by a large proportion of technical validation and early feasibility work. Growth in outpatient and home settings is evident in more recent years, consistent with the enabling technology trends described above. Regarding study design (Figure 12), a progressive increase in structured study types—clinical trials and RCTs—is observed over the review period, alongside a notable recent increase in protocol publications. This latter trend reflects the field’s move toward more rigorous pre-specified evaluation frameworks and is associated with the development of personalised rehabilitation paradigms enabled by adaptive control and AI-driven systems.

## 4. Discussion

As detailed in the Methods and summarised in Table 1, the evidence base is heavily weighted toward technical/engineering and pilot-feasibility studies (68 and 47 of 219 included studies, respectively), with RCTs (16) and controlled clinical trials (42) forming a minority; non-randomised and non-blinded designs predominate even within the clinical subset. Clinical effectiveness findings reported by the included studies—and synthesised here as part of this narrative review—should therefore be regarded with cautious optimism; methodological biases may inflate reported treatment effects, and the conclusions drawn reflect the state of published evidence rather than independent statistical reanalysis.

Sensing is the analytical thread connecting the two halves of this Discussion: Section 4.1 examines how sensing modalities are implemented, validated, and fused at the device level, while Section 4.2 examines how sensor-derived and clinician-rated measures are used to assess outcomes.

### 4.1. Engineering and Technical Aspects

Technical research on upper-limb and hand exoskeletons provides the engineering foundation for clinical translation [76]. The literature organises around four pillars: (i) mechatronic architecture and actuation; (ii) sensing and user-intent detection; (iii) control for safe and effective human–robot interaction (HRI); and (iv) bench-level assessment of performance, reliability, and usability constraints (weight, size, battery life) [45,46,62,63,77,78,79,80,81]. A common design objective is achieving devices light enough for distal placement yet capable of generating sufficient torque and range of motion for functional training. Research emphasises lightweight and adaptable materials (aluminium, soft fabrics), modular frames, and affordable architectures that support deployment to a wider patient population, while navigating the fundamental trade-off between mechanical complexity and manufacturability [55,56,60,61,80,82,83,84]. Cable-driven remote actuation—particularly Bowden-cable approaches that relocate motors away from the limb—has grown substantially as a strategy to increase wearability and reduce distal inertia. Representative implementations describe bidirectional pull-pull Bowden configurations with pretensioning mechanisms and spool diameter ratios (e.g., 2:1 to 3.2:1) chosen to amplify joint torque, with transmission efficiency shaped primarily by friction and sheath curvature [73,79,81,85,86]. Multi-DOF wearable systems increasingly prioritise anatomically aligned and ergonomically fitted designs, particularly at the shoulder, where remote centres of rotation and passive self-alignment mechanisms reduce joint misalignment and improve comfort [78,87,88,89,90,91]. Intent detection and assistance adjustment represent a second major technical pillar. Two families of intention recognition are well established: bio-signal-based methods (EMG and EEG) and interaction-force-based methods. EMG is framed as a feedforward signal mappable to movement intent through linear or nonlinear musculoskeletal models, enabling effort-coupled control pipelines. EEG-based control extends intent decoding to severely impaired patients but faces documented practical barriers—inter-subject variability, noise sensitivity, and calibration burden [47,48,49,50,51,52,92,93,94,95]. Force/torque sensing at the human–robot interface provides a more direct route to intuitive control with lower decoding complexity, supporting impedance and admittance HRI controllers. Deep learning-based EMG decoding for shared control is an emerging approach within the broader trend toward multimodal sensing and inference [91,96,97,98]. Control-focused studies increasingly address the safety of physical interaction. For cable-driven architectures, transforming joint-space errors into cable-length errors enables PID-style control in the actuator domain with cable-tension monitoring to detect slack. Compliant control methods—most notably admittance control—limit interaction forces and reduce secondary injury risk, particularly when spasticity prevents the limb from reaching the desired posture [15,99,100,101]. Assist-as-needed (AAN) paradigms avoid over-assistance by continuously adjusting support to the user’s active contribution, modulating impedance and controller parameters to sustain engagement while ensuring task completion [15,84,102,103,104,105,106]. Bench testing provides quantitative validation of actuator output and functional grasp capability prior to human trials [107]; representative examples characterise flexion actuator performance via load-cell tip-force measurements and universal testing machine grip-force assessment, separating frictional and normal force contributions to establish safety and control limits [78,85,99,108]. Reliability and autonomy are increasingly treated as first-class engineering objectives, particularly for home-use concepts [109,110]: accelerated life testing reveals failure modes associated with gearbox wear at high duty cycles, and battery testing under loaded conditions maps torque demand to practical autonomy, consistently showing that real-world duty cycles are lower than laboratory benchmarks. Relocating control and power units to backpack or waist-mounted packs reduces distal mass while explicit component cost reporting signals that comfort, maintainability, and affordability are being treated as engineering requirements alongside biomechanics and control [63,77,111,112,113,114,115,116].

#### 4.1.1. Target Articulation

Upper-limb exoskeletons can be classified at joint-level based on their target articulation, distinguishing devices designed for hand/finger, wrist, elbow, shoulder, and full-arm assistance.

##### Hand and Finger Exoskeletons

Hand and finger exoskeletons (often implemented as robotic gloves [117,118,119]) prioritise portability, safety, and affordability, driven by the kinematic complexity of the hand and the prevalence of post-stroke weakness and spasticity [41,57,58,59,68,120,121,122]. A consistent trend in the reviewed literature is the choice to assist gross grasp/release over individual finger dexterity, reflecting clinical recovery trajectories in which global hand function typically precedes fine motor control [31,77,121]. Soft or semi-soft glove architectures with cable/tendon routing and remote actuation packs have emerged as a practical solution to minimise distal inertia and address joint alignment variability [123,124,125,126,127,128,129,130]. A key design challenge is the non-fixed centre of rotation (COR) of the finger joints [131]: misalignment between the device and anatomical CORs generates parasitic loads and discomfort, and is addressed either through CT-informed equivalent rotation centres or underactuated linkage configurations [132,133,134,135] that passively follow the natural motion of the hand [136,137,138]. The literature increasingly links design decisions directly to clinical outcomes, highlighting the need to stratify assistance by spasticity level, standardise outcome reporting, and prioritise usability for home and community use [31,77,80,84,113,121,122,136,138,139,140,141,142,143,144,145,146,147].

##### Wrist Exoskeletons

The wrist is commonly modelled with two active DOFs (flexion/extension and radial/ulnar deviation), with forearm pronation/supination typically incorporated as part of a larger elbow/forearm module [148,149,150]. A central challenge is kinematic compatibility: the anatomical wrist axes vary across subjects and post-stroke conditions, requiring designs to either adopt redundant passive DOFs (as in the RiceWrist-S) or flexible cable-driven approaches that are inherently tolerant of joint axis misalignment [93,112,151]. An emerging trend is the integration of wrist modules within full-arm platforms to enable coordinated, multi-joint task-relevant training across the shoulder–elbow–wrist chain [152] as it can be seen in CAREX-7 [21,151,153] and in RehabRoby [99,154,155].

##### Elbow Exoskeletons

Elbow exoskeletons typically implement a single primary flexion/extension revolute DOF. The dominant design challenge is joint compatibility: since the exoskeleton is mounted at multiple contact points, misalignment between the mechanical and anatomical axes generates interaction forces that compromise safety and comfort [87,89,149,153,156,157]. Passive self-alignment joints—either free or lockable—have been shown to substantially reduce these interaction forces [87,157]. Cable-driven architectures represent an alternative approach that reduces sensitivity to alignment errors while maintaining a lightweight profile [73,79,89,99,157,158]. Beyond simple flexion/extension, control strategies targeting shoulder–elbow coordination (e.g., resistive modes that discourage compensatory shoulder substitution during reach) have demonstrated reduced biceps co-activation and more normalised movement patterns [98,159,160].

##### Shoulder Exoskeletons

The shoulder is the most mechanically complex segment, requiring exoskeletons to replicate the coordinated motion of the sternoclavicular, acromioclavicular, and glenohumeral joints. Standard design practice separates the scapula/girdle mechanism (elevation/depression, protraction/retraction via parallelogram linkages) from the glenohumeral mechanism (three serial revolute DOFs), with non-orthogonal joint axis orientations used to enlarge the usable workspace and avoid singularities near the head [87,89,161,162]. Including passive accommodation or redundant DOFs at the shoulder girdle is a well-established design principle to reduce parasitic interaction loads [87,88]. Full-arm platforms such as Harmony (five active shoulder DOFs, SEA actuation) and CAREX-7 (cable-driven, alignment-tolerant) represent contrasting philosophies [163]—high kinematic fidelity versus mechanical simplicity—both of which are viable depending on clinical and deployment context [15,85,156,164,165,166,167,168].

##### General Arm Exoskeletons

Full-arm devices integrate shoulder, elbow, and frequently wrist modules to enable coordinated multi-joint training. A major design tension exists between functional coverage (number of active DOFs) and portability [169,170,171,172,173,174,175,176]: each active joint adds mass, making fully wearable systems difficult to achieve [77,78,88,89,149,177,178,179,180]. Emerging designs address this through hybrid architectures combining a small number of active joints with strategically placed passive or lockable DOFs and remote actuation via cable transmission to reduce distal inertia (e.g., PULExo, UULE) [73,78,181]. Gravity compensation [182,183]—whether through counterweight mechanisms or model-based feedforward torque—is a near-universal feature at the shoulder and elbow and constitutes a prerequisite for safe home-based use [77,184,185]. Growing interest in bimanual and hybrid neuro-exoskeleton systems (combining robotic assistance with FES and intent decoding via EMG/EEG) signals a shift from isolated joint exercises toward coordinated, task-relevant paradigms [5,15,97,100,186,187,188,189,190,191,192].

#### 4.1.2. Structure of Exoskeletons

Exoskeleton structure—the physical architecture that defines how forces are transmitted to the user—is a primary determinant of clinical deployability, kinematic compatibility, and rehabilitation efficacy. The reviewed literature converges on four structural categories: multi-joint, rigid, soft/glove-based, and hybrid.

##### Multi-Joint Exoskeletons

Multi-joint platforms spanning the shoulder–elbow–forearm–wrist chain enable task-oriented, coordinated training rather than single-joint isolation, which is more aligned with neuroplasticity principles and functional recovery goals [77,87,88,149]. The dominant design tension is between functional coverage (number of active DOFs) and portability: each additional actuated joint adds mass and bulk, and most multi-DOF devices remain wheelchair-mounted or grounded [77,78,184]. Emerging solutions address this through hybrid kinematics—combining a small number of active joints with strategically placed passive or lockable DOFs and remote actuation—to reduce distal inertia while preserving clinical utility [73,77,78,168]. A further consistent finding is that constraining passive self-alignment joints increases parasitic interaction loads, while permitting passive motion substantially reduces them [193,194]—a principle with direct implications for both comfort and safety in multi-joint architectures [77,87,88,149].

##### Rigid Exoskeletons

Rigid link-and-joint structures provide precise, geometrically defined motion and support high-fidelity sensing (IMU, EMG, force/torque), but their mass, volume, and kinematic incompatibility with inter-subject anthropometric variability represent persistent barriers to wearability and home deployment [149,184,185]. The central design challenge is achieving anatomical compatibility: the mismatch between mechanical and biological joint axes generates cuff forces and discomfort, particularly at the shoulder and wrist. Solutions include passive self-alignment DOFs that absorb misalignment, and adjustable linkage geometries [87,157,195]. A broader clinical concern is that historically rigid systems imposed predetermined passive trajectories (analogous to CPM), which limits neuroplastic benefit; current best practice instead integrates compliant actuation elements (e.g., variable stiffness actuators and series elastic elements) that support active patient participation and allow stiffness to be used as a clinical parameter adjusted across rehabilitation stages [15,31,33,102,195,196,197,198,199,200,201].

##### Soft Exoskeletons and Gloves

Soft exosuits and robotic gloves use compliant textile or fabric substrates—rather than rigid linkages—to transmit assistive forces, directly addressing the weight, misalignment, and wearability limitations of rigid structures [77,202,203]. Within this category, two actuation strategies dominate: pneumatic and cable/tendon-driven. Pneumatic gloves offer high inherent compliance at the hand interface but require bulky auxiliary equipment (compressors, valves, tubing) that limits portability and reduces actuation precision [125,144,204]. Cable-driven gloves route tendons along a soft substrate and relocate motors proximally or externally, achieving lower distal mass, greater comfort, and a clear palmar surface for object interaction—at the cost of careful routing, anchoring, and fit management to avoid joint reaction forces [181,202,205]. Representative cable-driven platforms include Exo-Glove, HandCARE, and Gloreha [77,181,205]. A common simplification strategy treats the four fingers as a single actuated unit and controls the thumb separately, reducing the motor count from five to two and minimising hand-mounted mass [80,125,205]. Recent developments have extended bidirectional actuation (flexion and extension support) and full-textile construction to soft gloves, with auxiliary thumb splints addressing the common post-stroke limitation of thumb opposition [124,136,144,206,207]. For multi-joint coverage, Bowden cable-based exosuits route power through compact paths to deliver coordinated assistance across shoulder, elbow, wrist, and fingers within a textile garment, with back-mounted actuation units keeping mass away from distal segments [168,181,202,205,208].

##### Hybrid Exoskeletons

Hybrid systems combine robotic exoskeletons with functional electrical stimulation (FES), exploiting the complementary mechanisms of each modality [209,210,211]: proximal robotic assistance primarily targets body structure and function recovery, while distal FES promotes activity- and participation-level gains [4,77,102,212]. The exoskeleton component may be active (motor-driven) or passive (gravity compensation/spring-loaded), with FES applied either at the same joint as the robot or distally while the robot manages the proximal limb [13,77,102]. Examples of hybrid systems that have been developed include: (i) Hand/Grasp assistance through the exoskeleton with hand assistive FES [213] (HEXA FES for grasping), (ii) Proximal Robot + Distal FES to enable reaching and grasping/manipulation, and (iii) Hand-focused devices with hybrid FES–Robot Assisted Hand Training that were tested in subacute populations. A variety of tasks have been assisted including Finger Flexion/Extension, Wrist Flexion/Extension, Elbow Flexion/Extension, and Integrated Reach-To-Grasp Object Interactions, see [31,102,153,214,215]. Intent decoding ranges from kinematic threshold triggering to EMG-driven activation to BCI-mediated command pathways, with more advanced systems incorporating multi-modal fuzzy or adaptive controllers that synchronise electromechanical and neuromuscular actuation in real time [102,111,216].

#### 4.1.3. Actuation Devices

Actuator selection is a primary determinant of portability, safety, controllability, and clinical deployability, as it dictates (i) the achievable torque/force bandwidth, (ii) distal mass distribution, and (iii) complexity of the power supply chain. Seven actuation families are represented in the reviewed literature: electric motors, hydraulic actuators, cable/tendon drives, pneumatic artificial muscles, series elastic actuators, shape-memory alloy actuators, and variable-stiffness actuators.

##### Electric Motors

Electromechanical actuation dominates the field due to the precision, programmability, and versatility of DC/brushless motors across passive, active-assisted, and resistive therapy modalities [89,149,184,185]. Multi-DOF platforms typically pair brushless DC motors with high-ratio reducers (most commonly harmonic drives) at each joint to achieve high continuous torque in a compact form factor [89,153,184]. At the distal end (forearm–wrist–hand), hybrid arrangements combining direct drive (for low-friction joints such as forearm pronation/supination) with cable transmission (for wrist and finger joints) are preferred to minimise backlash and reduce distal inertia [93,108,112,148,151]. In hand exoskeletons, tendon routing with motors located on the forearm or in a waist-mounted pack is the standard strategy to achieve a lightweight, open-palm glove suitable for prolonged use [149,160,217].

##### Hydraulic Actuators

Hydraulic actuation offers high power density and torque output but has seen limited adoption due to the integration penalties of the required supply unit, plumbing, valves, and regulators—penalties that are particularly prohibitive in post-stroke rehabilitation where donning time, therapist workflow, and home deployment are key design constraints [77,149,155,184,217,218,219,219]. Hydraulic systems have been applied mainly to grounded multi-DOF upper-arm platforms (e.g., LIMPACT, a 5-DOF shoulder–elbow device) rather than distal hand devices, where the bulk of ancillary hardware becomes even less acceptable [31,77,184]. The incorporation of series elasticity into hydraulic actuators (rotational hydroelastic actuators, series elastic hydraulic motors) has been explored to improve torque control and reduce vibration but has not overcome the fundamental portability barrier [149,160,217].

##### Cabled Actuators

Cable-driven transmission enables remote motor placement, reducing distal mass and improving wearability—both critical requirements for upper-limb rehabilitation [73,181,184,218]. Early designs used cable-pulley routing on rigid frames (e.g., MEDARM, CADEN-7, IntelliArm), which scaled poorly with DOF count and limited anthropometric adjustability [85,181,205,220]. Bowden cable transmission has become the preferred approach in recent designs, decoupling actuator placement from joint location, enabling freer link-length adjustment, and facilitating ergonomic wearable architectures [73,85,181]. A bi-directional pull-pull Bowden arrangement with spool diameter ratios provides torque amplification while maintaining cable pre-tension and transmission efficiency [73,78,184]. Known limitations include nonlinear tension behaviour with posture changes and sensitivity to routing, anchoring, and glove fit—particularly relevant in post-stroke populations where spasticity increases interface loads [73,85]. For the hand, underactuated cable routing that couples multiple fingers through shared tendons reduces motor count and mass while preserving functional grasp/release capability [149,218,221,222].

##### Pneumatic Artificial Muscle (PAM) Actuators

Pneumatic Artificial Muscles (PAMs) offer inherent mechanical compliance and a muscle-like force-contraction profile, making them attractive for safe human–robot interaction. However, the requirement for compressed air supply, valves, and regulators adds bulk and complexity that undermines portability, and the low bandwidth and nonlinear pressure-force relationship complicate closed-loop control [31,184,218,223]. PAMs have been applied both as standalone actuators in fabric-based elbow exosuits and in combination with parallel closed-chain mechanisms at the wrist to partially recover positioning stiffness [168,204,207,218]. The recurring critique is that PAM-based systems may be lightweight at the point of attachment but remain system-level non-portable due to the peripheral hardware, a limitation that remains unresolved in current prototypes [4,123,184,218,224,225]. Recent developments in PAM architecture have addressed the wearing comfort limitation directly: an asymmetric expansion pouch PAM achieves an 89.61% reduction in skin-side extrusion force compared to conventional rectangular pouch configurations while maintaining a contraction force of 243.94 N at 70 kPa and a contraction force-to-weight ratio of 27,100 N/kg, representing a promising direction for wearable actuation in rehabilitation contexts [224]. Adjacent engineering developments in pneumatic actuation for manual therapy offer design insights on pressure regulation and soft-contact mechanics that, while outside the stroke-rehabilitation eligibility scope of the present review, remain relevant to the broader pneumatic soft-robotics design space [225].

##### Series Elastic Actuation (SEA) Devices

Series Elastic Actuators (SEAs)—which place an intentional elastic element between the power source and the joint—are widely used in upper-limb rehabilitation exoskeletons to improve interaction safety, absorb spasticity-induced shocks, and enable torque-regulated control strategies (impedance, admittance, and assist-as-needed) [77,184,218]. SEAs appear in both rotary form (compact brushless motor + harmonic drive + torsion spring, as in the Harmony system) and linear form (ball-screw-based, which also provides mechanical range-of-motion limits as a safety feature) [184,217,218,226,227]. The primary drawback is added mass from the spring and mechanical components; this is mitigated by combining SEAs with Bowden cable transmission to locate the actuator package proximally while transmitting forces distally via cables [79,184,226]. The SEA principle extends across energy domains: hydraulic series elastic motors (LIMPACT) and cable-path series springs (UP-CARE) demonstrate that compliance-in-series is a general design pattern rather than a technology-specific one [200,228,229,230].

##### Shape-Memory Alloy Actuation (SMA) Devices

SMA actuators—typically NiTi (Nitinol) wires that contract upon thermally triggered martensitic-to-austenitic phase transition—are attractive for their compactness, low mass, and compatibility with tendon-like routing configurations, making them candidates for highly wearable hand and elbow devices. Bowden cable transmission is commonly used to route SMA wires away from the joint, reducing distal inertia while preserving a flexible, compliant interface with the limb. However, SMA actuation carries well-documented limitations that are particularly problematic in post-stroke rehabilitation: nonlinear hysteretic behaviour, strong temperature dependence, limited bandwidth due to heating/cooling cycle times, and high energy consumption. These characteristics make closed-loop torque/position tracking difficult, compromise transparency and impedance modulation, and raise safety concerns around thermal management near the skin during extended sessions. Current SMA-based prototypes remain largely at the proof-of-concept stage, and clinical adoption will require advances in thermal design, embedded sensing, and control strategies that explicitly address hysteresis and rate dependence [184,218,231,232,233,234,235,236].

##### Variable-Stiffness Actuation (VSA) Devices

VSAs extend the SEA concept by making the compliance of the actuating chain actively adjustable, enabling the mechanical impedance of the exoskeleton to be tuned as a clinical parameter across rehabilitation stages [160,199]. The clinical rationale is well established: higher apparent stiffness assists path-following in early recovery when voluntary control is limited, while progressive stiffness reduction challenges the patient and encourages voluntary contribution as motor function improves—a process directly associated with neuroplastic adaptation [160,199]. Representative VSA implementations in upper-limb exoskeletons achieve stiffness modulation via cable tension adjustment through cam-driven mechanisms and self-locking worm gears, the latter reducing continuous power consumption—an important feature for portable devices [184,199]. Practical challenges remain: increased mechanical complexity, reliable stiffness estimation, and limited bandwidth in slow cam-based adjustment mechanisms are barriers to clinical adoption that require further validation [92,97,168,237].

#### 4.1.4. Sensors

The sensing layer is the primary enabler of the feedback loop between device state, biomechanical human–robot interaction, and voluntary user intent [22,238]. Its function spans three coupled roles: measuring the kinematic and kinetic state of the device and limb; detecting and quantifying physical interaction forces at the human–robot interface; and decoding the user’s voluntary intent and physiological engagement to enable active participation in therapy. Researchers have increasingly stressed the importance of integrating high-resolution sensory data—combining kinematic, force, and biological signal modalities—to enhance both control performance and the evaluation and tracking of rehabilitation outcomes [168,239,240]. The field is converging on multimodal fusion architectures because each individual sensing modality carries inherent limitations that degrade performance in the post-stroke population; fusion across complementary modalities compensates for the weaknesses of each [92,97,168,237]. These considerations—sensor validation against reference standards and the sensor-driven control strategies built on top of them—are addressed modality-by-modality below and synthesised in Table 2.

##### Inertial Measurement Units

IMUs are the most widely deployed sensing modality in wearable upper-limb exoskeletons, valued for low cost, low weight, and ease of integration [149,218,241]. Their primary roles are: (1) joint and segment angle estimation for feedback control and contralateral mirroring therapy, where the healthy-limb angle drives the reference trajectory for the impaired side [103,242,243,244]; (2) safety monitoring via posture-threshold logic [111,245,246]; and (3) low-burden user interfaces using orientation or acceleration events (e.g., tapping gestures) to command assistance—particularly relevant for home use where external tracking infrastructure is unavailable [145,168,214,247,248]. Practical deployment requires careful calibration (bias correction, gravity alignment, and axis registration) and drift management through multi-sensor fusion, as mounting variability and soft-tissue artefacts can corrupt the signal. Sampling rates in deployed systems typically range from 100 to 400 Hz, with sensor fusion filters (Madgwick, Mahony, or extended Kalman filters) used to maintain angular accuracy under dynamic conditions [145,241,247,249].

##### Force and Torque Sensors

Force/torque (F/T) sensing underpins safe physical human–robot interaction (pHRI) and enables compliance-based control strategies (impedance/admittance, assist-as-needed) and quantitative therapy dosage monitoring [15,93,198]. Implementation approaches include multi-axis wrench sensors at the cuff or handle, joint-level torque sensors for model-based control and transparency, and local pressure sensors at individual phalanges to detect kinematic mismatch and prevent skin injury [73,84,97]. Current-based torque estimation is insufficient at high frequencies due to noise and friction uncertainty; direct joint torque sensing is preferred for model-based transparency control [97,159,250,251]. In cable-driven systems, tendon tension measurement is theoretically attractive but practically complicated by transmission nonlinearities—preload, friction, and cable bending—that impede reliable joint-torque mapping [73,226,252]. For home deployment, miniaturised strain-gauge or capacitive load cells integrated into garment or orthosis structures represent the direction of current development, trading measurement range for form factor and donning simplicity.

##### Electromyographyc Sensors

Surface EMG is the most researched biological intent signal for exoskeleton control, enabling effort-coupled assistance that promotes active patient participation [77,93,149,218]. Three interface strategies are represented: (1) threshold-based event triggering, initiating discrete assistance phases on muscle activation detection—computationally simple and clinically interpretable; (2) continuous proportional myoelectric control, mapping EMG amplitude to assistive torque or velocity reference for graded effort-scaled assistance; and (3) pattern recognition and deep learning-based decoding (CNNs, LSTMs) for multi-grasp classification, used in shared-control architectures where EMG arbitrates between user and robot [95,97,108,250,253,254]. EMG also serves as an intent gate or dose modulator in hybrid robot+FES systems [4,240,255,256,257,258]. Preprocessing pipelines typically involve bandpass filtering (20–500 Hz), rectification, and moving-average envelope extraction, with electrode placement standardised to target muscles (flexor digitorum superficialis, extensor digitorum communis, biceps brachii) and inter-electrode distance maintained at 20 mm for surface arrays. Key limitations in post-stroke populations—spasticity, co-contraction, fatigue, electrode placement sensitivity, and day-to-day signal non-stationarity—have driven the adoption of multimodal and learning-based architectures rather than EMG-only solutions [77,95,218].

##### Electroencephalographyc Sensors (EEG) and BCIs

EEG-based brain–computer interfaces are motivated by the need to engage patients whose voluntary motor output is too limited for EMG-based control, in alignment with rehabilitation principles emphasising active cortical involvement in neuroplastic recovery [149,239,259]. Motor imagery (MI) EEG—decoding sensorimotor rhythms generated by imagined movements—is the dominant non-invasive BCI paradigm, [260] while steady-state visually evoked potentials (SSVEPs) provide an alternative for discrete command generation [92,261]. Hybrid MI+SSVEP systems use SSVEP for movement selection and MI for go/no-go switching, balancing command richness with user engagement [261,262]. Signal acquisition typically employs 8–64 channel active electrode systems at 250–1000 Hz, with spatial filtering (common spatial patterns, CSP/FBCSP) and temporal feature extraction (band power in mu/beta rhythms, 8–30 Hz) feeding classifiers. CNN-LSTM deep learning pipelines have improved online MI decoding accuracy over classical CSP methods and represent the current state of the art for session- and subject-robust classification [92,196,263,264]. EEG integration is better suited to soft robotic gloves than rigid multi-DOF platforms, as the latter’s mechanical constraints impede the fine motor training that cortical engagement is intended to support [261,262,265,266,267]. Primary barriers remain low signal-to-noise ratio, non-stationarity across sessions, and the calibration burden—particularly challenging in post-stroke populations with altered cortical topography [239,263].

##### Vision-Based Sensors

Vision-based sensing serves three distinct roles: (1) external motion capture for technical validation and kinematic calibration, using optical marker systems or RGB-D cameras (e.g., Microsoft Kinect) for markerless tracking; (2) environmental perception via depth cameras for torso-avoidance and context-aware task-based therapy; and (3) interaction and feedback delivery through virtual reality and mirror-therapy paradigms [5,78,92,177,218,268]. Markerless RGB-D tracking reduces setup complexity relative to optical motion capture and supports home-based monitoring, but remains limited by occlusion and lighting variability [78,177,218,269,270]. Vision systems typically operate at 30–60 fps for joint tracking and 15–30 fps for environmental perception, with latency budgets of less than 50 ms required for closed-loop control applications. The emerging consensus is that vision is most effective as a complement to wearable sensing in a fusion architecture: vision provides global context and drift-free positional constraints, while IMU and EMG provide high-bandwidth local control signals [218].

##### Multimodal Sensor Fusion and Role in Home Deployment

The overarching trend across all sensing modalities is toward multimodal fusion—combining kinematic (IMU, encoders), interaction-force (F/T), and biological intent (EMG, EEG) signals within unified inference frameworks. Fusion architectures range from simple state-machine multiplexing (switching between modalities based on signal quality flags) to learned probabilistic models (Bayesian fusion and deep sensor fusion networks) that weight modality contributions dynamically [92,108,149,237]. Sensor validation in this literature relies primarily on concurrent comparison against laboratory-grade reference systems (optical motion capture and dynamometry) rather than on standardised metrological protocols, which limits cross-study comparability of accuracy claims [271]. For home deployment specifically, sensing choices are constrained by form factor, donning burden, power consumption, and absence of clinical supervision: IMU-based control interfaces and threshold EMG triggering are currently the most feasible modalities for unsupervised home use, while EEG-BCI and high-density force/torque sensing remain primarily laboratory tools pending advances in miniaturisation and calibration automation. Remote sensing data—session logs of movement quality, force profiles, and EMG activation patterns—transmitted via telerehabilitation platforms additionally enables clinicians to monitor home-use adherence and adapt therapy parameters without on-site presence [136,145,147,246,272,273].

#### 4.1.5. Control

The key difference between actuation and helping people move their arms/hands (therapeutic exoskeleton assistance) is how controllers are designed to provide safe and comfortable movement during interaction with humans (pHRI), encourage the person being helped to be actively involved (not passively follow) and accommodate a wide range of different types of impairments and states of the person each day [22,149,218]. In general, most systems use a hierarchical approach where the lowest level (servo loop) provides position/velocity/torque control and the middle level provides a mechanism for the robot to interact safely with the human (via impedance/admittance or force/torque control) and the highest level has a supervisory function related to rehabilitation (e.g., task logic, assistance control, and safety monitoring) [22,149,218]. One common goal of these controllers is to provide the sensation of no resistance (transparency) when the user backdrives the system (to teach or to allow them to perform a specific task) and assistance as needed when they cannot perform the desired task [15,198,250]. These two competing goals are specifically addressed in controllers that utilize model-based feedforward gravity and friction torque compensation to reduce the user’s effort while maintaining a stable interaction [73,159]. For example, the FREE whole-arm exoskeleton uses an upper-level controller running on an industrial PC at 1000 Hz (EtherCAT-based architecture) that implements Zero Moment Control (ZMC) with gravity and friction compensation [274] and provides a backdriveable, transparent behavior that is then enhanced with an assist-as-needed (AAN) layer [250]. Three major concepts have emerged from the control community: (1) safe and compliant interaction through impedance/admittance (with many examples that enhance transparency), (2) individualised assistance using assist-as-needed and performance adaptations, and (3) supervision based on the user’s intended actions determined through EMG/EEG, and/or interaction sensors—often integrated into closed-loop (“patient-in-the-loop”) systems with explicit safety and progression rules [92,149,168,198,218].

##### PID Control

PID and PD controllers are the most widely implemented low-level method, valued for simplicity, low computational cost, and ease of integration in embedded motor drives [77,165,203]. Their primary role is inner-loop trajectory tracking during passive and therapist-programmed exercises. Key limitations in post-stroke use are well documented: pure position tracking generates high corrective torques (and high cable tensions in tendon-driven systems) when spasticity prevents the limb from following the reference trajectory, risking patient discomfort or injury [99,154,203,275]. This makes PID unsuitable as a standalone controller for interactive rehabilitation; it is instead used as the inner loop of a more compliant outer-loop strategy (impedance/admittance), where interaction forces modify the reference before it reaches the PID [73,93,96,197]. In cable-driven systems, PID must be reformulated in the cable-length domain rather than joint-space, with explicit cable-slack detection to prevent tracking degradation [73,79,226,252,276]. A recent trend is augmenting PID with learning-based torque prediction (LSTM, BLSTM, and GRU trained on EMG) to improve disturbance rejection and user adaptation while retaining the dependability of classical feedback [96,97,108,237,277,278].

##### Admittance and Impedance Control

Admittance and impedance control are the dominant mid-level interaction strategies, directly addressing the spasticity and involuntary resistance characteristic of post-stroke populations [149,198,218]. Admittance control—preferred when the hardware is position-controlled—measures interaction forces at the human–robot interface and modifies the commanded reference accordingly, allowing the device to yield rather than fight unexpected patient resistance [73,93,198]. Impedance control renders a target mechanical impedance (virtual spring–damper–mass) between device motion and interaction force, and is preferred when the hardware provides compliant actuation (SEA, hydraulic elastic drives) [149,217,279]. Both strategies share a critical clinical parameter: the impedance gains. Lower impedance prioritises safety and comfort (accepting trajectory deviations), while higher impedance improves kinematic fidelity—a trade-off that should be adjusted to the patient’s spasticity level and recovery stage [77,100,103,218,275,280]. Model-based gravity and friction compensation is a near-universal prerequisite to prevent the robot’s own dynamics from inflating the apparent impedance and degrading transparency [155,217,281]. Residual transmission non-idealities (cable friction and Bowden lag) complicate impedance rendering and must be addressed either in the mechanical design or through robust control formulations [31,73,78,181,202,218].

##### Model Predictive Control (MPC)

MPC has been explored as a high-level optimisation layer for impedance scheduling and assist-as-needed (AAN) regulation, formulating assistance modulation as a finite-horizon optimisation problem that balances task completion with active patient effort [2,5]. Its application in wearable upper-limb exoskeletons remains limited by the computational cost of real-time optimisation, the accuracy requirements of the human-exoskeleton model, and latency constraints; MPC is therefore typically employed above a conventional low-level torque/force loop rather than as a direct actuator controller [5,164,241].

##### Adaptive Control

Adaptive control frameworks—often termed patient-in-the-loop or assist-as-needed—represent the current paradigm for personalised rehabilitation, continuously adjusting assistance and resistance based on real-time estimates of patient performance, effort, and intent [149,218,221,282]. The adaptation architecture typically operates at three coupled layers: (1) gravity/friction compensation for intrinsic transparency; (2) AAN or resistance shaping driven by tracking error and performance metrics; and (3) intent/effort estimation via EMG or interaction forces to reduce dependence on pre-specified models [77,218]. Representative implementations (e.g., CASIA-EXO and FREE/ZMC) demonstrate mode-switching logic that provides resistance when performance is high, assistance when error grows, and safety stops when error exceeds defined bounds [123,283,284]. Key open challenges are stability under time-varying human dynamics, EMG reliability in spastic populations, and the need to validate adaptive laws against clinically relevant endpoints rather than purely kinematic tracking error—to avoid policies that inadvertently promote compensatory rather than recovery-oriented movement [5,149,218,285,286].

##### Reinforcement Learning Control

RL is an emerging but under-represented paradigm in upper-limb exoskeletons, with most explicit RL implementations currently in lower-limb and gait-assist devices [287,288,289,290,291,292,293]. Its appeal for post-stroke rehabilitation lies in framing assistance personalisation as a sequential decision-making problem that can learn patient-specific optimal policies under uncertainty and variability. The most viable near-term applications for upper-limb systems are high-level policy learning for AAN gain scheduling and compliance personalisation, operating above a certified low-level stability controller rather than directly commanding joint torques [256,294]. Barriers to clinical translation include safe exploration near vulnerable upper-limb joints, difficulty defining rewards that reflect neurorehabilitation quality rather than tracking performance, and data-efficiency constraints in clinical environments. Simulation-based pre-training, conservative online updates, and embedding RL within patient-in-the-loop adaptive frameworks are the strategies most consistent with near-term clinical feasibility [256,294].

### 4.2. Clinical and Biomedical Significance

The clinical evidence synthesised across the RCT and clinical-trial subsets (58 of 219 included studies)—with observational studies and case reports cited only for contextual illustration—converges on two broad findings, reported here as drawn from the conclusions of the included primary studies and not from independent meta-analytic pooling. First, at the impairment level, available evidence suggests potential benefits: the majority of RCTs and clinical trials reporting FMA-UE and MAS outcomes describe improvements following exoskeleton-assisted therapy, with the most consistent gains observed at proximal joints (shoulder and elbow). However, certainty remains limited by heterogeneity, small samples, blinding limitations, inconsistent dosing, and limited long-term follow-up, even in the subset of chronic-stage RCTs reporting statistically significant FMA-UE and BBT advantages over conventional therapy. Second, at the activity and participation levels, reported improvements in ARAT, BBT, and patient-reported outcomes (MAL) are less consistent and more heterogeneous across devices, protocols, and populations. Transfer of impairment-level gains to sustained improvements in ADLs and real-world arm use is insufficiently documented in the current evidence base, reflecting both the limited follow-up durations of most trials and the relative insensitivity of participation-level instruments to isolated upper-limb changes over short intervention windows. For non-randomised and observational studies, common shortcomings—absence of prospective sample size calculation, lack of an unbiased endpoint assessment, and inadequate follow-up—further limit the strength of inferences that can be drawn; these shortcomings are noted narratively rather than through a formal MINORS scoring, consistent with the review’s narrative (non-systematic) design. Across all study designs, the heterogeneity in device architecture (rigid vs. soft, proximal vs. distal, and motor-driven vs. pneumatic), intervention dosing (session frequency, duration, and total repetitions), and outcome selection complicates direct cross-study comparison. The thematic subsections below synthesise the evidence within each clinical domain, maintaining throughout the distinction between clinical studies involving stroke patients, technical validation studies with healthy subjects, and engineering prototype studies—a distinction essential for correctly attributing the strength of any clinical claim.

#### 4.2.1. Selection of the Clinical Outcomes

Outcome selection in post-stroke exoskeleton trials requires alignment between the device’s mechanical target (distal hand, wrist, and proximal arm), the therapy’s goal (impairment reduction, functional recovery, or real-world use), and the scale’s measurement level (body structure/function, activity, and participation). Ethical and resource considerations reinforce the importance of choosing outcomes with established psychometric properties and clinically meaningful thresholds, to ensure that a positive effect is scientifically detectable and interpretable. Ten standardised instruments are represented across the reviewed literature; they form a complementary three-level framework rather than interchangeable alternatives [29,295,296,297,298,299,300,301,302].

##### Impairment Level

The Fugl-Meyer Assessment—Upper Extremity (FMA-UE; 0–66, higher = better) is the most widely used impairment endpoint, assessing synergistic and selective motor control across 33 items [136,262,264,303,304]. A critical reporting practice is decomposing the total score into proximal (42 points) and distal (24 points) subscores: shoulder–elbow devices should report proximal changes; hand/glove devices should report distal changes, to avoid diluting device-specific effects in the total score [12,77,216]. Statistically significant FMA-UE changes do not always meet the minimal clinically important difference (MCID); reporting MDC/MCID-based responder rates and baseline severity stratification (mild/moderate/severe) are recommended practice [3,31,77,216,305]. The Modified Ashworth Scale (MAS; 0–4) is the standard spasticity metric, assessing velocity-dependent resistance during passive stretch at the major upper-limb joints. Its known reliability limitations (assessor dependence and ordinal resolution) make it insufficient as a sole spasticity endpoint; paired kinematic descriptors from embedded exoskeleton sensors (passive stretch velocity, final angle) offer a more objective and continuous alternative [12,31,77]. The NIH Stroke Scale (NIHSS), though a global neurological deficit measure, serves as a baseline characterisation and covariate variable in exoskeleton studies rather than a primary outcome—it is relatively insensitive to the distal motor improvements that exoskeleton therapy targets [77,214,306,307].

##### Activity Level

The Action Research Arm Test (ARAT; 0–57) is the primary activity-level endpoint for upper-limb and hand exoskeleton trials, assessing grasp, grip, pinch, and gross movement across 19 tasks (MDC = 3.5 points; MCID = 12–17 points) [31,77,216]. Subscale reporting is recommended to identify which movement categories are most affected by the device. The Box and Block Test (BBT; blocks/60 s) provides a rapid, reliable measure of gross manual dexterity (inter-rater reliability = 0.98), sensitive to changes in grasp–release capability [31,216,269,303,308,309]. A key distinction for exoskeleton research is device-on versus device-off performance—the former reflects immediate functional compensation, the latter sustained motor recovery—and these should not be conflated in reporting (MCID = 6 blocks) [3,145,186]. The Wolf Motor Function Test (WMFT; 0–75) complements ARAT by capturing timed, goal-oriented functional performance and is commonly paired with FMA-UE and ARAT to triangulate across impairment, activity, and performance dimensions [77,310,311]. The Nine-Hole Peg Test (9HPT; completion time) adds fine dexterity assessment for hand-focused devices, specifically targeting finger individuation and coordination not fully captured by gross dexterity measures [77,147,230].

##### Participation and Quality of Life Level

The Barthel Index (BI/MBI; 0–100) and Functional Independence Measure (FIM) are global ADL and independence measures whose primary role in exoskeleton trials is patient stratification at baseline and secondary detection of functional generalisation beyond the trained limb. Both instruments are relatively insensitive to isolated upper-limb improvements, particularly over short intervention periods, as compensatory strategies or co-existing limitations (gait, cognition) can mask arm-specific gains [77,121]. The Motor Activity Log (MAL; 0–5 on How Much and How Well scales across 30 daily activities) is the standard patient-reported outcome for real-world paretic limb use, providing ecological validity that performance-based lab tests lack. Recall and expectation biases inherent to self-report require paired objective measures and follow-up assessments to distinguish sustained recovery from transient post-treatment enthusiasm [77,272,310,311].

##### Recommendation for Outcome Battery Design

The consistent finding across the reviewed literature is that no single instrument captures the full recovery trajectory from impairment to real-world use. Best practice is a multi-level battery: FMA-UE (proximal or distal subscale) for impairment; ARAT and/or BBT for activity; MAL for participation; MAS as a baseline and monitoring descriptor; and NIHSS as a cohort characterisation covariate. Reporting should include MDC/MCID-referenced responder rates, baseline severity classification, and clear differentiation of device-on from device-off assessments [77,136,145,218,312].

#### 4.2.2. Stages of the Pathology

The post-stroke rehabilitation trajectory is conventionally divided into acute (<1 month), subacute (1–6 months), and chronic (>6 months) stages, each presenting distinct neurobiological mechanisms, clinical constraints, and device requirements.

##### Acute Stage

The acute stage represents a window of heightened neuroplasticity when spontaneous recovery is maximal and high-dose, task-oriented practice can yield disproportionate benefit if delivered safely [77,238,313]. Exoskeletons are attractive in this phase because they can bridge the gap between voluntary motor intent and functional execution in patients with minimal voluntary activation, supporting early exposure to reach-to-grasp kinematics [13,149,248,255,314]. Practical constraints include medical instability, fluctuating tone, fatigue, and attention deficits, which require conservative safety envelopes, rapid donning/doffing, and therapist-compatible workflows [77,315]. Acute-stage study designs face a distinctive signal-to-noise problem: spontaneous neurological recovery can mask or mimic device-specific effects, requiring dose-matched control conditions and impairment-stratified randomisation to isolate intervention benefit [13,31,77].

##### Subacute Stage

The subacute stage—where spontaneous biological recovery and structured rehabilitation overlap—is the most represented in the current evidence base and is considered the prime window for motor restitution [77,238,316]. Distal dysfunction (wrist–finger extension and flexor synergy) is the dominant target, making glove-type exoskeletons and wrist–finger modules particularly relevant [31,77,238]. Wearable soft exoskeletons and BCI-driven assistive gloves have shown clinically significant FMA-UE and ARAT improvements, with intent-driven assistance supporting active patient engagement aligned with neuroplasticity principles [212,240,264]. Key design requirements are high training dose, consistent kinematic guidance, and flexible integration with concurrent inpatient/outpatient rehabilitation programs [12,77,144,206].

##### Chronic Stage

The chronic stage (>6 months) shifts emphasis from motor restitution toward sustained use, home deployment, and functional independence for patients with persistent upper-limb disability [77,238,317]. The rehabilitative versus assistive distinction is clinically important: rehabilitative devices target training-induced recovery via repetitive volitional practice; assistive devices provide daily functional support during ADLs [145,147,318]. Soft robotic gloves and cable-driven wearable systems are dominant due to their portability, compliance, and low setup burden [77,145,317]. Volitional intent decoding via EMG thresholds and hybrid robot+FES systems are commonly used to promote active participation in populations with moderate-to-severe residual weakness [196,216,240,319,320]. A persistent gap is the exclusion of the most severely impaired chronic patients from trials [321]—precisely the individuals with the highest unmet need [77,186,310,311,317].

#### 4.2.3. Experimental Setting

The choice of experimental setting determines the available supervision level, instrumentation density, dose delivery model, and ecological validity of the study, and directly shapes device design requirements.

##### Laboratory Setting

Laboratory settings provide the highest degree of experimental control—standardized positioning, rich sensor suites (EMG, motion capture, force/torque), scripted dosing protocols, and supervised safety monitoring—making them the primary environment for technical validation, algorithm development, and early-phase efficacy signal detection [162,218]. Their primary limitation is limited ecological validity: controlled workspaces and structured task paradigms may not capture the variability of real-world use [77,121,218].

##### Inpatient Setting

Inpatient rehabilitation settings are the dominant clinical deployment context, where exoskeletons are used as adjuncts to standard occupational/physical therapy rather than replacements [31,216,317]. Key constraints are the competition for time within densely scheduled rehabilitation days, progressive fatigue from cumulative therapies, and the cognitive/perceptual deficits common post-stroke that require staged supervision models and usability-optimised device interfaces [160,186,322].

##### Outpatient Setting

Outpatient clinics serve as the transition point between supervised inpatient care and independent home use, combining scheduled therapist-supervised fitting and safety screening with progressive increases in patient autonomy and repetition volume [33,238,246]. Rapid donning/doffing is a critical practical requirement, as excessive setup time offsets the gains from increased training intensity within limited appointment durations [246,304,323]. Most outpatient protocols emphasize functionally oriented task practice (grasp–release of everyday objects) rather than isolated joint cycling [324], with the occupational therapist monitoring and correcting compensatory movement patterns [33,212,304,325].

##### Home Setting

Home settings are increasingly targeted to increase therapy dose, reduce access barriers, and extend recovery continuity beyond scheduled clinical contact [126,136,145,147]. Device requirements in this context are more demanding: low distal mass, passive safety features (mechanical stops and conservative force/torque limits), simplified user interfaces, and battery-powered portability—favouring electric motor and cable-driven architectures over pneumatic or hydraulic systems [31,78,145,202,218]. Remote supervisory capability (session logging, adherence tracking, and performance summaries) is a near-essential complement to home deployment, enabling clinicians to guide progression and detect maladaptive compensation patterns without continuous on-site presence [136,145,147,246,272,273].

#### 4.2.4. Experimental Studies

The evidence base for upper-limb and hand exoskeletons in post-stroke rehabilitation has been built progressively through a hierarchy of study designs, each serving distinct epistemic goals—from early hardware validation to confirmatory efficacy trials.

##### Experimental Protocol

Participant selection and intervention protocol design in exoskeleton studies serve three goals: assembling a clinically homogeneous cohort, ensuring safe device interaction, and delivering a structured high-dose programme that integrates with existing rehabilitation [186,238,315,317]. Standard inclusion criteria across the reviewed literature specify time post-stroke (subacute vs. chronic), baseline motor impairment (typically defined by FMA-UE score ranges and MAS grading), cognitive and postural prerequisites (preserved comprehension, ability to remain seated for ≥30 min), and medical stability [186,264,315,326]. Common exclusions are severe spasticity/contractures, skin integrity issues, and significant cognitive/communication deficits that would prevent safe device interaction [136,186,315]. Intervention protocols typically prescribe 3–5 sessions per week over 4–8 weeks, with an initial therapist-led parameter adjustment session to individualise task and loading parameters (target distance, object weight/geometry, shoulder abduction load) to each participant’s active capacity [103,186,264,326]. Best practice in recent protocols is to quantify achieved dose—through device-logged repetition counts and time-on-task—alongside planned dose, to assess intervention fidelity and support scalability modelling [84,111,136]. Secondary endpoint batteries commonly combine impairment (FMA-UE and MAS) and activity (ARAT and BBT) measures with quality-of-life (EQ-5D-5L and SS-QOL) and device usability/acceptability instruments (SUS and QUEST 2.0), evaluated at baseline, post-treatment, and follow-up [136,186,264,326].

##### Case Studies

Case studies (single-patient reports and small series) are the standard first translational step, enabling rapid iteration of hardware fit, session structure, and safety procedures before larger trials [136,164,327]. Their four pragmatic goals are: (i) safety screening (skin integrity, fatigue, adverse events); (ii) usability and acceptance (donning/doffing, comfort, perceived usefulness); (iii) protocol feasibility (session length, progression rules, therapist workload); and (iv) signal-seeking efficacy for a specific impairment phenotype [124,145,246,323]. Granular safety reporting is particularly important: transient pressure marks or redness that resolve after device removal are distinct from adverse events but may affect long-term adoption and should be documented [136,323,327,328]. Statistical inference is not meaningful at these sample sizes, but case studies define the boundary conditions—fit, tolerance, and usability—within which larger studies can operate [119,164,323,329].

##### Observational Studies

The observational tier complements randomised trials by characterising real-world usability, dose delivery, and short-term functional change in heterogeneous post-stroke populations [121,164]. Three types are represented [121,137]: (1) cross-sectional device-on/off comparisons, which isolate immediate functional assistance from sustained recovery using within-subject designs that control for lesion and chronicity variability [312,330]; (2) prospective feasibility cohorts with pre-post assessment of safety, adherence, and impairment/activity outcomes [331,332,333]; and (3) user-centred development studies using iterative structured feedback from stroke survivors and occupational therapists to refine hardware and interface design [137,138,247]. Common outcome assignments across observational designs are: ARAT/BBT for activity-level cross-sectional comparisons, FMA-UE for longitudinal impairment tracking [193,334,335], and SUS/QUEST for usability and satisfaction as primary endpoints in human factors studies [121,137,138,216,312,332]. Predictable limitations include selection bias toward motivated/tolerant participants, baseline heterogeneity, absence of follow-up, and outcome–mechanism mismatch when recovery-oriented scales are applied to primarily assistive devices [121,164]. Recommendations from the literature to maximise the informativeness of observational studies for subsequent RCT design are: standardise feasibility endpoints and safety reporting; correlate device-logged dose with clinical outcomes; and embed structured user-centred requirements early, since interface resistance is among the largest determinants of adherence and home translation [121,137,138].

##### Pilot and Feasibility Studies

Pilot and feasibility studies are the essential bridge between basic science and controlled clinical trials, with three primary goals: assessing risk and tolerability (skin irritation, spasticity exacerbation, and fatigue); validating intervention delivery (session frequency, duration, and protocol adherence); and generating outcome variance estimates to power adequately designed confirmatory trials [136,272,322]. The distinction between pilot and feasibility is methodologically important: pilot studies focus on safety and preliminary efficacy signals when prior evidence is insufficient for sample size calculations; feasibility studies focus on practical trialability—recruitment, retention, adherence, and implementation barriers—rather than definitive efficacy [164,336]. A consistent finding is that feasibility is limited not only by device performance but by participant heterogeneity and outcome sensitivity [337,338,339,340,341], motivating exploratory stratification (by impairment severity, chronicity, or residual voluntary control) to identify subgroups likely to respond in subsequent larger trials [255,322,342].

##### Clinical Trials and Randomized Clinical Trials (RCTs)

Clinical trial evidence has progressively shifted from feasibility testing toward confirmatory efficacy evaluation using randomised controlled designs [3,33,44,186,206]. Three RCT architectures are represented: comparative trials (exoskeleton-assisted vs conventional therapy); controlled adjunct trials (exoskeleton added to standard care vs standard care alone); and technology-contrast trials (comparing different robotic paradigms) [1,21,33,310]. Chronic-stage RCTs with structured multi-week protocols—typically ≥3 sessions/week for 8 weeks totalling ≥24 sessions—have demonstrated statistically significant FMA-UE and BBT advantages over conventional therapy, with no serious adverse events reported across multiple trials [33,310,343,344,345,346]. Subacute RCTs are an emerging design space, with trials examining not only whether exoskeleton training works but how it should be organised (e.g., unilateral vs bilateral delivery) [347,348] and incorporating mechanistic correlates such as quantitative EEG indices alongside clinical outcomes [1,229,264,315]. Persistent methodological limitations across the RCT literature are: participant heterogeneity (stage and severity), small per-device sample sizes, inconsistent inclusion of participation-level outcomes (MAL, ABILHAND), and limited follow-up durations [21,44,310]. A positive trend is the progressive improvement in methodological rigour: recent designs incorporate double-blinding, stratified randomisation, allocation concealment, pre-specified primary outcomes referenced against MDC/MCID thresholds, and extended multi-timepoint follow-up—moving toward the standard required for device-specific clinical recommendation [44,186,315,326].

##### Review Studies

The review literature has matured through three generations: early technology-mapping surveys cataloguing device architectures and engineering challenges; hand-specific syntheses comparing device families (exoskeleton vs end-effector); and systematic reviews and meta-analyses evaluating clinical effectiveness [22,149,349]. Key conclusions from this body of work are [149,349,350,351,352,353,354,355,356,357,358,359]: (1) the field has transitioned from technology-push to clinically-framed synthesis, with design reviews and clinical meta-analyses increasingly coexisting [22,231,350]; (2) hand exoskeletons are an increasingly distinct sub-domain, with comparative effectiveness syntheses specifically addressing finger–hand recovery separate from proximal arm rehabilitation [121,181,360]; and (3) device-specific recommendations remain premature because most systematic reviews aggregate heterogeneous devices and protocols [361,362]—future reviews should stratify by device architecture (rigid/soft), target joints (proximal/distal), training intensity, and assistance strategy to support actionable clinical inference [349,350].

#### 4.2.5. Limitations and Future Directions

The present narrative review is subject to several limitations that must be considered when interpreting its findings.

Regarding the review methodology: the formal, PRISMA-tracked search was conducted in PubMed and Scopus, limited to English-language peer-reviewed publications; supplementary exploratory searches directly in IEEE Xplore, Web of Science, Embase, and ACM Digital Library confirmed that Scopus’s indexing coverage adequately captured records from these sources. Grey literature, preprints, conference proceedings, and clinical trial registries were deliberately excluded, in line with the narrative rather than systematic nature of this review, which prioritises peer-reviewed, citable evidence over exhaustive retrieval of unpublished or ongoing studies. This approach may still have introduced publication and language bias, potentially omitting relevant studies published in other languages or available only as grey literature. The review protocol was not prospectively registered, and no formal protocol document was prepared or published prior to the review; this is acknowledged as a transparency limitation, though no material deviations from the initial research plan occurred. As a literature synthesis study, formal PROSPERO registration was not applicable. The volume and heterogeneity of the included studies (*n* = 219, spanning engineering prototypes to multi-site RCTs) precluded quantitative meta-analysis, formal funnel plot assessment of publication bias, or certainty-of-evidence grading using the GRADE framework; these remain important unmet methodological needs for the field.

Regarding the evidence base itself: heterogeneity in study design—ranging from single-patient case studies and bench-level engineering reports to randomised controlled trials—means that studies serve fundamentally different epistemic functions and cannot be compared as equivalent evidence. The three-category classification (clinical studies with stroke patients; technical/laboratory studies with healthy subjects; engineering prototype studies) maintained throughout this review is intended to make this distinction explicit, but the nature of a narrative synthesis is that it integrates across these categories descriptively; Table 1 reports the study-type stratification adopted to prevent conflation of clinical and technical evidence.

The majority of clinical studies are underpowered, of insufficient duration, and lack long-term follow-up, limiting the ability to evaluate the sustainability of recovery trajectories and the degree to which exoskeleton use promotes community participation. The predominance of impairment and activity metrics (FMA-UE, ARAT, and BBT), with relatively limited use of participation-level and patient-reported outcomes (MAL and ABILHAND), means that functional generalisation beyond the trained task remains difficult to establish from the current evidence. Cross-sectional and early observational designs—while valuable for demonstrating assist-as-needed effects, usability, and safety—do not directly measure unassisted recovery, long-term participation gains, or real-world functional transfer [363,364,365,366,367].

The explicit-evidence labelling rule (Section 2.4) may have systematically under-reported technical implementation details that are less consistently described in abstracts and methods sections than device-level structural features, a directional bias that should be considered when interpreting the relative frequency of sensor-related labels.

Non-clinical barriers further impede adoption and scaling. These include device weight and bulkiness, ergonomic and donning/doffing difficulties, limitations in real-time control adaptability, variable user and therapist acceptance, and cost and accessibility constraints. These factors collectively contribute to the persistent gap between promising laboratory and clinic-based performance and successful real-world deployment.

Inter-rater agreement for screening, data extraction, and label assignment was not formally quantified (e.g., Cohen’s kappa); while discrepancies were resolved through consensus and third-reviewer adjudication, the absence of a quantified agreement statistic limits independent assessment of labelling reliability and is noted here as a transparency limitation alongside the absence of prospective registration.

Future research priorities arising from these limitations are the following:

1. *Well-powered, adequately controlled RCTs.* Multicentre randomised trials with dose-matched control conditions, intervention durations exceeding five weeks, and follow-up periods of at least three to six months are needed to differentiate device-specific effects from practice and learning effects, and to establish whether gains persist and generalise outside supervised settings.
2. *Standardised outcome reporting aligned to device target and therapy goal.* Future studies should routinely report FMA-UE proximal and distal subscores separately, include at least one ADL-oriented patient-reported outcome (MAL, ABILHAND), and specify baseline severity classification and MCID/MDC-referenced responder rates to enable meaningful cross-study comparability.
3. *Stratified analyses by impairment phenotype.* Given the heterogeneity of post-stroke upper-limb deficits, stratified analyses by spasticity level, chronicity, and residual voluntary control are essential to identify which patient subgroups benefit from which device architectures and assistance strategies, and to prevent averaging-out of benefits in heterogeneous cohorts.
4. *Sensor-driven dose quantification and home-use monitoring.* Trials should systematically log device-recorded dose metrics (repetition counts, time-on-task, and movement quality indices from embedded IMU and force sensors) alongside planned dose, and integrate remote supervisory capability—session logs, EMG activation patterns, kinematic performance summaries—to enable clinician-guided progression and detection of maladaptive compensation without continuous on-site presence.
5. *Clinical evaluation of long-term adherence and cost effectiveness for home-based programmes.* As exoskeleton systems increasingly target home and community settings, long-term adherence, device durability under real-world use, and cost-effectiveness relative to standard care must be integrated as primary endpoints rather than secondary observations [368]. These data are prerequisites for health technology assessment and reimbursement consideration.

## 5. Conclusions

This narrative review synthesised 219 studies on upper-limb and hand exoskeletons for post-stroke rehabilitation published between 2010 and 2025, integrating evidence from primary clinical studies, technical engineering studies, and previous systematic reviews within a unified label-based framework. The added value of this approach lies in connecting engineering descriptors (anatomical target, structure, actuation, sensing, control, and AI/telerehabilitation features) with biomedical descriptors (stroke stage, setting, study design, and outcome domain) within a single cross-disciplinary synthesis designed for both technical and clinical audiences. Overall, the field is moving from rigid, laboratory-centred systems toward lighter, cable-driven, soft, glove-based, and increasingly home-oriented solutions, driven by advances in sensing—particularly multimodal fusion of IMU, EMG, EEG, and force/torque modalities—and by AI-enabled adaptive control and telerehabilitation platforms. At the clinical level, the available evidence suggests potential impairment-level benefits, especially for proximal motor control and reaching performance, but certainty remains limited due to heterogeneity, small samples, blinding limitations, inconsistent dosing, and limited long-term follow-up; gains in hand and finger dexterity are smaller and even more heterogeneous across devices and protocols. Transfer to ADLs and real-world function remains inconsistently documented and is insufficient to support firm conclusions about functional independence.

Future work should prioritise adequately powered multicentre trial designs, standardised outcome reporting with proximal/distal FMA-UE subscores and ADL-oriented endpoints, stratified analyses by impairment phenotype, sensor-driven dose quantification, and rigorous evaluation of long-term home-use adherence and cost-effectiveness. The transition from laboratory efficacy to real-world clinical impact will require not only continued engineering innovation in sensing and control but also the methodological infrastructure—registered protocols, standardised reporting frameworks, and sustained follow-up—to demonstrate that these innovations translate into meaningful and durable improvements in the lives of stroke survivors.

## Figures and Tables

**Table 1:** Stratification of the 219 included studies by study type, with the evidential role attributed to each category in this review. This is a design-based stratification, not a formal risk-of-bias instrument (RoB 2/MINORS); it is intended to make explicit which subset of studies supports the clinical statements in Section 4.2.

Study Type	N (of 219) ^1^	Role in This Review	Design-Based Caveat
RCT	16 (7%)	Basis for impairment/activity-level clinical claims	Randomised, but therapist/patient blinding rarely feasible in rehab robotics
Clinical trial (non-randomised/uncontrolled)	42 (19%)	Supporting clinical claims, lower certainty	No randomisation; control-group quality varies
Observational	16 (7%)	Descriptive clinical context only	No control group; confounding not addressed
Case study/case series	12 (5%)	Illustrative only	n=1–few; not generalisable
Protocol paper	12 (5%)	Methods description only	No outcome data; excluded from all efficacy claims
Review/meta-analysis	22 (10%)	Background citation only	Not primary evidence; excluded from synthesis counts
Technical/engineering study	68 (31%)	Device/technology trend evidence	Not clinical evidence
Pilot/feasibility study	47 (21%)	Feasibility/safety, hypothesis-generating	Not powered for efficacy

^1^ Percentages sum to more than 100% because a single study can carry more than one study-type label (e.g., a pilot RCT).

**Table 2:** Sensor centered synthesis by modality, summarising placement, acquisition parameters, calibration, fusion strategy, control-loop and clinical-assessment roles, and home-deployment suitability, as reported across the included literature (Section 4.1.4). Parameters reflect typical ranges reported across studies rather than per-study extracted values.

Modality	Placement	Sampling/Hardware	Calibration	Fusion/Processing	Role in Control Loop	Role in Clinical Assessment	Home-Use Suitability
IMU	Limb segments, joints	100–400 Hz; MEMS accelerometer/gyro/magnetometer	Bias correction, gravity alignment, axis registration	Madgwick/Mahony/EKF fusion	Joint-angle feedback, contralateral mirroring	Movement quality/dose logging	High—low cost, low burden
Force/torque	Cuff, handle, joint, phalanges	Multi-axis wrench sensors, joint-level torque sensors, load cells	Zeroing, temperature drift correction	Direct joint-torque preferred over current-based estimation	Impedance/admittance, assist-as-needed	Interaction-force dosage monitoring	Emerging—miniaturised strain-gauge/capacitive cells
EMG	Target muscles (FDS, EDC, biceps brachii), 20 mm inter-electrode	Bandpass 20–500 Hz	Electrode placement standardisation	Rectification + moving-average envelope; CNN/LSTM pattern recognition	Threshold/proportional/ pattern-recognition control	Voluntary engagement indicator	Moderate—sensitive to spasticity, day-to-day drift
EEG-BCI	Scalp, 8–64 channels	250–1000 Hz	Session-specific recalibration	CSP/FBCSP spatial filtering, CNN-LSTM decoding	MI/SSVEP-based command generation	Cortical engagement marker	Low—high SNR/ calibration burden
Vision-based	External (motion capture) or wearable camera	30–60 fps (joint tracking), 15–30 fps (environment); latency <50 ms	Marker-based or markerless registration	Complements IMU/EMG with global, drift-free position reference	Environmental perception, VR/mirror feedback	Kinematic validation, occlusion-robust outcome capture	Moderate—occlusion/ lighting sensitive

## Data Availability

The template data collection forms, and the full dataset extracted from the included studies, will be submitted to the Data journal as associated with this article. Additional data or materials used in the review are available from the corresponding author upon reasonable request.

## Supplementary Materials

The following supporting information can be downloaded at: https://www.mdpi.com/article/10.3390/s26175373/s1, Table S1: Search strings; Table S2: References and associated labels; Glossary: Label Taxonomy Glossary.
