# Supplementary material for: Recent Advances in Sensor-Based Upper-Limb and Hand Exoskeletons for Post-Stroke Rehabilitation: A Technical and Biomedical Review
Source: Sensors (Basel). 2026 Aug 25;26(17):5373. doi: 10.3390/s26175373 (PMC13568222; doi:10.3390/s26175373)
Supplement: Supplementary file 1 [file sensors-26-05373-s001.zip › sensors-4367847-supplementary.pdf]

Review

Recent Advances in Sensor-Based Upper-Limb and Hand Exoskeletons for Post-Stroke Rehabilitation: A Technical and Biomedical Review

Alberto Borboni [1,*], Matteo Verzeletti [1], Alireza Rastegarpanah [2] and Jorge Hugo Villafañe [3]

1 Department of Mechanical and Industrial Engineering, Università degli Studi di Brescia, 25123 Brescia, Italy

2 School of Engineering and Physical Sciences, Aston University, Birmingham B4 7ET, UK; a.rastegarpanah@aston.ac.uk

3 Casa de Villafañe APS-Science, Culture & Education, 10045 Piossasco, Italy; mail@villafane.it

* Correspondence: alberto.borboni@unibs.it; Tel.: +39-030-3715401

Supplmentary materials

Table S1. Search strings.

Database and Search String
**Scopus Search String**
`TITLE-ABS-KEY(("upper limb*" OR "upper-limb*" OR upper W/1 limb OR "upper extremit*" OR "upper-extremit*" OR upper W/1 extremit* OR arm OR arms OR forearm* OR hand OR hands OR wrist* OR finger*) AND (exoskeleton* OR exosuit* OR "powered exoskeleton*" OR "wearable robot*" OR "wearable robotic*" OR "robotic glove*" OR "soft glove*" OR "glove-based" OR ("hand orthos*" OR "wrist orthos*" OR orthose* OR orthosis*) W/3 (robotic* OR powered)) AND (stroke OR "post-stroke" OR poststroke OR "cerebrovascular accident*" OR CVA OR hemipleg* OR hemipare* OR "brain infarct*" OR "ischemic stroke" OR "intracerebral hemorrhag*" OR "cerebral hemorrhag*") AND (rehabilitat* OR therap* OR training OR neurorehabilitat* OR physiotherap* OR "occupational therap*" OR telerehab* OR "home-based" OR "home care")) AND (PUBYEAR > 2009 AND PUBYEAR < 2026)`
**PubMed Search String**
`("Upper Extremity"[Mesh] OR "upper limb*"[Title/Abstract] OR "arm"[Title/Abstract] OR "hand"[Title/Abstract] OR "wrist"[Title/Abstract] OR "finger*"[Title/Abstract]) AND ("Exoskeleton Device"[Mesh] OR "exoskeleton*"[Title/Abstract] OR "exosuit*"[Title/Abstract] OR "wearable robot*"[Title/Abstract] OR "robotic glove*"[Title/Abstract] OR "orthosis"[Title/Abstract]) AND ("Stroke"[Mesh] OR "stroke"[Title/Abstract] OR "post-stroke"[Title/Abstract] OR "cerebrovascular accident"[Title/Abstract] OR "hemiplegia"[Title/Abstract] OR "hemiparesis"[Title/Abstract]) AND ("Rehabilitation"[Mesh] OR "rehabilitation"[Title/Abstract] OR "therapy"[Title/Abstract] OR "training"[Title/Abstract] OR "telerehabilitation"[Title/Abstract]) AND ("2010/01/01"[Date - Publication] : "2025/11/10"[Date - Publication])`

Table S2. References and associated labels

Title	Year	Labels
Recovr Glove: Accessible Hand Exoskeleton for Stroke Rehabilitation and Everyday Aid [1]	2025	sensors, actuators, control, home_telerehab, hand, finger, glove, soft-exosuit, study-technical, EMG-interface, setting-lab, actuation-cabled, actuation-motorized

Title	Year	Labels
Development and Validation of Customizable, Adaptive SPring-Actuated Hand EXoskeleton (CASPHEX) Designed for Home-Based Training of Finger Movements Post-Stroke [2]	2025	sensors, actuators, control, AI, home_telerehab, hand, wrist, finger, multi-joint, rigid-exoskeleton, study-pilot-feasibility, study-protocol, study-review, study-technical, EMG-interface, force-torque-sensing, setting-lab, outcome-BBT, stage-chronic, actuation-cabled, actuation-motorized, AI-intent-decoding
Feasibility of post-stroke hand rehabilitation supported by a soft robotic hand orthosis in-clinic and at-home [3]	2025	actuators, control, AI, home_telerehab, hand, finger, elbow, shoulder, arm-general, glove, soft-exosuit, study-pilot-feasibility, study-case, study-protocol, study-review, setting-outpatient, setting-home, outcome-FMA-UE, outcome-ARAT, outcome-MAS, stage-chronic, actuation-motorized, AI-intent-decoding
Effects and neural mechanisms of a brain?computer interface-controlled soft robotic glove on upper limb function in patients with subacute stroke: a randomized controlled fNIRS study [4]	2025	hand, wrist, finger, glove, soft-exosuit, study-RCT, study-clinical-trial, study-protocol, EEG-BCI, setting-inpatient, outcome-FMA-UE, outcome-ARAT, outcome-Barthel, outcome-MAS, stage-subacute, actuation-motorized
Effects of device-assisted practice of activities of daily living in a close-to-normal pattern on upper extremity motor recovery in individuals with moderate to severe stroke: study protocol of a randomized control trial [5]	2025	sensors, actuators, control, AI, hand, wrist, finger, elbow, shoulder, arm-general, rigid-exoskeleton, study-RCT, study-clinical-trial, study-protocol, force-torque-sensing, setting-inpatient, outcome-FMA-UE, outcome-ARAT, outcome-BBT, outcome-MAL, stage-subacute, stage-chronic, actuation-motorized
Hybrid BCI for upper limb rehabilitation: integrating MI with peripheral field SSVEP stimulation [6]	2025	sensors, actuators, control, AI, hand, finger, glove, soft-exosuit, study-pilot-feasibility, study-protocol, study-technical, EEG-BCI, vision-based, setting-lab, stage-chronic
Robot-Assisted Mirror Rehabilitation for Post-Stroke Upper Limbs: A Personalized Control Strategy [7]	2025	sensors, actuators, control, wrist, elbow, shoulder, arm-general, multi-joint, rigid-exoskeleton, hybrid, study-pilot-feasibility, study-protocol, study-technical, EMG-interface, IMU-kine, actuation-motorized, control-impedance, control-admittance, control-PID
Loss of Joint Individuation and Abnormal Synergy Post Stroke in Upper Limb Movements [8]	2025	wrist, elbow, shoulder, arm-general, multi-joint, rigid-exoskeleton, study-observational, study-technical, outcome-FMA-UE, outcome-ARAT, outcome-MAS, actuation-motorized, control-impedance
Development and Efficacy Assessment of an Angle Sensor-Integrated Upper Limb Exoskeleton System for Autonomous Rehabilitation Training [9]	2025	sensors, actuators, control, wrist, elbow, shoulder, arm-general, rigid-exoskeleton, hybrid, study-protocol, study-technical, vision-based, IMU-kine, setting-lab, actuation-motorized
Enhancing Upper Limb Exoskeletons Using Sensor-Based Deep Learning Torque Prediction and PID Control [10]	2025	sensors, actuators, control, AI, elbow, arm-general, rigid-exoskeleton, study-technical, EMG-interface, force-torque-sensing, setting-lab, actuation-motorized, control-PID
Integration of neuromuscular control for multidirectional horizontal planar reaching movements in a portable upper limb exoskeleton for enhanced stroke rehabilitation [11]	2025	sensors, actuators, control, elbow, shoulder, arm-general, multi-joint, rigid-exoskeleton, study-technical, setting-lab, actuation-motorized, control-PID
Rehabilitation with hybrid assistive limb improves upper limb paralysis in patients with cerebral hemorrhage by repairing axonal injury of the corticospinal tract [12]	2025	sensors, actuators, control, elbow, arm-general, rigid-exoskeleton, study-clinical-trial, EMG-interface, setting-inpatient, outcome-FMA-UE, stage-subacute, actuation-motorized
Assist-as-Needed Controller of a Rehabilitation Exoskeleton for Upper-Limb Natural Movements [13]	2025	sensors, actuators, control, AI, wrist, elbow, shoulder, arm-general, multi-joint, rigid-exoskeleton, study-technical, force-torque-sensing, setting-lab, actuation-motorized

Title	Year	Labels
Effectiveness of Powered Hand Exoskeleton on Upper Extremity Function in People with Chronic Stroke [14]	2025	actuators, home_telerehab, hand, finger, arm-general, rigid-exoskeleton, study-RCT, study-clinical-trial, setting-outpatient, outcome-FMA-UE, outcome-BBT, stage-chronic, actuation-motorized
Development and Control of an Upper-Limb Exoskeleton CASIA-EXO for Motor Learning in Post-Stroke Rehabilitation [15]	2025	sensors, actuators, control, wrist, elbow, shoulder, arm-general, multi-joint, rigid-exoskeleton, setting-home, actuation-cabled, control-admittance, control-adaptive
Design of a Variable-Stiffness Exoskeleton for Upper Limb Rehabilitation [16]	2025	actuators, home_telerehab, wrist, elbow, shoulder, arm-general, multi-joint, rigid-exoskeleton, study-technical, setting-lab, actuation-VSA, actuation-cabled, actuation-motorized
SOFT ROBOTIC GLOVES VERSUS MIRROR THERAPY: A LONG-TERM COMPARATIVE STUDY ON HAND FUNCTION AND MOTOR RECOVERY IN POST-STROKE REHABILITATION [17]	2025	actuators, hand, finger, arm-general, glove, soft-exosuit, study-clinical-trial, study-pilot-feasibility, setting-outpatient, outcome-ARAT, outcome-NHPT, actuation-SEA, control-impedance
Poststroke Neurorehabilitation Using a Soft Robotic Glove Combined With a Virtual Environment: Preliminary Study on Feasibility, Safety, Effects, and User Satisfaction [18]	2025	sensors, actuators, control, home_telerehab, hand, finger, arm-general, glove, rigid-exoskeleton, study-clinical-trial, study-pilot-feasibility, setting-inpatient, outcome-FMA-UE, outcome-ARAT, outcome-BBT, stage-subacute, actuation-motorized, control-adaptive
THE EFFECT OF SOFT ROBOTIC GLOVE ON THE FLEXION AND EXTENSION OF HAND FOR STROKE PATIENTS: A CLINICAL TRIAL [19]	2025	sensors, actuators, home_telerehab, hand, finger, arm-general, glove, soft-exosuit, study-clinical-trial, study-pilot-feasibility, force-torque-sensing, setting-outpatient, outcome-ARAT, outcome-BBT, stage-subacute, stage-chronic, actuation-cabled, actuation-motorized
Up-Care: An Upper Limb Portable Cable Assistive Recovery Exoskeleton for Elbow Movement Assistance [20]	2025	sensors, actuators, control, home_telerehab, elbow, arm-general, rigid-exoskeleton, study-technical, force-torque-sensing, setting-lab, actuation-SEA, actuation-cabled, actuation-motorized, control-PID
Robotic Exoskeleton for Upper Arm Stroke Rehabilitation [21]	2025	sensors, actuators, control, elbow, arm-general, rigid-exoskeleton, study-technical, setting-lab, actuation-motorized
A lightweight, fully wearable 7-DOF upper limb exoskeleton for home-based rehabilitation: design, modeling, and experimental validation [22]	2025	sensors, actuators, control, AI, wrist, elbow, shoulder, arm-general, multi-joint, rigid-exoskeleton, study-technical, vision-based, setting-home, actuation-hydraulic, actuation-cabled, control-admittance
A Novel Low-Pressure Robotic Glove Based on CT-Optimized Finger Joint Kinematic Model for Long-Term Rehabilitation of Stroke Patients [23]	2024	sensors, actuators, control, home_telerehab, hand, finger, soft-exosuit, study-review, setting-home, actuation-cabled
B-FMEA-TRIZ model for scheme decision in conceptual product design: A study on upper-limb hemiplegia rehabilitation exoskeleton [24]	2024	sensors, actuators, control, elbow, shoulder, arm-general, multi-joint, rigid-exoskeleton, study-technical, vision-based, setting-home, actuation-cabled, control-admittance
Elbow-Hand Robotic Exoskeletons for Active and Passive Rehabilitation on Post-Stroke Patients: A Bioengineering Review [25]	2024	sensors, actuators, control, AI, home_telerehab, hand, elbow, arm-general, multi-joint, glove, soft-exosuit, rigid-exoskeleton, hybrid, study-review, EMG-interface, EEG-BCI, force-torque-sensing, setting-inpatient, setting-outpatient, setting-home, outcome-FMA-UE, outcome-ARAT, outcome-BBT, outcome-FIM, outcome-MAS, stage-acute, stage-subacute, stage-chronic, actuation-PAM, actuation-SMA, actuation-hydraulic, actuation-cabled, actuation-motorized, control-impedance, control-admittance, control-adaptive, AI-intent-decoding

Title	Year	Labels
Gravity support from a robotic exoskeleton increases spontaneous use of the nondominant upper extremity during a choice reaching task [26]	2024	sensors, actuators, control, elbow, shoulder, arm-general, multi-joint, rigid-exoskeleton, study-pilot-feasibility, force-torque-sensing, stage-chronic, actuation-cabled
Neural Interface-Based Motor Neuroprosthesis in Poststroke Upper Limb Neurorehabilitation: An Individual Patient Data Meta-analysis [27]	2024	sensors, actuators, control, AI, hand, wrist, finger, elbow, shoulder, arm-general, multi-joint, glove, rigid-exoskeleton, hybrid, study-protocol, study-technical, EMG-interface, setting-outpatient, setting-home, outcome-FMA-UE, outcome-ARAT, outcome-WMFT, stage-acute, stage-subacute, actuation-PAM, actuation-SMA, actuation-hydraulic, control-RL
The efficacy of hybrid neuroprostheses in the rehabilitation of upper limb impairment after stroke, a narrative and systematic review with a meta-analysis [28]	2024	sensors, actuators, control, AI, home_telerehab, hand, wrist, finger, elbow, shoulder, arm-general, multi-joint, glove, rigid-exoskeleton, hybrid, study-observational, study-review, study-technical, vision-based, IMU-kine, setting-inpatient, setting-outpatient, setting-home, outcome-FMA-UE, outcome-ARAT, outcome-BBT, outcome-NIHSS, stage-chronic, actuation-SEA, actuation-PAM, actuation-motorized, control-admittance, control-adaptive
Therapy for Abnormal Muscle Synergies in Stroke Using the ULIX Low-Impedance Robot [29]	2024	sensors, actuators, control, elbow, shoulder, arm-general, multi-joint, soft-exosuit, study-clinical-trial, study-pilot-feasibility, setting-inpatient, setting-lab, actuation-VSA, actuation-hydraulic
Kinematic and Dynamic Analysis of the Cable Actuation in an Upper-Extremity Exoskeleton [30]	2024	sensors, actuators, elbow, shoulder, arm-general, multi-joint, rigid-exoskeleton, study-technical, setting-lab, actuation-cabled, actuation-motorized
Design and Control of a Cable-Driven Exoskeleton System for Upper-Extremity Rehabilitation [31]	2024	sensors, actuators, control, elbow, shoulder, arm-general, multi-joint, rigid-exoskeleton, study-technical, force-torque-sensing, setting-lab, actuation-cabled, actuation-motorized, control-impedance, control-admittance, control-PID
Mechanical Design and Analysis of Cable-Driven Wearable Flexible Exoskeleton System [32]	2024	sensors, actuators, elbow, shoulder, arm-general, multi-joint, soft-exosuit, study-review, setting-home, actuation-cabled, actuation-motorized
Automatic Prediction of Upper Limb Motor Recovery in Stroke Patients Undergoing Robotic Therapy [33]	2024	sensors, actuators, control, AI, elbow, shoulder, arm-general, multi-joint, rigid-exoskeleton, study-clinical-trial, study-pilot-feasibility, setting-inpatient, outcome-FMA-UE, stage-subacute, actuation-motorized
Emerging trends in human upper extremity rehabilitation robot [34]	2024	sensors, actuators, control, home_telerehab, hand, wrist, finger, elbow, shoulder, arm-general, multi-joint, soft-exosuit, rigid-exoskeleton, study-review, EMG-interface, vision-based, IMU-kine, setting-inpatient, setting-outpatient, setting-home, setting-lab, actuation-SEA, actuation-PAM, actuation-SMA, actuation-hydraulic, actuation-cabled, actuation-motorized, control-impedance, control-admittance, control-adaptive
Restoring of Interhemispheric Symmetry in Patients With Stroke Following Bilateral or Unilateral Robot-Assisted Upper-Limb Rehabilitation: A Pilot Randomized Controlled Trial [35]	2024	sensors, actuators, control, elbow, shoulder, arm-general, multi-joint, rigid-exoskeleton, study-RCT, study-clinical-trial, study-pilot-feasibility, setting-inpatient, outcome-FMA-UE, outcome-ARAT, outcome-WMFT, outcome-MAS, stage-subacute, actuation-motorized
A 4-DOF Exosuit Using a Hybrid EEG-Based Control Approach for Upper-Limb Rehabilitation [36]	2024	sensors, actuators, control, AI, hand, wrist, finger, elbow, shoulder, arm-general, multi-joint, soft-exosuit, study-clinical-trial, study-pilot-feasibility, IMU-kine, actuation-VSA, actuation-PAM, control-adaptive

Title	Year	Labels
Robot-Assisted Therapies for Upper Limb Stroke Rehabilitation: A Narrative Review [37]	2024	sensors, actuators, hand, wrist, finger, elbow, shoulder, arm-general, multi-joint, rigid-exoskeleton, study-protocol, setting-inpatient, setting-outpatient, outcome-FMA-UE, outcome-MAL, outcome-FIM, stage-acute, stage-subacute, stage-chronic, actuation-cabled
A Novel Bilateral Underactuated Upper Limb Exoskeleton for Post-Stroke Bimanual ADL Training [38]	2024	sensors, actuators, control, hand, wrist, elbow, shoulder, arm-general, multi-joint, rigid-exoskeleton, EMG-interface, vision-based, setting-inpatient, outcome-ARAT, actuation-hydraulic, control-adaptive, control-MPC
Design and Modelling of a Human Upper Limb for Rehabilitation Exoskeleton [39]	2024	sensors, actuators, hand, wrist, elbow, shoulder, arm-general, multi-joint, rigid-exoskeleton, study-technical, setting-lab, actuation-motorized
Portable robots for upper-limb rehabilitation after stroke: a systematic review and meta-analysis [40]	2024	sensors, actuators, home_telerehab, hand, wrist, finger, elbow, shoulder, arm-general, multi-joint, glove, soft-exosuit, hybrid, study-RCT, study-technical, EMG-interface, EEG-BCI, force-torque-sensing, setting-outpatient, setting-lab, outcome-FMA-UE, outcome-ARAT, outcome-WMFT, outcome-NHPT, outcome-MAL, outcome-FIM, outcome-Barthel, outcome-MAS, outcome-NIHSS, stage-acute, stage-subacute, stage-chronic, actuation-SEA, actuation-VSA, actuation-hydraulic, control-impedance, control-adaptive, control-PID
An Upper Limb Exoskeleton Motion Generation Algorithm Based on Separating Shoulder and Arm Motion [41]	2024	sensors, actuators, control, AI, wrist, elbow, shoulder, arm-general, multi-joint, rigid-exoskeleton, study-technical, vision-based, setting-lab, actuation-motorized
Design and Control of 2DoF Wearable Robot Arm for Rehabilitation [42]	2024	sensors, actuators, control, elbow, shoulder, arm-general, multi-joint, rigid-exoskeleton, study-technical, setting-lab, actuation-motorized, control-adaptive, control-PID
Initial Testing of Robotic Exoskeleton Hand Device for Stroke Rehabilitation [43]	2023	sensors, actuators, control, wrist, elbow, soft-exosuit, study-review, IMU-kine, setting-home, actuation-hydraulic, actuation-cabled
Occupational therapists' evaluation of the perceived usability and utility of wearable soft robotic exoskeleton gloves for hand function rehabilitation following a stroke [44]	2023	sensors, actuators, hand, finger, glove, study-observational, setting-lab, actuation-cabled, actuation-motorized
Functional MRI Assessment of Brain Activity During Hand Rehabilitation with an MR-Compatible Soft Glove in Chronic Stroke Patients: A Preliminary Study [45]	2023	sensors, actuators, control, AI, hand, finger, multi-joint, glove, study-clinical-trial, study-pilot-feasibility, EEG-BCI, vision-based, outcome-FMA-UE, stage-chronic, actuation-cabled, AI-intent-decoding
Hand rehabilitation based on the RobHand exoskeleton in stroke patients: A case series study [46]	2023	actuators, hand, finger, glove, study-clinical-trial, study-case, IMU-kine, setting-outpatient, setting-home, outcome-ARAT, actuation-VSA
User-centered development process of an operating interface to couple a robotic glove with a virtual environment to optimize hand rehabilitation following a stroke [47]	2023	sensors, actuators, hand, finger, glove, rigid-exoskeleton, study-observational, IMU-kine, force-torque-sensing, setting-lab, stage-chronic, actuation-cabled, actuation-motorized
A bidirectional fabric-based soft robotic glove for hand function assistance in patients with chronic stroke [48]	2023	sensors, actuators, control, home_telerehab, hand, finger, glove, study-clinical-trial, study-case, setting-outpatient, stage-chronic, actuation-PAM
Validating the measurement of upper limb sensorimotor behavior utilizing a tablet in neurologically intact controls and individuals with chronic stroke [49]	2023	sensors, actuators, home_telerehab, hand, elbow, shoulder, arm-general, multi-joint, rigid-exoskeleton, study-case, setting-inpatient, setting-home, actuation-SEA, actuation-motorized

Title	Year	Labels
The NuroSleeve, a user-centered 3D printed hybrid orthosis for individuals with upper extremity impairment [50]	2023	sensors, actuators, control, home_telerehab, hand, finger, soft-exosuit, rigid-exoskeleton, study-clinical-trial, study-pilot-feasibility, EEG-BCI, force-torque-sensing, setting-outpatient, setting-lab, stage-chronic, actuation-motorized
The Application of Soft Robotic Gloves in Stroke Patients: A Systematic Review and Meta-Analysis of Randomized Controlled Trials [51]	2023	sensors, actuators, hand, wrist, finger, glove, study-observational, study-review, study-technical, EEG-BCI, vision-based, IMU-kine, setting-inpatient, setting-lab, outcome-Barthel, outcome-MAS, outcome-NIHSS
Upper Extremity Robotic-Assisted Rehabilitation: Results Not Yet Robust [52]	2023	sensors, actuators, control, hand, wrist, finger, elbow, shoulder, arm-general, multi-joint, soft-exosuit, rigid-exoskeleton, study-protocol, setting-inpatient, setting-lab, outcome-BBT, stage-subacute, stage-chronic, actuation-motorized
Exoskeleton-Assisted Anthropomorphic Movement Training for the Upper Limb After Stroke: The EAMT Randomized Trial [53]	2023	sensors, actuators, control, elbow, shoulder, arm-general, multi-joint, rigid-exoskeleton, study-RCT, study-clinical-trial, force-torque-sensing, setting-inpatient, outcome-FMA-UE, outcome-Barthel, stage-subacute, actuation-motorized, control-admittance
Neurophysiological and Clinical Effects of Upper Limb Robot-Assisted Rehabilitation on Motor Recovery in Patients with Subacute Stroke: A Multicenter Randomized Controlled Trial Study Protocol [54]	2023	sensors, actuators, finger, elbow, shoulder, arm-general, soft-exosuit, rigid-exoskeleton, study-RCT, study-clinical-trial, study-protocol, setting-inpatient, setting-lab, outcome-WMFT, outcome-BBT, outcome-FIM, outcome-Barthel, stage-acute, actuation-cabled
Lightweight Bioinspired Exoskeleton for Wrist Rehabilitation Powered by Twisted and Coiled Artificial Muscles [55]	2023	sensors, actuators, control, wrist, glove, study-technical, EMG-interface, IMU-kine, outcome-FMA-UE, actuation-SMA, control-PID
Effectiveness of soft robotic glove versus repetitive transcranial magnetic stimulation in post-stroke patients with severe upper limb dysfunction: A randomised controlled trial [56]	2023	sensors, actuators, home_telerehab, hand, wrist, finger, glove, study-RCT, study-clinical-trial, setting-inpatient, outcome-FMA-UE, outcome-Barthel, stage-subacute, actuation-PAM
Implementation of technological devices used by occupational therapists in upper extremity rehabilitation after a stroke; Implementaci¢n de dispositivos tecnol¢gicos usados por terapeutas ocupacionales en la rehabilitaci¢n de extremidad superior posterior a un accidente cerebro vascular [57]	2023	sensors, actuators, hand, wrist, finger, elbow, shoulder, arm-general, multi-joint, glove, rigid-exoskeleton, study-protocol, EMG-interface, force-torque-sensing, stage-subacute, actuation-SEA, actuation-motorized
A Self-Aligning Upper-Limb Exoskeleton Preserving Natural Shoulder Movements: Kinematic Compatibility Analysis [58]	2023	sensors, actuators, control, elbow, shoulder, arm-general, multi-joint, rigid-exoskeleton, study-technical, setting-lab, actuation-SEA, actuation-motorized
Delayed Muscle Activity in Stroke Survivors with Upper-Limb Hemiparesis [59]	2023	sensors, home_telerehab, wrist, study-pilot-feasibility, study-protocol, force-torque-sensing, outcome-BBT
An Upper-Limb Rehabilitation Exoskeleton System Controlled by MI Recognition Model With Deep Emphasized Informative Features in a VR Scene [60]	2023	sensors, actuators, control, AI, hand, wrist, finger, elbow, shoulder, arm-general, multi-joint, rigid-exoskeleton, study-technical, EEG-BCI, setting-lab, actuation-motorized, AI-intent-decoding
Development and Validation of a Kinematically Accurate Upper-Limb Exoskeleton Digital Twin for Stroke Rehabilitation [61]	2023	sensors, actuators, hand, finger, elbow, shoulder, arm-general, multi-joint, rigid-exoskeleton, study-technical, IMU-kine, setting-lab, actuation-motorized
Direction-dependent differences in the quality and quantity of horizontal reaching in people after stroke [62]	2023	sensors, actuators, elbow, shoulder, arm-general, multi-joint, soft-exosuit, study-pilot-feasibility, vision-based, setting-inpatient, stage-chronic
Adaptive PID Controller Based on Sliding Surface for Controlling Elbow Joint Robot Rehabilitation [63]	2023	sensors, actuators, control, wrist, glove, study-protocol, setting-outpatient, actuation-hydraulic, control-impedance, control-admittance

Title	Year	Labels
Development of Elbow Joint Exoskeleton Control System Using Fuzzy Pid Control Method for Post Stroke Rehabilitation [64]	2023	sensors, actuators, control, elbow, rigid-exoskeleton, study-technical, force-torque-sensing, setting-home, actuation-cabled, control-admittance
Design and Performance Analysis of a Bioelectronic Controlled Hybrid Serial-Parallel Wrist Exoskeleton [65]	2023	sensors, actuators, control, AI, hand, wrist, finger, glove, rigid-exoskeleton, study-technical, EMG-interface, IMU-kine, force-torque-sensing, setting-lab, actuation-motorized, control-PID, AI-intent-decoding
Integrating hand exoskeletons into goal-oriented clinic and home stroke and spinal cord injury rehabilitation [66]	2022	sensors, actuators, control, home_telerehab, hand, finger, glove, study-clinical-trial, study-pilot-feasibility, study-case, IMU-kine, setting-outpatient, setting-home, outcome-FMA-UE, outcome-BBT, outcome-MAL, outcome-FIM, outcome-MAS, stage-subacute, stage-chronic, actuation-cabled, actuation-motorized
May Dual Transcranial Direct Current Stimulation Enhance the Efficacy of Robot-Assisted Therapy for Promoting Upper Limb Recovery in Chronic Stroke? [67]	2022	sensors, actuators, wrist, elbow, shoulder, arm-general, multi-joint, rigid-exoskeleton, study-RCT, study-clinical-trial, setting-outpatient, outcome-FMA-UE, outcome-Barthel, outcome-MAS, outcome-NIHSS, stage-chronic, actuation-motorized
A randomized clinical control study on the efficacy of three-dimensional upper limb robotic exoskeleton training in chronic stroke [68]	2022	sensors, actuators, control, wrist, elbow, shoulder, arm-general, multi-joint, rigid-exoskeleton, study-RCT, study-clinical-trial, setting-outpatient, outcome-FMA-UE, outcome-MAS, stage-chronic, actuation-cabled, actuation-motorized, control-impedance
Human-Exoskeleton Joint Coordination Assessment: A Case Study on the Shoulder and Elbow Joints [69]	2022	actuators, elbow, shoulder, arm-general, multi-joint, rigid-exoskeleton, study-technical, setting-lab, actuation-motorized
Development of an EMG-based Elbow-Exoskeleton with Twisted String Actuation [70]	2022	sensors, actuators, control, home_telerehab, elbow, rigid-exoskeleton, study-technical, EMG-interface, force-torque-sensing, setting-lab, actuation-cabled, actuation-motorized, control-PID
Supporting and Stabilizing the Scapulohumeral Rhythm With a Body- or Robot-Powered Orthosis [71]	2022	sensors, actuators, control, home_telerehab, elbow, shoulder, arm-general, multi-joint, soft-exosuit, rigid-exoskeleton, study-technical, vision-based, IMU-kine, force-torque-sensing, setting-lab, actuation-SEA, actuation-cabled, actuation-motorized, control-impedance
Upper extremity exoskeleton system to generate customized therapy motions for stroke survivors [72]	2022	sensors, actuators, control, elbow, shoulder, arm-general, multi-joint, rigid-exoskeleton, study-technical, setting-lab, actuation-motorized
Thumb Stabilization and Assistance in a Robotic Hand Orthosis for Post-Stroke Hemiparesis [73]	2022	sensors, actuators, control, wrist, elbow, soft-exosuit, study-review, IMU-kine, setting-outpatient, actuation-SEA, actuation-motorized, control-impedance, control-PID
Comparative analysis of three categories of four-DOFs exoskeleton mechanism based on relative movement offsets [74]	2022	elbow, shoulder, arm-general, multi-joint, rigid-exoskeleton, study-technical, setting-lab
Robustness and Tracking Performance Evaluation of PID Motion Control of 7 DoF Anthropomorphic Exoskeleton Robot Assisted Upper Limb Rehabilitation [75]	2022	sensors, actuators, control, wrist, elbow, shoulder, arm-general, multi-joint, rigid-exoskeleton, study-technical, force-torque-sensing, setting-lab, actuation-motorized, control-PID
Probabilistic Model-Based Learning Control of a Soft Pneumatic Glove for Hand Rehabilitation [76]	2022	sensors, actuators, control, AI, hand, finger, glove, study-clinical-trial, study-pilot-feasibility, study-case, EMG-interface, setting-lab, outcome-FMA-UE, outcome-ARAT, outcome-BBT, outcome-MAS, stage-chronic, actuation-PAM, control-adaptive

Title	Year	Labels
An Exoneuromusculoskeleton for Self-Help Upper Limb Rehabilitation after Stroke [77]	2022	sensors, actuators, control, hand, wrist, finger, elbow, arm-general, multi-joint, glove, soft-exosuit, hybrid, study-clinical-trial, study-pilot-feasibility, EMG-interface, vision-based, setting-lab, outcome-FMA-UE, outcome-ARAT, outcome-WMFT, outcome-FIM, outcome-MAS, stage-chronic, actuation-PAM
Neuromuscular electrical stimulation restores upper limb sensory-motor functions and body representations in chronic stroke survivors [78]	2022	actuators, hand, wrist, finger, glove, study-clinical-trial, setting-inpatient, outcome-BBT, outcome-MAL, stage-chronic
NASA-TLX Assessment for a Haptic Adaptive Platform for Upper Extremity Motor Rehabilitation [79]	2022	sensors, actuators, control, AI, hand, finger, elbow, shoulder, arm-general, multi-joint, glove, rigid-exoskeleton, study-technical, setting-home, actuation-cabled, control-impedance, control-admittance
Exoskeleton Device for Left Hemiplegia Patients with Electromyograph [80]	2022	sensors, actuators, control, home_telerehab, elbow, rigid-exoskeleton, study-technical, IMU-kine, setting-lab, actuation-motorized
AGREE: an upper-limb robotic platform for personalized rehabilitation, concept and clinical study design [81]	2022	sensors, actuators, control, elbow, shoulder, arm-general, multi-joint, rigid-exoskeleton, study-RCT, study-clinical-trial, study-protocol, force-torque-sensing, setting-inpatient, outcome-FMA-UE, outcome-ARAT, outcome-BBT, outcome-MAS, stage-subacute, stage-chronic, actuation-motorized, control-impedance
Impact of Gravity Compensation on Upper Extremity Movements in Harmony Exoskeleton [82]	2022	sensors, actuators, control, elbow, shoulder, arm-general, multi-joint, rigid-exoskeleton, study-pilot-feasibility, study-case, study-observational, force-torque-sensing, setting-outpatient, outcome-ARAT, actuation-hydraulic, actuation-cabled, control-MPC
Measuring Torque Production with a Robotic Exoskeleton during Cervical Transcutaneous Spinal Stimulation [83]	2022	sensors, actuators, control, wrist, elbow, arm-general, multi-joint, rigid-exoskeleton, study-technical, setting-lab, actuation-motorized
Design of a 6-DoF Cost-effective Differential-drive based Robotic system for Upper-Limb Stroke Rehabilitation [84]	2022	sensors, actuators, control, home_telerehab, wrist, elbow, shoulder, arm-general, multi-joint, study-technical, vision-based, IMU-kine, force-torque-sensing, setting-lab, actuation-motorized, control-PID
The effect of mirror therapy can be improved by simultaneous robotic assistance [85]	2022	actuators, hand, finger, glove, study-RCT, study-clinical-trial, setting-inpatient, outcome-FMA-UE, stage-subacute, stage-chronic, actuation-cabled, actuation-motorized
SSVEP-Based Brain Computer Interface Controlled Soft Robotic Glove for Post-Stroke Hand Function Rehabilitation [86]	2022	sensors, actuators, control, hand, finger, glove, study-RCT, study-clinical-trial, EEG-BCI, setting-inpatient, outcome-FMA-UE, outcome-WMFT, outcome-MAS, stage-chronic, actuation-PAM
Exoskeleton versus end-effector robot-assisted therapy for finger-hand motor recovery in stroke survivors: systematic review and meta-analysis [87]	2022	study-review
Dynamic hand orthoses for the recovery of hand and arm function in adults after stroke: A systematic review and meta-analysis of randomised controlled trials [88]	2022	study-review
Evidence of neuroplasticity with robotic hand exoskeleton for post-stroke rehabilitation: a randomized controlled trial [89]	2021	sensors, actuators, control, hand, wrist, finger, multi-joint, rigid-exoskeleton, study-RCT, study-clinical-trial, study-pilot-feasibility, EMG-interface, setting-outpatient, outcome-FMA-UE, outcome-Barthel, outcome-MAS, stage-subacute, stage-chronic, actuation-motorized

Title	Year	Labels
Early post-stroke rehabilitation for upper limb motor function using virtual reality and exoskeleton: equally efficient in older patients [90]	2021	sensors, home_telerehab, finger, elbow, shoulder, arm-general, soft-exosuit, hybrid, study-RCT, force-torque-sensing, setting-home, outcome-WMFT, outcome-FIM, outcome-NIHSS
Exoskeleton-Assisted Anthropomorphic Movement Training (EAMT) for Poststroke Upper Limb Rehabilitation: A Pilot Randomized Controlled Trial [91]	2021	sensors, actuators, control, wrist, elbow, shoulder, arm-general, multi-joint, soft-exosuit, hybrid, study-RCT, study-clinical-trial, IMU-kine, force-torque-sensing, outcome-ARAT, outcome-FIM, stage-acute, control-impedance
Design and Validation of a Self-Aligning Index Finger Exoskeleton for Post-Stroke Rehabilitation [92]	2021	sensors, actuators, control, wrist, glove, study-protocol, study-review, setting-inpatient, actuation-SMA, actuation-hydraulic
Effect of proprioceptive stimulation using a soft robotic glove on motor activation and brain connectivity in stroke survivors [93]	2021	actuators, control, hand, finger, glove, soft-exosuit, study-RCT, study-clinical-trial, setting-home, stage-subacute, actuation-SEA
A compact and lightweight rehabilitative exoskeleton to restore grasping functions for people with hand paralysis [94]	2021	sensors, actuators, control, home_telerehab, hand, finger, soft-exosuit, study-review, study-technical, setting-outpatient, stage-acute, actuation-SMA
Design and development of an upper limb rehabilitative robot with dual functionality [95]	2021	sensors, actuators, control, wrist, elbow, shoulder, arm-general, multi-joint, soft-exosuit, study-review, vision-based, setting-inpatient, actuation-hydraulic, control-impedance
Research on the Kinematic Compatibility of Position Solution of a Four-DOFs Mechanical Structure Mechanism [96]	2021	elbow, shoulder, arm-general, multi-joint, rigid-exoskeleton, study-technical, setting-lab
Brain-Computer Interface Coupled to a Robotic Hand Orthosis for Stroke Patients? Neurorehabilitation: A Crossover Feasibility Study [97]	2021	actuators, control, hand, finger, soft-exosuit, hybrid, study-RCT, study-clinical-trial, EMG-interface, IMU-kine, setting-outpatient, setting-home, outcome-MAS, outcome-NIHSS, actuation-hydraulic
Sensing and Control of a Multi-Joint Soft Wearable Robot for Upper-Limb Assistance and Rehabilitation [98]	2021	sensors, actuators, control, home_telerehab, elbow, shoulder, arm-general, multi-joint, soft-exosuit, study-technical, IMU-kine, setting-lab, actuation-hydraulic, control-MPC
Clinical Test of a Wearable, High DOF, Spring Powered Hand Exoskeleton (HandSOME II) [99]	2021	sensors, home_telerehab, hand, finger, multi-joint, rigid-exoskeleton, study-clinical-trial, study-pilot-feasibility, vision-based, setting-lab, stage-chronic
Preliminary Assessment of a Postural Synergy-Based Exoskeleton for Post-Stroke Upper Limb Rehabilitation [100]	2021	sensors, actuators, control, elbow, shoulder, arm-general, multi-joint, rigid-exoskeleton, study-clinical-trial, study-pilot-feasibility, force-torque-sensing, setting-inpatient, outcome-FMA-UE, outcome-ARAT, outcome-Barthel, stage-subacute, actuation-cabled, actuation-motorized, control-admittance
Highlighting gaps in spinal cord injury research in activity-based interventions for the upper extremity: A scoping review [101]	2021	study-observational
The adjunct of transcranial direct current stimulation to Robot-assisted therapy in upper limb post-stroke treatment [102]	2021	study-observational
HandMATE: Wearable Robotic Hand Exoskeleton and Integrated Android App for At Home Stroke Rehabilitation [103]	2020	sensors, actuators, control, home_telerehab, hand, finger, multi-joint, rigid-exoskeleton, study-technical, force-torque-sensing, setting-lab, stage-chronic, actuation-motorized, control-admittance
Characterization and wearability evaluation of a fully portable wrist exoskeleton for unsupervised training after stroke [104]	2020	sensors, actuators, control, home_telerehab, wrist, rigid-exoskeleton, study-technical, force-torque-sensing, setting-lab, stage-chronic, actuation-motorized, control-admittance
Comparisons between end-effector and exoskeleton rehabilitation robots regarding upper extremity function among chronic stroke patients with moderate-to-severe upper limb impairment [105]	2020	actuators, wrist, elbow, shoulder, arm-general, multi-joint, rigid-exoskeleton, study-RCT, study-clinical-trial, setting-inpatient, outcome-FMA-UE, outcome-WMFT, stage-chronic, actuation-motorized

Title	Year	Labels
HERCULES: A Three Degree-of-Freedom Pneumatic Upper Limb Exoskeleton for Stroke Rehabilitation() [106]	2020	sensors, actuators, control, elbow, shoulder, arm-general, multi-joint, rigid-exoskeleton, study-technical, setting-lab, control-PID
Brain-Computer Interface-Based Soft Robotic Glove Rehabilitation for Stroke [107]	2020	actuators, control, hand, finger, multi-joint, glove, soft-exosuit, study-RCT, study-clinical-trial, study-pilot-feasibility, EEG-BCI, setting-outpatient, outcome-FMA-UE, outcome-ARAT, stage-chronic, actuation-PAM
User-Driven Functional Movement Training with a Wearable Hand Robot after Stroke [108]	2020	sensors, actuators, control, hand, finger, multi-joint, rigid-exoskeleton, study-clinical-trial, study-pilot-feasibility, EMG-interface, force-torque-sensing, setting-outpatient, outcome-FMA-UE, outcome-ARAT, outcome-BBT, stage-chronic, actuation-cabled, actuation-motorized, control-PID
A multimodal approach to capture post-stroke temporal dynamics of recovery [109]	2020	sensors, actuators, control, elbow, shoulder, arm-general, multi-joint, rigid-exoskeleton, study-clinical-trial, study-pilot-feasibility, setting-inpatient, setting-outpatient, outcome-FMA-UE, stage-subacute, actuation-motorized
Inflatable Soft Wearable Robot for Reducing Therapist Fatigue during Upper Extremity Rehabilitation in Severe Stroke [110]	2020	sensors, actuators, shoulder, soft-exosuit, study-clinical-trial, study-pilot-feasibility, vision-based, setting-lab, stage-chronic, actuation-PAM
Hand Extension Robot Orthosis (HERO) Grip Glove: Enabling independence amongst persons with severe hand impairments after stroke [111]	2020	sensors, actuators, control, home_telerehab, hand, finger, multi-joint, glove, soft-exosuit, study-clinical-trial, study-pilot-feasibility, IMU-kine, setting-inpatient, setting-outpatient, outcome-BBT, stage-acute, stage-chronic, actuation-cabled, actuation-motorized
Neural activity modulations and motor recovery following brain-exoskeleton interface mediated stroke rehabilitation [112]	2020	sensors, actuators, control, AI, elbow, rigid-exoskeleton, study-clinical-trial, study-pilot-feasibility, EMG-interface, EEG-BCI, setting-outpatient, outcome-FMA-UE, outcome-ARAT, stage-chronic, actuation-motorized, AI-intent-decoding
Dynamic compensation controller with feedback linearization technique of 3 degree of freedom exoskeleton robotic arm for upper limb rehabilitation purpose [113]	2020	actuators, control, wrist, elbow, shoulder, arm-general, multi-joint, rigid-exoskeleton, study-technical, setting-lab, actuation-motorized, control-PID
A portable assist-as-need upper-extremity hybrid exoskeleton for FES-induced muscle fatigue reduction in stroke rehabilitation [114]	2019	sensors, actuators, control, elbow, rigid-exoskeleton, hybrid, study-technical, force-torque-sensing, setting-lab, actuation-cabled, actuation-motorized, control-adaptive, control-PID
Robotic Exoskeleton for Wrist and Fingers Joint in Post-Stroke Neuro-Rehabilitation for Low-Resource Settings [115]	2019	sensors, actuators, control, home_telerehab, hand, wrist, finger, multi-joint, rigid-exoskeleton, study-clinical-trial, study-pilot-feasibility, EMG-interface, setting-outpatient, outcome-FMA-UE, outcome-Barthel, outcome-MAS, stage-chronic, actuation-motorized
An Elbow Exoskeleton for Upper Limb Rehabilitation with Series Elastic Actuator and Cable-Driven Differential [116]	2019	sensors, actuators, control, elbow, arm-general, multi-joint, rigid-exoskeleton, study-technical, force-torque-sensing, setting-lab, actuation-SEA, actuation-cabled, actuation-motorized, control-PID
A Hybrid robotic system for arm training of stroke survivors: Concept and first evaluation [117]	2019	sensors, actuators, control, elbow, shoulder, arm-general, multi-joint, rigid-exoskeleton, hybrid, study-clinical-trial, study-pilot-feasibility, EMG-interface, setting-inpatient, setting-outpatient, outcome-ARAT, outcome-BBT, outcome-MAL, stage-subacute
Movement kinematics and proprioception in post-stroke spasticity: Assessment using the Kinarm robotic exoskeleton [118]	2019	sensors, actuators, elbow, shoulder, arm-general, multi-joint, rigid-exoskeleton, study-observational, setting-lab, outcome-MAS, stage-chronic, actuation-motorized

Title	Year	Labels
Design and Construction of a Planar Robotic Exoskeleton for Assessment of Upper Limb Movements [119]	2019	sensors, elbow, shoulder, arm-general, multi-joint, rigid-exoskeleton, study-technical, setting-lab
Design and human?machine compatibility analysis of Co-Exos II for upper-limb rehabilitation [120]	2019	sensors, actuators, control, elbow, shoulder, arm-general, multi-joint, rigid-exoskeleton, study-technical, force-torque-sensing, setting-lab, actuation-motorized
A novel telerehabilitation system based on bilateral upper limb exoskeleton robot [121]	2019	sensors, actuators, control, AI, home_telerehab, wrist, elbow, shoulder, arm-general, multi-joint, rigid-exoskeleton, study-technical, EMG-interface, IMU-kine, setting-lab, actuation-motorized, AI-intent-decoding
Effect of repetitive transcranial magnetic stimulation combined with robot-assisted training on wrist muscle activation post-stroke [122]	2019	sensors, actuators, control, wrist, rigid-exoskeleton, study-clinical-trial, study-pilot-feasibility, EMG-interface, force-torque-sensing, setting-lab, stage-chronic, actuation-motorized
Active Upper Arm Exoskeleton - Design and Kinematic Analysis [123]	2019	actuators, wrist, elbow, shoulder, arm-general, multi-joint, rigid-exoskeleton, study-technical, setting-lab, actuation-motorized
Passive elbow movement assistant (PEMA): A portable exoskeleton to compensate angle-dependent tone profile of the elbow joint post-stroke [124]	2019	sensors, home_telerehab, elbow, rigid-exoskeleton, study-technical, setting-lab
Exploring the capabilities of harmony for upper-limb stroke therapy [125]	2019	sensors, actuators, control, elbow, shoulder, arm-general, multi-joint, rigid-exoskeleton, study-clinical-trial, study-pilot-feasibility, force-torque-sensing, outcome-FMA-UE, outcome-ARAT, stage-subacute, stage-chronic, actuation-SEA, actuation-motorized, control-impedance, control-adaptive
A novel elbow pneumatic muscle actuator for exoskeleton arm in post-stroke rehabilitation [126]	2019	sensors, actuators, control, home_telerehab, elbow, soft-exosuit, study-technical, setting-lab, actuation-PAM, control-adaptive
Brain-machine interface of upper limb recovery in stroke patients rehabilitation: A systematic review [127]	2019	sensors, actuators, control, AI, hand, arm-general, study-review, EEG-BCI, AI-intent-decoding
Cartesian Trajectory Tracking of a 7-DOF Exoskeleton Robot Based on Human Inverse Kinematics [128]	2019	sensors, actuators, control, wrist, elbow, shoulder, arm-general, multi-joint, rigid-exoskeleton, study-technical, setting-lab, actuation-motorized, control-adaptive
Improvement of human?machine compatibility of upper-limb rehabilitation exoskeleton using passive joints [129]	2019	sensors, actuators, control, elbow, shoulder, arm-general, multi-joint, rigid-exoskeleton, study-technical, force-torque-sensing, setting-lab, actuation-motorized
Pilot Study of a Powered Exoskeleton for Upper Limb Rehabilitation Based on the Wheelchair [130]	2019	sensors, actuators, control, wrist, elbow, shoulder, multi-joint, rigid-exoskeleton, study-technical, actuation-motorized
Novel adaptive iterative observer based on integral backstepping control of a wearable robotic exoskeleton [131]	2019	sensors, actuators, control, wrist, elbow, shoulder, arm-general, multi-joint, rigid-exoskeleton, study-technical, setting-lab, actuation-motorized, control-adaptive
The control of an upper extremity exoskeleton for stroke rehabilitation by means of a hybrid active force control [132]	2019	sensors, actuators, control, elbow, rigid-exoskeleton, study-technical, setting-lab, actuation-motorized, control-PID
Home-based hand rehabilitation with a robotic glove in hemiplegic patients after stroke: a pilot feasibility study [133]	2018	actuators, control, home_telerehab, hand, finger, multi-joint, glove, soft-exosuit, study-clinical-trial, study-pilot-feasibility, setting-home, outcome-NHPT, outcome-Barthel, outcome-MAS, stage-subacute, stage-chronic, actuation-motorized

Title	Year	Labels
Design and Development of a Portable Exoskeleton for Hand Rehabilitation [134]	2018	sensors, actuators, control, AI, home_telerehab, hand, finger, multi-joint, rigid-exoskeleton, study-clinical-trial, study-pilot-feasibility, EMG-interface, outcome-FMA-UE, outcome-ARAT, outcome-WMFT, stage-chronic, actuation-motorized
Vision of the upper limb fails to compensate for kinesthetic impairments in subacute stroke [135]	2018	sensors, actuators, elbow, shoulder, arm-general, multi-joint, rigid-exoskeleton, study-observational, setting-inpatient, setting-lab, outcome-FIM, stage-subacute, actuation-motorized
A Virtual Reality Rehabilitation Training System Based on Upper Limb Exoskeleton Robot [136]	2018	sensors, actuators, control, wrist, elbow, shoulder, arm-general, multi-joint, rigid-exoskeleton, study-technical, IMU-kine, force-torque-sensing, setting-lab, actuation-motorized
The Retrainer Light-Weight Arm Exoskeleton: Effect of Adjustable Gravity Compensation on Muscle Activations and Forces [137]	2018	sensors, actuators, elbow, shoulder, arm-general, multi-joint, rigid-exoskeleton, study-technical, force-torque-sensing, setting-lab, actuation-cabled, actuation-motorized
Design of the speech control system for a upper limb rehabilitation robot based on wavelet denoising [138]	2018	sensors, actuators, control, home_telerehab, hand, wrist, finger, elbow, arm-general, multi-joint, rigid-exoskeleton, study-technical, setting-lab, actuation-motorized
Comparative study of actuation systems for portable upper limb exoskeletons [139]	2018	actuators, control, home_telerehab, hand, wrist, finger, elbow, shoulder, arm-general, multi-joint, glove, rigid-exoskeleton, hybrid, study-review, actuation-SEA, actuation-VSA, actuation-PAM, actuation-SMA, actuation-hydraulic, actuation-cabled, actuation-motorized
Bilateral reaching deficits after unilateral perinatal ischemic stroke: A population-based case-control study [140]	2018	sensors, elbow, shoulder, arm-general, multi-joint, rigid-exoskeleton, study-observational, setting-lab, outcome-MAS, stage-chronic
A Soft Exosuit for Flexible Upper-Extremity Rehabilitation [141]	2018	sensors, actuators, control, home_telerehab, wrist, elbow, shoulder, arm-general, multi-joint, soft-exosuit, study-pilot-feasibility, study-technical, IMU-kine, setting-home, setting-lab, actuation-cabled, actuation-motorized
Rehabilitation robots [142]	2018	hand, arm-general, rigid-exoskeleton, study-review
The Design of a Low-Cost Therapy Robot for Hand Rehabilitation of a Post-Stroke Patient [143]	2018	sensors, actuators, control, hand, finger, multi-joint, rigid-exoskeleton, study-technical, setting-lab, actuation-motorized, control-PID
Development of EMG based Upper Arm Exoskeleton [144]	2018	sensors, actuators, elbow, rigid-exoskeleton, study-technical, EMG-interface, setting-lab, actuation-motorized
Hand Motor Rehabilitation of Patients with Stroke Using Physiologically Congruent Neurofeedback [145]	2018	sensors, actuators, control, hand, finger, glove, soft-exosuit, study-clinical-trial, study-pilot-feasibility, EEG-BCI, outcome-FMA-UE, outcome-MAS, stage-subacute, stage-chronic, actuation-PAM
Evaluation of the harmony exoskeleton as an upper extremity rehabilitation tool after stroke [146]	2018	sensors, actuators, control, elbow, shoulder, arm-general, multi-joint, rigid-exoskeleton, study-case, setting-outpatient, stage-chronic, actuation-motorized, control-impedance
Development of a Minimal-Intervention-Based Admittance Control Strategy for Upper Extremity Rehabilitation Exoskeleton [147]	2018	sensors, actuators, control, wrist, elbow, shoulder, arm-general, multi-joint, rigid-exoskeleton, study-pilot-feasibility, study-technical, force-torque-sensing, setting-lab, outcome-FMA-UE, actuation-cabled, actuation-motorized, control-admittance
Finger strength, individuation, and their interaction: Relationship to hand function and corticospinal tract injury after stroke [148]	2018	sensors, actuators, control, hand, finger, rigid-exoskeleton, study-observational, force-torque-sensing, setting-lab, outcome-FMA-UE, outcome-BBT, outcome-NHPT, stage-chronic, actuation-motorized, control-impedance, control-adaptive

Title	Year	Labels
From the past to the future of therapeutic orthoses for upper limbs rehabilitation [149]	2018	sensors, actuators, control, home_telerehab, hand, wrist, finger, elbow, shoulder, arm-general, multi-joint, study-review
Design and implementation of shoulder exoskeleton robot [150]	2018	actuators, control, shoulder, rigid-exoskeleton, study-technical, setting-lab, actuation-motorized
Self-powered robots to reduce motor slacking during upper-extremity rehabilitation: A proof of concept study [151]	2018	sensors, actuators, elbow, arm-general, rigid-exoskeleton, study-pilot-feasibility, study-technical, setting-lab, outcome-FMA-UE, outcome-MAS, stage-chronic, actuation-cabled
The eWrist - A wearable wrist exoskeleton with sEMG-based force control for stroke rehabilitation [152]	2017	sensors, actuators, control, home_telerehab, wrist, rigid-exoskeleton, study-technical, EMG-interface, force-torque-sensing, setting-lab, actuation-motorized, control-admittance, control-PID
Applying a soft-robotic glove as assistive device and training tool with games to support hand function after stroke: Preliminary results on feasibility and potential clinical impact [153]	2017	sensors, actuators, control, home_telerehab, hand, finger, glove, soft-exosuit, study-pilot-feasibility, force-torque-sensing, setting-outpatient, setting-home, outcome-FMA-UE, stage-chronic, actuation-cabled, actuation-motorized
Post-stroke Rehabilitation Training with a Motor-Imagery-Based Brain-Computer Interface (BCI)-Controlled Hand Exoskeleton: A Randomized Controlled Multicenter Trial [154]	2017	sensors, actuators, control, AI, hand, finger, rigid-exoskeleton, study-clinical-trial, study-pilot-feasibility, IMU-kine, outcome-BBT, actuation-PAM, control-MPC
Improving Upper Extremity Function and Quality of Life with a Tongue Driven Exoskeleton: A Pilot Study Quantifying Stroke Rehabilitation [155]	2017	sensors, actuators, control, AI, hand, wrist, rigid-exoskeleton, study-pilot-feasibility, setting-lab, outcome-FMA-UE, outcome-WMFT, stage-chronic, actuation-PAM, AI-intent-decoding
A composite robotic-based measure of upper limb proprioception [156]	2017	sensors, actuators, elbow, shoulder, arm-general, multi-joint, rigid-exoskeleton, study-observational, setting-inpatient, outcome-FIM, stage-acute, actuation-motorized
Biomechanical Reactions of Exoskeleton Neurorehabilitation Robots in Spastic Elbows and Wrists [157]	2017	sensors, actuators, control, wrist, elbow, arm-general, multi-joint, rigid-exoskeleton, study-observational, force-torque-sensing, setting-lab, outcome-MAS, stage-chronic, actuation-motorized
Recovery of proprioception in the upper extremity by robotic mirror therapy: A clinical pilot study for proof of concept [158]	2017	sensors, actuators, control, wrist, elbow, arm-general, multi-joint, rigid-exoskeleton, study-case, IMU-kine, setting-outpatient, outcome-FMA-UE, outcome-Barthel, outcome-MAS, stage-chronic, actuation-motorized
Effects of robot-assisted upper limb rehabilitation in stroke patients: a systematic review with meta-analysis [159]	2017	arm-general, rigid-exoskeleton, study-review, outcome-FMA-UE, outcome-MAL, outcome-FIM, outcome-MAS, stage-subacute, stage-chronic
Design of a clinically relevant upper-limb exoskeleton robot for stroke patients with spasticity [160]	2017	sensors, actuators, control, hand, wrist, finger, elbow, arm-general, multi-joint, rigid-exoskeleton, study-pilot-feasibility, force-torque-sensing, setting-inpatient, outcome-MAS, actuation-motorized
The combined action of a passive exoskeleton and an EMG-controlled neuroprosthesis for upper limb stroke rehabilitation: First results of the RETRAINER project [161]	2017	sensors, actuators, control, elbow, shoulder, arm-general, multi-joint, rigid-exoskeleton, study-pilot-feasibility, EMG-interface, setting-inpatient, stage-subacute, stage-chronic
Improving robotic stroke rehabilitation by incorporating neural intent detection: Preliminary results from a clinical trial [162]	2017	sensors, actuators, control, AI, elbow, arm-general, rigid-exoskeleton, study-clinical-trial, study-pilot-feasibility, EMG-interface, EEG-BCI, setting-lab, outcome-FMA-UE, stage-chronic, actuation-motorized, AI-intent-decoding
Mechanical design of EFW Exo II: A hybrid exoskeleton for elbow-forearm-wrist rehabilitation [163]	2017	sensors, actuators, control, home_telerehab, wrist, elbow, arm-general, multi-joint, rigid-exoskeleton, hybrid, study-technical, force-torque-sensing, setting-lab, actuation-motorized

Title	Year	Labels
Design and kinematic analysis of a novel upper limb exoskeleton for rehabilitation of stroke patients [164]	2017	sensors, actuators, wrist, elbow, shoulder, arm-general, multi-joint, rigid-exoskeleton, study-technical, setting-lab, actuation-motorized
Feedforward model based arm weight compensation with the rehabilitation robot ARMin [165]	2017	sensors, actuators, control, elbow, shoulder, arm-general, multi-joint, rigid-exoskeleton, study-technical, EMG-interface, setting-lab, actuation-motorized
Strength training of the upper extremity in virtual reality with an exoskeleton robot [166]	2017	sensors, actuators, control, hand, elbow, shoulder, arm-general, multi-joint, rigid-exoskeleton, study-case, force-torque-sensing, setting-lab, outcome-FMA-UE, stage-chronic, actuation-motorized, control-impedance, control-adaptive
Improvement of upper extremity rehabilitation Robotic Exoskeleton, NREX [167]	2017	actuators, hand, wrist, elbow, shoulder, arm-general, multi-joint, rigid-exoskeleton, study-technical, actuation-motorized
Effects of assist-as-needed upper extremity robotic therapy after incomplete spinal cord injury: A parallel-group controlled trial [168]	2017	actuators, control, wrist, elbow, multi-joint, rigid-exoskeleton, study-clinical-trial, study-pilot-feasibility, setting-lab, outcome-ARAT, outcome-MAS, stage-chronic, actuation-cabled, actuation-motorized, control-adaptive
Modifying upper-limb inter-joint coordination in healthy subjects by training with a robotic exoskeleton [169]	2017	sensors, actuators, control, elbow, shoulder, arm-general, multi-joint, rigid-exoskeleton, study-technical, setting-lab, actuation-motorized, control-impedance
Sensory tractography and robot-quantified proprioception in hemiparetic children with perinatal stroke [170]	2017	sensors, actuators, elbow, arm-general, rigid-exoskeleton, study-observational, setting-lab, stage-chronic, actuation-motorized
Design and control of an upper arm exoskeleton using Fuzzy logic techniques [171]	2017	sensors, actuators, control, AI, elbow, arm-general, rigid-exoskeleton, study-technical, EMG-interface, force-torque-sensing, setting-lab, actuation-motorized, control-adaptive, control-PID, AI-intent-decoding
Adaptive iterative observer based on integral backstepping control for upper extremity exoskelton robot [172]	2017	sensors, actuators, control, wrist, elbow, shoulder, arm-general, multi-joint, rigid-exoskeleton, study-technical, force-torque-sensing, setting-lab, actuation-motorized, control-adaptive
Eye gaze tracking based interaction method of an upper-limb exoskeletal rehabilitation robot [173]	2017	sensors, actuators, control, elbow, shoulder, arm-general, multi-joint, rigid-exoskeleton, study-technical, vision-based, setting-lab, actuation-cabled, actuation-motorized
Use of an actuated glove to facilitate hand rehabilitation after stroke [174]	2017	sensors, actuators, control, hand, finger, glove, soft-exosuit, study-clinical-trial, study-pilot-feasibility, force-torque-sensing, outcome-FMA-UE, outcome-WMFT, stage-subacute, actuation-cabled, actuation-motorized
Upper extremity training with CUREs robot in subacute stroke: A pilot study [175]	2017	sensors, actuators, control, elbow, shoulder, arm-general, multi-joint, rigid-exoskeleton, study-clinical-trial, study-pilot-feasibility, force-torque-sensing, setting-inpatient, outcome-FMA-UE, outcome-MAS, stage-subacute, actuation-motorized, control-impedance
Design of a 3D printed lightweight orthotic device based on twisted and coiled polymer muscle: IGrab hand orthosis [176]	2017	sensors, actuators, hand, finger, multi-joint, rigid-exoskeleton, study-technical, vision-based, force-torque-sensing, setting-lab, actuation-cabled
Hand rehabilitation after stroke using a wearable, high DOF, spring powered exoskeleton [177]	2016	sensors, actuators, hand, finger, rigid-exoskeleton, study-pilot-feasibility, vision-based, force-torque-sensing, setting-lab, stage-chronic
A novel scheme of finger recovery based on symmetric rehabilitation: Specially for hemiplegia [178]	2016	sensors, actuators, control, AI, hand, finger, multi-joint, glove, rigid-exoskeleton, study-technical, EMG-interface, IMU-kine, setting-lab, actuation-motorized, AI-intent-decoding

Title	Year	Labels
Biomechtronic design of a wearable upper dual-arm exoskeleton robot for lifting tasks [179]	2016	sensors, actuators, control, elbow, shoulder, arm-general, multi-joint, rigid-exoskeleton, study-technical, setting-lab, actuation-motorized, control-PID
Design and Evaluation of the RUPERT Wearable Upper Extremity Exoskeleton Robot for Clinical and In-Home Therapies [180]	2016	sensors, actuators, control, home_telerehab, wrist, elbow, shoulder, arm-general, multi-joint, rigid-exoskeleton, study-pilot-feasibility, force-torque-sensing, setting-home, setting-lab, outcome-FMA-UE, outcome-WMFT, outcome-MAL, stage-chronic, actuation-PAM, control-adaptive, control-PID
Bilateral robots for upper-limb stroke rehabilitation: State of the art and future prospects [181]	2016	sensors, actuators, control, home_telerehab, wrist, elbow, shoulder, arm-general, multi-joint, rigid-exoskeleton, study-review, force-torque-sensing, actuation-motorized, control-admittance, control-adaptive
Instrumental indices for upper limb function assessment in stroke patients: A validation study [182]	2016	sensors, elbow, shoulder, arm-general, multi-joint, rigid-exoskeleton, study-observational, IMU-kine, setting-inpatient, outcome-WMFT, stage-subacute
Exoskeleton robots for upper-limb rehabilitation [183]	2016	sensors, actuators, control, home_telerehab, elbow, arm-general, multi-joint, rigid-exoskeleton, study-technical, vision-based, setting-lab, actuation-motorized
An exploratory investigation on the use of closed-loop electrical stimulation to assist individuals with stroke to perform fine movements with their hemiparetic arm [184]	2016	sensors, actuators, control, wrist, elbow, shoulder, arm-general, multi-joint, study-pilot-feasibility, IMU-kine, setting-lab, outcome-FMA-UE, stage-chronic
Evaluation of the effects of the Arm Light Exoskeleton on movement execution and muscle activities: A pilot study on healthy subjects [185]	2016	sensors, actuators, control, elbow, shoulder, arm-general, multi-joint, rigid-exoskeleton, study-pilot-feasibility, setting-lab, actuation-cabled, actuation-motorized, control-impedance
Tailor-made rehabilitation approach using multiple types of hybrid assistive limb robots for acute stroke patients: A pilot study [186]	2016	sensors, actuators, control, elbow, rigid-exoskeleton, study-pilot-feasibility, EMG-interface, setting-inpatient, outcome-FIM, outcome-Barthel, stage-acute, actuation-motorized
Development of a novel shoulder exoskeleton using parallel actuation and slip [187]	2016	sensors, actuators, control, shoulder, rigid-exoskeleton, study-technical, vision-based, setting-lab, actuation-motorized, control-PID
Upper-limb robotic exoskeletons for neurorehabilitation: A review on control strategies [188]	2016	sensors, actuators, control, wrist, elbow, shoulder, arm-general, multi-joint, rigid-exoskeleton, study-review
A new treatment in the rehabilitation of the paretic upper limb after stroke: The ARAMIS prototype and treatment protocol [189]	2016	sensors, actuators, control, wrist, elbow, shoulder, arm-general, multi-joint, rigid-exoskeleton, study-observational, setting-inpatient, outcome-FMA-UE, outcome-FIM, stage-subacute, actuation-motorized, control-adaptive
Modeling and design of a tendon actuated soft robotic exoskeleton for hemiparetic upper limb rehabilitation [190]	2015	actuators, hand, wrist, finger, elbow, arm-general, multi-joint, glove, soft-exosuit, study-technical, setting-lab, actuation-cabled, actuation-motorized
Adaptive wrist robot training in pediatric rehabilitation [191]	2015	sensors, actuators, control, wrist, rigid-exoskeleton, study-case, IMU-kine, setting-outpatient, outcome-BBT, outcome-MAS, stage-chronic, actuation-motorized, control-impedance, control-adaptive
User requirements for assistance of the supporting hand in bimanual daily activities via a robotic glove for severely affected stroke patients [192]	2015	hand, wrist, finger, glove, study-observational, stage-subacute
Efficacy of torque versus myocontrol for active, robotic-assisted rehabilitation of the shoulder after stroke: An experimental study [193]	2015	sensors, actuators, control, shoulder, arm-general, rigid-exoskeleton, study-pilot-feasibility, EMG-interface, force-torque-sensing, setting-inpatient, outcome-FMA-UE, stage-subacute, actuation-PAM

Title	Year	Labels
Robotic exoskeletons: a perspective for the rehabilitation of arm coordination in stroke patients [194]	2014	sensors, actuators, control, wrist, elbow, shoulder, arm-general, multi-joint, rigid-exoskeleton, study-review, EMG-interface, EEG-BCI, IMU-kine, outcome-FMA-UE, stage-acute, stage-chronic, actuation-hydraulic, actuation-cabled, actuation-motorized, control-impedance, control-admittance, control-adaptive
Design and Development of the Cable Actuated Finger Exoskeleton for Hand Rehabilitation Following Stroke [195]	2014	sensors, actuators, control, hand, finger, multi-joint, rigid-exoskeleton, study-technical, force-torque-sensing, setting-lab, actuation-cabled, actuation-motorized, control-PID
A task-oriented brain-computer interface rehabilitation system for patients with stroke hemiplegia [196]	2014	sensors, actuators, control, hand, wrist, finger, rigid-exoskeleton, study-pilot-feasibility, study-case, EEG-BCI, stage-chronic, actuation-motorized, AI-intent-decoding
Towards a brain computer interface driven exoskeleton for upper extremity rehabilitation [197]	2014	sensors, actuators, control, AI, wrist, elbow, arm-general, multi-joint, rigid-exoskeleton, study-pilot-feasibility, EEG-BCI, setting-lab, actuation-motorized, AI-intent-decoding
Design of the pediatric arm rehabilitation robot ChARMin [198]	2014	sensors, actuators, control, elbow, shoulder, arm-general, multi-joint, rigid-exoskeleton, study-technical, force-torque-sensing, setting-lab, actuation-motorized
Three-dimensional, task-specific robot therapy of the arm after stroke: A multicentre, parallel-group randomised trial [199]	2014	sensors, actuators, control, hand, elbow, shoulder, arm-general, multi-joint, rigid-exoskeleton, study-RCT, study-clinical-trial, outcome-FMA-UE, outcome-WMFT, outcome-MAL, outcome-MAS, stage-chronic, actuation-motorized, control-adaptive
Low cost and light-weight multi-DOF exoskeleton for comprehensive upper limb rehabilitation [200]	2014	sensors, actuators, hand, wrist, finger, shoulder, arm-general, multi-joint, soft-exosuit, rigid-exoskeleton, hybrid, study-technical, vision-based, setting-lab, actuation-cabled, actuation-motorized
System characterization of MAHI Exo-II: A robotic exoskeleton for upper extremity rehabilitation [201]	2014	sensors, actuators, control, wrist, elbow, shoulder, arm-general, multi-joint, rigid-exoskeleton, study-technical, setting-lab, actuation-cabled, actuation-motorized, control-PID
Design and development of wearable upper extremities powered exoskeleton - A cost-effective rehabilitation system [202]	2014	sensors, actuators, control, hand, wrist, elbow, shoulder, arm-general, multi-joint, rigid-exoskeleton, study-technical, force-torque-sensing, setting-lab, actuation-PAM
Clinical effects of using HEXORR (Hand Exoskeleton Rehabilitation Robot) for movement therapy in stroke rehabilitation [203]	2013	sensors, actuators, control, hand, finger, multi-joint, rigid-exoskeleton, study-pilot-feasibility, force-torque-sensing, setting-lab, outcome-FMA-UE, outcome-ARAT, outcome-BBT, outcome-MAS, stage-chronic, actuation-motorized, control-adaptive
Towards a parameterizable exoskeleton for training of hand function after stroke [204]	2013	sensors, actuators, control, hand, finger, multi-joint, rigid-exoskeleton, study-technical, setting-lab, actuation-cabled, actuation-motorized
Design of a self-aligning 3-DOF actuated exoskeleton for diagnosis and training of wrist and forearm after stroke [205]	2013	sensors, actuators, control, wrist, arm-general, multi-joint, rigid-exoskeleton, study-technical, force-torque-sensing, setting-lab, actuation-motorized
A crossover pilot study evaluating the functional outcomes of two different types of robotic movement training in chronic stroke survivors using the arm exoskeleton BONES [206]	2013	sensors, actuators, control, hand, wrist, elbow, shoulder, arm-general, multi-joint, rigid-exoskeleton, study-clinical-trial, study-pilot-feasibility, force-torque-sensing, setting-lab, outcome-FMA-UE, outcome-WMFT, outcome-BBT, outcome-MAL, outcome-MAS, stage-chronic, control-adaptive
Design of Wrist Gimbal: a forearm and wrist exoskeleton for stroke rehabilitation [207]	2013	sensors, actuators, control, wrist, arm-general, multi-joint, rigid-exoskeleton, study-technical, setting-lab, actuation-cabled, actuation-motorized, control-PID

Title	Year	Labels
Fine finger motor skill training with exoskeleton robotic hand in chronic stroke: Stroke rehabilitation [208]	2013	sensors, actuators, control, hand, finger, rigid-exoskeleton, study-case, EMG-interface, force-torque-sensing, setting-lab, outcome-FMA-UE, outcome-ARAT, outcome-FIM, stage-chronic, actuation-motorized
NREX: NRC Robotic Exoskeleton [209]	2013	sensors, actuators, control, wrist, elbow, shoulder, arm-general, multi-joint, rigid-exoskeleton, study-technical, force-torque-sensing, setting-lab, actuation-motorized
Positive effects of robotic exoskeleton training of upper limb reaching movements after stroke [210]	2012	sensors, actuators, control, wrist, elbow, shoulder, arm-general, multi-joint, rigid-exoskeleton, study-clinical-trial, EMG-interface, setting-lab, outcome-FMA-UE, outcome-MAS, stage-chronic, actuation-motorized, control-impedance
Development and control of a robotic exoskeleton for shoulder, elbow and forearm movement assistance [211]	2012	sensors, actuators, control, elbow, shoulder, arm-general, multi-joint, rigid-exoskeleton, study-technical, setting-lab, actuation-motorized
Design and implementation of a training strategy in chronic stroke with an arm robotic exoskeleton [212]	2011	sensors, actuators, control, elbow, shoulder, arm-general, multi-joint, rigid-exoskeleton, study-case, study-technical, force-torque-sensing, outcome-FMA-UE, outcome-MAS, stage-chronic, actuation-motorized, control-impedance, control-adaptive
An EMG-driven exoskeleton hand robotic training device on chronic stroke subjects: task training system for stroke rehabilitation [213]	2011	sensors, actuators, control, home_telerehab, hand, finger, arm-general, multi-joint, rigid-exoskeleton, study-clinical-trial, study-pilot-feasibility, EMG-interface, setting-lab, outcome-FMA-UE, outcome-ARAT, stage-chronic, actuation-motorized
Hand Spring Operated Movement Enhancer (HandSOME): a portable, passive hand exoskeleton for stroke rehabilitation [214]	2011	sensors, actuators, home_telerehab, hand, finger, arm-general, rigid-exoskeleton, study-clinical-trial, study-pilot-feasibility, study-case, EMG-interface, force-torque-sensing, setting-lab, outcome-MAS, actuation-SEA
Changing human upper-limb synergies with an exoskeleton using viscous fields [215]	2011	sensors, actuators, control, elbow, shoulder, arm-general, multi-joint, rigid-exoskeleton, study-technical, vision-based, force-torque-sensing, setting-lab, actuation-motorized, control-impedance
Mechanical design of a distal arm exoskeleton for stroke and spinal cord injury rehabilitation [216]	2011	sensors, actuators, wrist, elbow, shoulder, arm-general, multi-joint, rigid-exoskeleton, study-technical, actuation-cabled, actuation-motorized
Gravity compensation of an upper extremity exoskeleton [217]	2010	sensors, actuators, control, elbow, shoulder, arm-general, multi-joint, rigid-exoskeleton, study-technical, force-torque-sensing, setting-lab, actuation-motorized
Cooperative and active assistance based interactive therapy [218]	2010	sensors, actuators, control, hand, wrist, elbow, shoulder, arm-general, multi-joint, rigid-exoskeleton, study-clinical-trial, study-pilot-feasibility, setting-lab, outcome-FMA-UE, outcome-WMFT, outcome-MAL, stage-chronic, actuation-PAM, control-adaptive, control-PID
Design of a rotational hydroelastic actuator for a powered exoskeleton for upper limb rehabilitation [219]	2010	sensors, actuators, control, elbow, shoulder, arm-general, multi-joint, rigid-exoskeleton, study-technical, force-torque-sensing, setting-lab, actuation-SEA, actuation-hydraulic, control-impedance, control-admittance, control-PID

Label Taxonomy Glossary

This glossary provides a one- to two-line operational definition for every label used in the taxonomy of Figure 2 and applied to the 219 included studies (Table S2, Supplementary Material). Labels are grouped by the two analytical branches of the taxonomy: Technical and Biomedical. Multiple labels could be assigned to the same study; a label was applied only when the corresponding feature was explicitly reported (Section 2.4).

Technical branch

Anatomical target

Label	Operational definition
Hand	Device or intervention targets the hand as a whole (multiple digits and/or palm).
Finger	Device or intervention targets individual finger flexion/extension.
Wrist	Device targets wrist flexion/extension or radial-ulnar deviation.
Elbow	Device targets elbow flexion/extension.
Shoulder	Device targets shoulder abduction, flexion, or rotation.
Arm-general	Device spans or targets the arm without segment-specific distinction, or the whole upper limb.

Structure

Label	Operational definition
Rigid-exoskeleton	Rigid or semi-rigid mechanical linkage configured in parallel with the limb; meets the ISO 8373 two-actuator robotic-device criterion.
Soft-exosuit	Compliant textile/fabric-based wearable transmitting force through tendons, cables, or pneumatic elements rather than rigid links.
Glove	Hand-specific wearable device (typically fabric- or silicone-based) actuating finger flexion and/or extension.
Hybrid	Device combining a robotic exoskeleton or exosuit with functional electrical stimulation (FES).
Multi-joint	Device spans and actuates more than one joint (e.g., shoulder-elbow-wrist).

Actuators

Label	Operational definition
Actuation-motorized	Electric motor-driven actuation (DC or servo motors); the most common actuation approach in the reviewed literature.
Hydraulic	Fluid-pressure-driven actuation; higher force density, less common in wearable devices.
Cable	Tendon/cable transmission routing force from a remote actuator to the joint.
PAM	Pneumatic Artificial Muscle: compressed-air-driven compliant actuator.
SEA	Series Elastic Actuator: motor with an inline elastic element enabling passive compliance and force control.
SMA	Shape Memory Alloy actuator: thermally activated compliant element, mainly used in soft gloves.
VSA	Variable Stiffness Actuator: mechanism allowing online tuning of joint compliance.

Sensors

Label	Operational definition
IMU (IMU-kine)	Inertial Measurement Unit (accelerometer/gyroscope/magnetometer) used for joint/segment angle estimation and motion tracking.
EMG (EMG-interface)	Surface electromyography sensing muscular activation to decode voluntary motor intent.
EEG-BCI	Electroencephalography-based brain-computer interface decoding cortical activity (e.g., motor imagery, SSVEP).
Force/torque (force-torque-sensing)	Sensors measuring interaction forces or torques at the human-robot interface (wrench, joint-torque, or pressure sensors).
Vision-based	Camera or optical systems (RGB-D, marker-based motion capture) used for kinematic tracking or environmental perception.

Control

Label	Operational definition
Control-PID	Proportional-Integral-Derivative feedback control of position, velocity, or torque.
Control-impedance	Control strategy regulating the dynamic relationship between position/velocity and interaction force.
Control-admittance	Control strategy computing a motion response from measured interaction force.
MPC	Model Predictive Control: control using a predictive model to optimise future actions over a receding horizon.
Control-RL	Reinforcement-learning-based control policy learned through reward-driven interaction.
Control-adaptive	Control strategy that adjusts parameters online to subject- or task-specific variability (e.g., assist-as-needed).

Enabling technologies

Label	Operational definition
AI	Artificial intelligence methods (machine learning/deep learning) applied to control, decoding, or outcome assessment.
Home_telerehab	Telerehabilitation or home-based delivery with remote monitoring capability.
AI-intent-decoding	AI-based decoding of the user's voluntary movement intent from biological or interaction signals.

Biomedical branch

Outcome

Label	Operational definition
FMA-UE	Fugl-Meyer Assessment for the Upper Extremity: impairment-level motor-function scale.
ARAT	Action Research Arm Test: activity-level test of grasp, grip, pinch, and gross arm movement.
MAS	Modified Ashworth Scale: clinical assessment of spasticity/muscle tone.
BBT	Box and Block Test: gross manual dexterity test (blocks transferred per 60 seconds).
MAL	Motor Activity Log: patient-reported outcome of real-world affected-arm use.
Barthel	Barthel Index: scale of basic activities-of-daily-living independence.
FIM	Functional Independence Measure: broader scale of functional independence.
WMFT	Wolf Motor Function Test: timed and quality-rated assessment of upper-limb task performance.

Stage

Label	Operational definition
Stage-acute	Within the first days after stroke onset (typically <1 month).
Stage-subacute	Approximately 1–6 months post-stroke, the period of peak spontaneous neurological recovery.
Stage-chronic	More than 6 months post-stroke, a period of relatively stable impairment.

Setting

Label	Operational definition
Setting-lab	Controlled laboratory or technical validation environment, not a clinical care setting.
Setting-inpatient	Hospital or inpatient rehabilitation ward.
Setting-outpatient	Outpatient clinic or rehabilitation centre.
Setting-home	Home-based use, unsupervised or remotely supervised via telerehabilitation.

Study type

Label	Operational definition
Study-RCT	Randomised controlled trial with random allocation to intervention/control conditions.
Study-clinical-trial	Controlled or uncontrolled clinical trial without randomisation.
Study-observational	Non-interventional observational study (e.g., cohort, cross-sectional).
Study-case	Case study or case series (single patient or small case group), descriptive in nature.
Study-protocol	Published study protocol without reported outcome data.
Study-review	Previous systematic or narrative review or meta-analysis, cited as background evidence.
Study-technical	Engineering or technical validation study, conducted with healthy subjects or bench-level testing.
Study-pilot-feasibility	Pilot or feasibility study; hypothesis-generating and not powered for efficacy testing.

References

1. Zheng, S.; Meng, N. Recovr Glove: Accessible Hand Exoskeleton for Stroke Rehabilitation and Everyday Aid. *IEEE Int Conf Rehabil Robot* **2025**. https://doi.org/10.1109/ICORR66766.2025.11062937.
2. Phan, T.Q.; Alghamdi, M.; Egwim, M.; Lee, S.W. Development and Validation of Customizable, Adaptive SPring-Actuated Hand EXoskeleton (CASPHEX) Designed for Home-Based Training of Finger Movements Post-Stroke. *IEEE Trans Neural Syst Rehabil Eng* **2025**. https://doi.org/10.1109/TNSRE.2025.3615078.
3. Tanczak, N.; Plunkett, T.; Lin, S.; Kuenzler, L.; Lau, M.; Kuah, W.; Ng, C.; Gassert, R.; Chua, K.; Lambercy, O. Feasibility of post-stroke hand rehabilitation supported by a soft robotic hand orthosis in-clinic and at-home. *Journal of NeuroEngineering and Rehabilitation* **2025**, *22*. https://doi.org/10.1186/s12984-025-01717-6.
4. Ji, X.; Lu, X.; Xu, Y.; Zhang, W.; Yang, H.; Yin, C.; Wang, H.; Ren, C.; Ji, Y.; Li, Y.; et al. Effects and neural mechanisms of a brain-computer interface-controlled soft robotic glove on upper limb function in patients with subacute stroke: a randomized controlled fNIRS study. *Journal of NeuroEngineering and Rehabilitation* **2025**, *22*. https://doi.org/10.1186/s12984-025-01704-x.
5. Drogos, J.; Carmona, C.; Arceo, R.; Yao, J. Effects of device-assisted practice of activities of daily living in a close-to-normal pattern on upper extremity motor recovery in individuals with moderate to severe stroke: study protocol of a randomized control trial. *Trials* **2025**, *26*. https://doi.org/10.1186/s13063-025-08930-7.
6. Zhang, R.; Li, Z.; Pan, X.; Cui, H.; Chen, X. Hybrid BCI for upper limb rehabilitation: integrating MI with peripheral field SSVEP stimulation. *Journal of Neuroscience Methods* **2025**, *423*. https://doi.org/10.1016/j.jneumeth.2025.110537.
7. Chen, J.; Cheng, Z.; Cai, Y.; Zhang, S.; Zhu, C.; Zhang, Y. Robot-Assisted Mirror Rehabilitation for Post-Stroke Upper Limbs: A Personalized Control Strategy. *Sensors* **2025**, *25*. https://doi.org/10.3390/s25185659.
8. Koh, K.; Oppizzi, G.; Baghi, R.; Kehs, G.; Zhang, L.Q. Loss of Joint Individuation and Abnormal Synergy Post Stroke in Upper Limb Movements. *Neurorehabilitation and Neural Repair* **2025**, *39*. https://doi.org/10.1177/15459683251340914.
9. Zhang, L.; Tian, X.; Fan, Y.; Jiang, T.; Gu, S.; Xu, L. Development and Efficacy Assessment of an Angle Sensor-Integrated Upper Limb Exoskeleton System for Autonomous Rehabilitation Training. *Sensors* **2025**, *25*. https://doi.org/10.3390/s25133984.
10. Shakeriaski, F.; Mohammadian, M. Enhancing Upper Limb Exoskeletons Using Sensor-Based Deep Learning Torque Prediction and PID Control. *Sensors* **2025**, *25*. https://doi.org/10.3390/s25113528.
11. Zhao, Y.; Mao, J.; Zhang, M.; Wu, H.; Jiang, J.; Jing, S. Integration of neuromuscular control for multidirectional horizontal planar reaching movements in a portable upper limb exoskeleton for enhanced stroke rehabilitation. *Biomedizinische Technik* **2025**, *70*. https://doi.org/10.1515/bmt-2023-0622.
12. Nishimura, M.; Kobayashi, S.; Kuninaka, T.; Hokama, Y.; Nagamine, H.; Ishiuchi, S. Rehabilitation with hybrid assistive limb improves upper limb paralysis in patients with cerebral hemorrhage by repairing axonal injury of the corticospinal tract. *Journal of Neural Engineering* **2025**, *22*. https://doi.org/10.1088/1741-2552/adbe3c.
13. Pei, S.; Wang, J.; Tian, C.; Li, X.; Guo, B.; Guo, J.; Yao, Y. Assist-as-Needed Controller of a Rehabilitation Exoskeleton for Upper-Limb Natural Movements. *Applied Sciences (Switzerland)* **2025**, *15*. https://doi.org/10.3390/app15052644.
14. Yeh, S.J.; Wang, Y.C.; Fang, W.C.; Huang, S.C.; Yang, Y.S. Effectiveness of Powered Hand Exoskeleton on Upper Extremity Function in People with Chronic Stroke. *Actuators* **2025**, *14*. https://doi.org/10.3390/act14020067.

15. Wang, C.; Peng, L.; Hou, Z.G. Development and Control of an Upper-Limb Exoskeleton CASIA-EXO for Motor Learning in Post-Stroke Rehabilitation. *IEEE/CAA Journal of Automatica Sinica* **2025**, *12*. https://doi.org/10.1109/JAS.2024.124662.
16. He, Z.; Liu, Y.; Bian, Y.; Jiang, M.; Yi, Z.; Xiao, Z.; Ma, Y.; Lui, Z.; Wang, L.; Wang, K. Design of a Variable-Stiffness Exoskeleton for Upper Limb Rehabilitation. 2025. https://doi.org/10.1109/ICMA65362.2025.11120656.
17. Abdelraouf, O.; Abdel Ghafar, M.; Mohamed, M.; Ibrahim, Z.; Harraz, E.; Seyam, M.; Mousa, G.; Radwan, R.; El-Bagalaty, A. SOFT ROBOTIC GLOVES VERSUS MIRROR THERAPY: A LONG-TERM COMPARATIVE STUDY ON HAND FUNCTION AND MOTOR RECOVERY IN POST-STROKE REHABILITATION. *Journal of Rehabilitation Medicine* **2025**, *57*. https://doi.org/10.2340/jrm.v57.43482.
18. Proulx, C.; Higgins, J.; Vaughan, T.; Hewko, M.; Gagnon, D. Poststroke Neurorehabilitation Using a Soft Robotic Glove Combined With a Virtual Environment: Preliminary Study on Feasibility, Safety, Effects, and User Satisfaction. *JMIR Neurotechnology* **2025**, *4*. https://doi.org/10.2196/69750.
19. Mayouf, T.; Al-Jubouri, M. THE EFFECT OF SOFT ROBOTIC GLOVE ON THE FLEXION AND EXTENSION OF HAND FOR STROKE PATIENTS: A CLINICAL TRIAL. *Georgian Medical News* **2025**, *361*.
20. Berdal, J.; Hartnett, R.; Quintero, D. Up-Care: An Upper Limb Portable Cable Assistive Recovery Exoskeleton for Elbow Movement Assistance. In Proceedings of the IEEE International Conference on Rehabilitation Robotics, 2025. https://doi.org/10.1109/ICORR66766.2025.11063208.
21. Ghozzi, R.; Ammar, A.; Hammouda, M.; Ali, J.; Lahouar, S.; Souani, C. Robotic Exoskeleton for Upper Arm Stroke Rehabilitation. 2025. https://doi.org/10.1109/ICCIT63348.2025.10989311.
22. Danaish, n.; Han, L.; Xu, G.; Gao, Y.; Baig, M.; Dong, G.; Xu, Z. A lightweight, fully wearable 7-DOF upper limb exoskeleton for home-based rehabilitation: design, modeling, and experimental validation. *International Journal of Intelligent Robotics and Applications* **2025**. https://doi.org/10.1007/s41315-025-00446-2.
23. Yu, J.; Luo, L.; Zhu, W.; Li, Y.; Xie, P.; Zhang, L. A Novel Low-Pressure Robotic Glove Based on CT-Optimized Finger Joint Kinematic Model for Long-Term Rehabilitation of Stroke Patients. *IEEE Trans Neural Syst Rehabil Eng* **2024**. https://doi.org/10.1109/TNSRE.2023.3337827.
24. Song, D.; Liu, L.; Zhu, T.; Zhang, S.; Huang, Y. B-FMEA-TRIZ model for scheme decision in conceptual product design: A study on upper-limb hemiplegia rehabilitation exoskeleton. *Heliyon* **2024**. https://doi.org/10.1016/j.heliyon.2024.e30684.
25. Vargas, M.; Vasquez, Y.; de la Barra, D.; Charapaqui, S.; Tapia-Yanayaco, P.; Maldonado-Gómez, R.; Mendoza-Arias, L.; Altatorre, A.; Ccellccaro, C.; Bedoya-Castillo, M.; et al. Elbow-Hand Robotic Exoskeletons for Active and Passive Rehabilitation on Post-Stroke Patients: A Bioengineering Review. *HighTech and Innovation Journal* **2024**, *5*. https://doi.org/10.28991/HIJ-2024-05-04-020.
26. Culp, S.; DiRocco, S.; Brunfeldt, A.; Casas, R.; Lum, P. Gravity support from a robotic exoskeleton increases spontaneous use of the nondominant upper extremity during a choice reaching task. *Journal of Neurophysiology* **2024**, *132*. https://doi.org/10.1152/jn.00261.2024.
27. Lo, Y.; Lim, M.; Kok, C.; Wang, S.; Blok, S.; Ang, T.; Ng, V.; Rao, J.; Chua, K. Neural Interface-Based Motor Neuroprosthesis in Poststroke Upper Limb Neurorehabilitation: An Individual Patient Data Meta-analysis. *Archives of Physical Medicine and Rehabilitation* **2024**, *105*. https://doi.org/10.1016/j.apmr.2024.04.001.
28. Höhler, C.; Trigili, E.; Astarita, D.; Hermsdörfer, J.; Jahn, K.; Krewer, C. The efficacy of hybrid neuroprostheses in the rehabilitation of upper limb impairment after stroke, a narrative and systematic review with a meta-analysis. *Artificial Organs* **2024**, *48*. https://doi.org/10.1111/aor.14618.
29. DiRocco, S.; Culp, S.; Casas, R.; Lum, P. Therapy for Abnormal Muscle Synergies in Stroke Using the ULIX Low-Impedance Robot. In Proceedings of the Annual International Conference of the IEEE Engineering in Medicine and Biology - Proceedings, 2024. https://doi.org/10.1109/EMBC53108.2024.10782051.
30. Chen, Z.; Ju, C.; Wu, J.; Tian, M.; Jia, F.; Wang, X. Kinematic and Dynamic Analysis of the Cable Actuation in an Upper-Extremity Exoskeleton. In Proceedings of the M2VIP - Proceedings of the International Conference on Mechatronics and Machine Vision in Practice, 2024, number 2024. https://doi.org/10.1109/M2VIP62491.2024.10746195.
31. Chen, Z.; Wu, J.; Ju, C.; Wang, X.; Tian, M.; Liu, B. Design and Control of a Cable-Driven Exoskeleton System for Upper-Extremity Rehabilitation. *IEEE Access* **2024**, *12*. https://doi.org/10.1109/ACCESS.2024.3515141.
32. Jin, X.; Ding, W.; Li, Q.; Yang, W.; Wei, Y. Mechanical Design and Analysis of Cable-Driven Wearable Flexible Exoskeleton System. In Proceedings of the Proceedings of SPIE - The International Society for Optical Engineering, 2024, Vol. 13285. https://doi.org/10.1117/12.3051998.
33. Privitera, L.; Lassi, M.; Dalise, S.; Azzolini, V.; Vercillo, F.; Maggiani, L.; Mazzoni, A.; Chisari, C.; Micera, S.; Bandini, A. Automatic Prediction of Upper Limb Motor Recovery in Stroke Patients Undergoing Robotic Therapy. In Proceedings of the Proceedings of the IEEE RAS and EMBS International Conference on Biomedical Robotics and Biomechatronics, 2024. https://doi.org/10.1109/BioRob60516.2024.10719781.
34. Hasan, S.; Bhujel, S.; Niemiec, G. Emerging trends in human upper extremity rehabilitation robot. *Cognitive Robotics* **2024**, 4. https://doi.org/10.1016/j.cogr.2024.09.001.

35. Mauro, M.; Fasano, A.; Germanotta, M.; Cortellini, L.; Insalaco, S.; Pavan, A.; Comanducci, A.; Guglielmelli, E.; Aprile, I. Restoring of Interhemispheric Symmetry in Patients With Stroke Following Bilateral or Unilateral Robot-Assisted Upper-Limb Rehabilitation: A Pilot Randomized Controlled Trial. *IEEE Transactions on Neural Systems and Rehabilitation Engineering* **2024**, *32*. https://doi.org/10.1109/TNSRE.2024.3460485.
36. Tang, Z.; Cui, Z.; Wang, H.; Liu, P.; Xu, X.; Yang, K. A 4-DOF Exosuit Using a Hybrid EEG-Based Control Approach for Upper-Limb Rehabilitation. *IEEE Journal of Translational Engineering in Health and Medicine* **2024**, *12*. https://doi.org/10.1109/JTEHM.2024.3454077.
37. Sankar, K.; Muthukumar, S.; Kannan, P.; Mariappan, S.; Kalidoss, S. Robot-Assisted Therapies for Upper Limb Stroke Rehabilitation: A Narrative Review. 2024. https://doi.org/10.1109/ICC-ROBINS60238.2024.10533977.
38. Kwok, T.; Yu, H. A Novel Bilateral Underactuated Upper Limb Exoskeleton for Post-Stroke Bimanual ADL Training. *IEEE Transactions on Neural Systems and Rehabilitation Engineering* **2024**, *32*. https://doi.org/10.1109/TNSRE.2024.3407653.
39. Prashanna Rangan, R.; Johnson, J.; Ramesh Babu, S.; Maheswari, C.; Vaisali, S.; Shankar, S. Design and Modelling of a Human Upper Limb for Rehabilitation Exoskeleton. *Journal of Engineering Science and Technology Review* **2024**, *17*. https://doi.org/10.25103/jestr.172.02.
40. Tseng, K.; Wang, L.; Hsieh, C.; Wong, A. Portable robots for upper-limb rehabilitation after stroke: a systematic review and meta-analysis. *Annals of Medicine* **2024**, *56*. https://doi.org/10.1080/07853890.2024.2337735.
41. Wang, J.; Pei, S.; Guo, J.; Dong, A.; Liu, B.; Yao, Y. An Upper Limb Exoskeleton Motion Generation Algorithm Based on Separating Shoulder and Arm Motion. *IEEE Transactions on Neural Systems and Rehabilitation Engineering* **2024**, *32*. https://doi.org/10.1109/TNSRE.2024.3356724.
42. Hasan, A.; Alhamri, M.; Firoz, O.; Khan, S.; Khan, S. Design and Control of 2DoF Wearable Robot Arm for Rehabilitation. 2024. https://doi.org/10.1109/ICRAI62391.2024.10894684.
43. Alhamad, R.; Seth, N.; Abdullah, H.A. Initial Testing of Robotic Exoskeleton Hand Device for Stroke Rehabilitation. *Sensors (Basel)* **2023**. https://doi.org/10.3390/s23146339.
44. Proulx, C.E.; Higgins, J.; Gagnon, D.H. Occupational therapists' evaluation of the perceived usability and utility of wearable soft robotic exoskeleton gloves for hand function rehabilitation following a stroke. *Disabil Rehabil Assist Technol* **2023**. https://doi.org/10.1080/17483107.2021.1938710.
45. Jo, S.; Song, Y.; Lee, Y.; Heo, S.H.; Jang, S.J.; Kim, Y.; Shin, J.H.; Jeong, J.; Park, H.S. Functional MRI Assessment of Brain Activity During Hand Rehabilitation with an MR-Compatible Soft Glove in Chronic Stroke Patients: A Preliminary Study. *IEEE Int Conf Rehabil Robot* **2023**. https://doi.org/10.1109/ICORR58425.2023.10304776.
46. Barria, P.; Riquelme, M.; Reppich, H.; Cisnal, A.; Fraile, J.C.; Pérez-Turiel, J.; Sierra, D.; Aguilar, R.; Andrade, A.; Nuñez Espinosa, C. Hand rehabilitation based on the RobHand exoskeleton in stroke patients: A case series study. *Front Robot AI* **2023**. https://doi.org/10.3389/frobt.2023.1146018.
47. Proulx, C.E.; Higgins, J.; Vincent, C.; Vaughan, T.; Hewko, M.; Gagnon, D.H. User-centered development process of an operating interface to couple a robotic glove with a virtual environment to optimize hand rehabilitation following a stroke. *J Rehabil Assist Technol Eng* **2023**. https://doi.org/10.1177/20556683231166574.
48. Lim, D.L.; Lai, H.S.; Yeow, R.H. A bidirectional fabric-based soft robotic glove for hand function assistance in patients with chronic stroke. *Journal of NeuroEngineering and Rehabilitation* **2023**, *20*. https://doi.org/10.1186/s12984-023-01250-4.
49. Austin, D.; Dixon, M.; Tulimieri, D.; Cashaback, J.; Semrau, J. Validating the measurement of upper limb sensorimotor behavior utilizing a tablet in neurologically intact controls and individuals with chronic stroke. *Journal of NeuroEngineering and Rehabilitation* **2023**, *20*. https://doi.org/10.1186/s12984-023-01240-6.
50. Khantan, M.; Avery, M.; Aung, P.; Zarin, R.; Hammelef, E.; Shawki, N.; Serruya, M.; Napoli, A. The NuroSleeve, a user-centered 3D printed hybrid orthosis for individuals with upper extremity impairment. *Journal of NeuroEngineering and Rehabilitation* **2023**, *20*. https://doi.org/10.1186/s12984-023-01228-2.
51. Ko, M.J.; Chuang, Y.C.; Ou-Yang, L.J.; Cheng, Y.Y.; Tsai, Y.L.; Lee, Y.C. The Application of Soft Robotic Gloves in Stroke Patients: A Systematic Review and Meta-Analysis of Randomized Controlled Trials. *Brain Sciences* **2023**, *13*. https://doi.org/10.3390/brainsci13060900.
52. Dobkin, B.; Busza, A. Upper Extremity Robotic-Assisted Rehabilitation: Results Not Yet Robust. *Stroke* **2023**, *54*. https://doi.org/10.1161/STROKEAHA.123.042570.
53. Chen, Z.J.; He, C.; Xu, J.; Zheng, C.J.; Wu, J.; Xia, N.; Hua, Q.; Xia, W.G.; Xiong, C.H.; Huang, X.L. Exoskeleton-Assisted Anthropomorphic Movement Training for the Upper Limb After Stroke: The EAMT Randomized Trial. *Stroke* **2023**, *54*. https://doi.org/10.1161/STROKEAHA.122.041480.
54. Pournajaf, S.; Giovanni, G.; Straudi, S.; Goffredo, M.; Leo, M.; Calabrò, R.; Felzani, G.; Paolucci, S.; Filoni, S.; Santamato, A.; et al. Neurophysiological and Clinical Effects of Upper Limb Robot-Assisted Rehabilitation on Motor Recovery in Patients with Subacute Stroke: A Multicenter Randomized Controlled Trial Study Protocol. *Brain Sciences* **2023**, *13*. https://doi.org/10.3390/brainsci13040700.

55. Greco, C.; Weerakkody, T.; Cichella, V.; Pagnotta, L.; Lamuta, C. Lightweight Bioinspired Exoskeleton for Wrist Rehabilitation Powered by Twisted and Coiled Artificial Muscles. *Robotics* **2023**, *12*. https://doi.org/10.3390/robotics12010027.
56. Wang, T.; Liu, Z.; Gu, J.; Tan, J.; Hu, T. Effectiveness of soft robotic glove versus repetitive transcranial magnetic stimulation in post-stroke patients with severe upper limb dysfunction: A randomised controlled trial. *Frontiers in Neurology* **2023**, *13*. https://doi.org/10.3389/fneur.2022.887205.
57. Ruiz-Sáez, P.; Velásquez-Oberreuter, L.; Torres Zúñiga, N.; Lapierre, M.; Castillo-González, W. Implementation of technological devices used by occupational therapists in upper extremity rehabilitation after a stroke; Implementación de dispositivos tecnológicos usados por terapeutas ocupacionales en la rehabilitación de extremidad superior posterior a un accidente cerebro vascular. *Salud, Ciencia y Tecnologia* **2023**, *3*. https://doi.org/10.56294/saludcyt2023694.
58. Pan, J.; Astarita, D.; Baldoni, A.; Dell'Agnello, F.; Crea, S.; Vitiello, N.; Trigili, E. A Self-Aligning Upper-Limb Exoskeleton Preserving Natural Shoulder Movements: Kinematic Compatibility Analysis. *IEEE Transactions on Neural Systems and Rehabilitation Engineering* **2023**, *31*. https://doi.org/10.1109/TNSRE.2023.3341219.
59. Lopez, D.; Thomson, C.; Mino, F.; Edgely, S.; Maitre, P.; Iversen, M.; George, J. Delayed Muscle Activity in Stroke Survivors with Upper-Limb Hemiparesis. In Proceedings of the Annual International Conference of the IEEE Engineering in Medicine and Biology - Proceedings, 2023. https://doi.org/10.1109/EMBC40787.2023.10340726.
60. Tang, Z.; Wang, H.; Cui, Z.; Jin, X.; Zhang, L.; Peng, Y.; Xing, B. An Upper-Limb Rehabilitation Exoskeleton System Controlled by MI Recognition Model With Deep Emphasized Informative Features in a VR Scene. *IEEE Transactions on Neural Systems and Rehabilitation Engineering* **2023**, *31*. https://doi.org/10.1109/TNSRE.2023.3329059.
61. Ratschat, A.; Lomba, T.; Dalla Gasperina, S.; Marchal-Crespo, L. Development and Validation of a Kinematically Accurate Upper-Limb Exoskeleton Digital Twin for Stroke Rehabilitation. In Proceedings of the IEEE International Conference on Rehabilitation Robotics, 2023. https://doi.org/10.1109/ICORR58425.2023.10304719.
62. Uehara, S.; Yuasa, A.; Ushizawa, K.; Kitamura, S.; Yamazaki, K.; Otaka, E.; Otaka, Y. Direction-dependent differences in the quality and quantity of horizontal reaching in people after stroke. *Journal of Neurophysiology* **2023**, *130*. https://doi.org/10.1152/jn.00455.2022.
63. Safira, L.; Arifin, A.; Kusuma, H.; Risciawan, A.; Zazuli, M. Adaptive PID Controller Based on Sliding Surface for Controlling Elbow Joint Robot Rehabilitation. 2023. https://doi.org/10.1109/ISITIA59021.2023.10221161.
64. Sidabalok, S.; Arifin, A.; Pramudijanto, J.; Kusuma, H.; Risciawan, A.; Zazuli, M. Development of Elbow Joint Exoskeleton Control System Using Fuzzy Pid Control Method for Post Stroke Rehabilitation. 2023. https://doi.org/10.1109/ISITIA59021.2023.10221019.
65. Zhang, X.; Wang, M.; Wang, H.; Wang, F.; Chen, L.; Mu, W.; Wang, J.; Kang, X. Design and Performance Analysis of a Bioelectronic Controlled Hybrid Serial-Parallel Wrist Exoskeleton. *IEEE Transactions on Neural Systems and Rehabilitation Engineering* **2023**, *31*. https://doi.org/10.1109/TNSRE.2023.3283603.
66. Yurkewich, A.; Ortega, S.; Sanchez, J.; Wang, R.H.; Burdet, E. Integrating hand exoskeletons into goal-oriented clinic and home stroke and spinal cord injury rehabilitation. *J Rehabil Assist Technol Eng* **2022**. https://doi.org/10.1177/20556683221130970.
67. Giovanni, G.; Capone, F.; Iosa, M.; Cruciani, A.; Paolucci, M.; Martino Cinnera, A.; Musumeci, G.; Brunelli, N.; Costa, C.; Paolucci, S.; et al. May Dual Transcranial Direct Current Stimulation Enhance the Efficacy of Robot-Assisted Therapy for Promoting Upper Limb Recovery in Chronic Stroke? *Neurorehabilitation and Neural Repair* **2022**, *36*. https://doi.org/10.1177/15459683221138743.
68. Frisoli, A.; Barsotti, M.; Sotgiu, E.; Lamola, G.; Procopio, C.; Chisari, C. A randomized clinical control study on the efficacy of three-dimensional upper limb robotic exoskeleton training in chronic stroke. *Journal of NeuroEngineering and Rehabilitation* **2022**, *19*. https://doi.org/10.1186/s12984-022-00991-y.
69. Delgado, P.; Rincon, C.; Yihun, Y. Human-Exoskeleton Joint Coordination Assessment: A Case Study on the Shoulder and Elbow Joints. *Journal of Bionic Engineering* **2022**, *19*. https://doi.org/10.1007/s42235-022-00226-9.

Weymann, S.; Pretty, C.; Pott, P. Development of an EMG-based Elbow-Exoskeleton with Twisted String Actuation. *Current Directions in Biomedical Engineering* **2022**, *8*. https://doi.org/10.1515/cdbme-2022-1141.

70. Georgarakis, A.M.; Zimmermann, Y.; Wolf, P.; Hutter, M.; Riener, R. Supporting and Stabilizing the Scapulohumeral Rhythm With a Body- or Robot-Powered Orthosis. *IEEE Transactions on Medical Robotics and Bionics* **2022**, *4*. https://doi.org/10.1109/TMRB.2022.3176728.
72. Kim, B.; Ahn, K.H.; Nam, S.; Hyun, D. Upper extremity exoskeleton system to generate customized therapy motions for stroke survivors. *Robotics and Autonomous Systems* **2022**, *154*. https://doi.org/10.1016/j.robot.2022.104128.
73. Chen, A.; Winterbottom, L.; Park, S.; Xu, J.; Nilsen, D.; Stein, J.; Ciocarlie, M. Thumb Stabilization and Assistance in a Robotic Hand Orthosis for Post-Stroke Hemiparesis. *IEEE Robotics and Automation Letters* **2022**, *7*. https://doi.org/10.1109/LRA.2022.3185365.
74. Cao, Q.; Li, J.; Dong, M. Comparative analysis of three categories of four-DOFs exoskeleton mechanism based on relative movement offsets. *Industrial Robot* **2022**, *49*. https://doi.org/10.1108/IR-12-2020-0273.
75. Ahmed, T.; Islam, M.; Brahim, B.; Rahman, M. Robustness and Tracking Performance Evaluation of PID Motion Control of 7 DoF Anthropomorphic Exoskeleton Robot Assisted Upper Limb Rehabilitation. *Sensors* **2022**, *22*. https://doi.org/10.3390/s22103747.

76. Tang, Z.; Heung, H.; Shi, X.; Tong, R.; Li, Z. Probabilistic Model-Based Learning Control of a Soft Pneumatic Glove for Hand Rehabilitation. *IEEE Transactions on Biomedical Engineering* **2022**, *69*. https://doi.org/10.1109/TBME.2021.3111891.
77. Nam, C.; Rong, W.; Li, W.; Cheung, C.; Ngai, W.; Cheung, T.; Pang, M.; Li, L.; Hu, J.; Wai, H.; et al. An Exoneuromusculoskeleton for Self-Help Upper Limb Rehabilitation after Stroke. *Soft Robotics* **2022**, *9*. https://doi.org/10.1089/soro.2020.0090.
78. Crema, A.; Bassolino, M.; Guanziroli, E.; Colombo, M.; Blanke, O.; Serino, A.; Micera, S.; Molteni, F. Neuromuscular electrical stimulation restores upper limb sensory-motor functions and body representations in chronic stroke survivors. *Med* **2022**, *3*. https://doi.org/10.1016/j.medj.2021.12.001.
79. Ramirez-Zamora, J.; Domínguez-Ramírez, O.; Sepúlveda-Cervantes, G.; Ramos-Velasco, L.; Fernandez-Ramirez, J. NASA-TLX Assessment for a Haptic Adaptive Platform for Upper Extremity Motor Rehabilitation. 2022. https://doi.org/10.1109/COMRob57154.2022.9962313.
80. Nithyaa, A.; Premkumar, R.; Manavalan, M.; Iswarya, C.; Lakshmi Priya, S. Exoskeleton Device for Left Hemiplegia Patients with Electromyograph. In Proceedings of the Journal of Physics: Conference Series, 2022, Vol. 2318. https://doi.org/10.1088/1742-6596/2318/1/012006.
81. Dalla Gasperina, S.; Longatelli, V.; Panzenbeck, M.; Luciani, B.; Morosini, A.; Piantoni, A.; Tropea, P.; Braghin, F.; Pedrocchi, A.; Gandolla, M. AGREE: an upper-limb robotic platform for personalized rehabilitation, concept and clinical study design. In Proceedings of the IEEE International Conference on Rehabilitation Robotics, 2022, Vol. 2022-July. https://doi.org/10.1109/ICORR55369.2022.9896569.
82. Hailey, R.; de Oliveira, A.; Ghonasgi, K.; Whitford, B.; Lee, R.; Rose, C.; Deshpande, A. Impact of Gravity Compensation on Upper Extremity Movements in Harmony Exoskeleton. In Proceedings of the IEEE International Conference on Rehabilitation Robotics, 2022, Vol. 2022-July. https://doi.org/10.1109/ICORR55369.2022.9896415.
83. Mahan, E.; Dunkelberger, N.; Oh, J.; Simmons, M.; Varghese, B.; Sayenko, D.; O-Malley, M. Measuring Torque Production with a Robotic Exoskeleton during Cervical Transcutaneous Spinal Stimulation. In Proceedings of the IEEE International Conference on Rehabilitation Robotics, 2022, Vol. 2022-July. https://doi.org/10.1109/ICORR55369.2022.9896477.
84. Jonna, P.; Rao, M. Design of a 6-DoF Cost-effective Differential-drive based Robotic system for Upper-Limb Stroke Rehabilitation. In Proceedings of the Annual International Conference of the IEEE Engineering in Medicine and Biology - Proceedings, 2022, Vol. 2022-July. https://doi.org/10.1109/EMBC48229.2022.9871426.
85. Schrader, M.; Sterr, A.; Kettlitz, R.; Wohlmeiner, A.; Buschfort, R.; Dohle, C.; Bamborschke, S. The effect of mirror therapy can be improved by simultaneous robotic assistance. *Restorative Neurology and Neuroscience* **2022**, *40*. https://doi.org/10.3233/RNN-221263.
86. Guo, N.; Wang, X.; Duanmu, D.; Huang, X.; Li, X.; Fan, Y.; Li, H.; Liu, Y.; Yeung, E.; To, M.; et al. SSVEP-Based Brain Computer Interface Controlled Soft Robotic Glove for Post-Stroke Hand Function Rehabilitation. *IEEE Transactions on Neural Systems and Rehabilitation Engineering* **2022**, *30*. https://doi.org/10.1109/TNSRE.2022.3185262.
87. Moggio, L.; de Sire, A.; Marotta, N.; Demeco, A.; Ammendolia, A. Exoskeleton versus end-effector robot-assisted therapy for finger-hand motor recovery in stroke survivors: systematic review and meta-analysis. *Topics in Stroke Rehabilitation* **2022**, *29*. https://doi.org/10.1080/10749357.2021.1967657.
88. Alexander, J.; Dawson, J.; Langhorne, P. Dynamic hand orthoses for the recovery of hand and arm function in adults after stroke: A systematic review and meta-analysis of randomised controlled trials. *Topics in Stroke Rehabilitation* **2022**, *29*. https://doi.org/10.1080/10749357.2021.1878669.
89. Singh, N.; Saini, M.; Kumar, N.; Srivastava, M.V.P.; Mehndiratta, A. Evidence of neuroplasticity with robotic hand exoskeleton for post-stroke rehabilitation: a randomized controlled trial. *J Neuroeng Rehabil* **2021**. https://doi.org/10.1186/s12984-021-00867-7.
90. Gueye, T.; Dedkova, M.; Rogalewicz, V.; Grunerova-Lippertova, M.; Angerova, Y. Early post-stroke rehabilitation for upper limb motor function using virtual reality and exoskeleton: equally efficient in older patients. *Neurol Neurochir Pol* **2021**. https://doi.org/10.5603/PJNNS.a2020.0096.
91. Chen, Z.J.; He, C.; Guo, F.; Xiong, C.H.; Huang, X.L. Exoskeleton-Assisted Anthropomorphic Movement Training (EAMT) for Poststroke Upper Limb Rehabilitation: A Pilot Randomized Controlled Trial. *Arch Phys Med Rehabil* **2021**. https://doi.org/10.1016/j.apmr.2021.06.001.
92. Sun, N.; Li, G.; Cheng, L. Design and Validation of a Self-Aligning Index Finger Exoskeleton for Post-Stroke Rehabilitation. *IEEE Trans Neural Syst Rehabil Eng* **2021**. https://doi.org/10.1109/TNSRE.2021.3097888.
93. Nasrallah, F.; Mohamed, A.; Yap, H.; Lai, H.S.; Yeow, R.H.; Lim, J. Effect of proprioceptive stimulation using a soft robotic glove on motor activation and brain connectivity in stroke survivors. *Journal of Neural Engineering* **2021**, *18*. https://doi.org/10.1088/1741-2552/ac456c.
94. Nazari, V.; Pouladian, M.; Zheng, Y.P.; Alam, M. A compact and lightweight rehabilitative exoskeleton to restore grasping functions for people with hand paralysis. *Sensors* **2021**, *21*. https://doi.org/10.3390/s21206900.

95. Islam, M.; Assad-Uz-Zaman, M.; Brahim, B.; Bouteraa, Y.; Wang, I.; Rahman, M. Design and development of an upper limb rehabilitative robot with dual functionality. In Proceedings of the Micromachines, 2021, Vol. 12. https://doi.org/10.3390/mi12080870.
96. Li, J.; Cao, Q.; Dong, M. Research on the Kinematic Compatibility of Position Solution of a Four-DOFs Mechanical Structure Mechanism. In Proceedings of the Journal of Physics: Conference Series, 2021, Vol. 1952. https://doi.org/10.1088/1742-6596/1952/3/032062.
97. Cantillo-Negrete, J.; Cariño Escobar, R.; Carrillo-Mora, P.; Rodríguez-Barragán, M.; Hernandez-Arenas, C.; Quinzaños Fresnedo, J.; Hernandez-Sanchez, I.; Galicia-Alvarado, M.; Miguel-Puga, A.; Arias-Carrion, O. Brain-Computer Interface Coupled to a Robotic Hand Orthosis for Stroke Patients? Neurorehabilitation: A Crossover Feasibility Study. *Frontiers in Human Neuroscience* **2021**, *15*. https://doi.org/10.3389/fnhum.2021.656975.
98. Proietti, T.; O'Neill, C.; Hohimer, C.; Nuckols, K.; Clarke, M.; Zhou, Y.; Lin, D.; Walsh, C. Sensing and Control of a Multi-Joint Soft Wearable Robot for Upper-Limb Assistance and Rehabilitation. *IEEE Robotics and Automation Letters* **2021**, *6*. https://doi.org/10.1109/LRA.2021.3061061.
99. Casas, R.; Sandison, M.; Chen, T.; Lum, P. Clinical Test of a Wearable, High DOF, Spring Powered Hand Exoskeleton (HandSOME II). *IEEE Transactions on Neural Systems and Rehabilitation Engineering* **2021**, *29*. https://doi.org/10.1109/TNSRE.2021.3110201.
100. He, C.; Xiong, C.H.; Chen, Z.J.; Fan, W.; Huang, X.L.; Fu, C. Preliminary Assessment of a Postural Synergy-Based Exoskeleton for Post-Stroke Upper Limb Rehabilitation. *IEEE Transactions on Neural Systems and Rehabilitation Engineering* **2021**, *29*. https://doi.org/10.1109/TNSRE.2021.3107376.
101. Grampurohit, N.; Bell, A.; Duff, S.; Mulcahey, M.; Thielen, C.; Kaplan, G.; Marino, R. Highlighting gaps in spinal cord injury research in activity-based interventions for the upper extremity: A scoping review. *NeuroRehabilitation* **2021**, *49*. https://doi.org/10.3233/NRE-210042.
102. Marotta, N.; Demeco, A.; Moggio, L.; Ammendolia, A. The adjunct of transcranial direct current stimulation to Robot-assisted therapy in upper limb post-stroke treatment. *Journal of Medical Engineering and Technology* **2021**, *45*. https://doi.org/10.1080/03091902.2021.1922527.
103. Sandison, M.; Phan, K.; Casas, R.; Nguyen, L.; Lum, M.; Pergami-Peries, M.; Lum, P.S. HandMATE: Wearable Robotic Hand Exoskeleton and Integrated Android App for At Home Stroke Rehabilitation. *Annu Int Conf IEEE Eng Med Biol Soc* **2020**. https://doi.org/10.1109/EMBC44109.2020.9175332.
104. Lambelet, C.; Temiraliuly, D.; Siegenthaler, M.; Wirth, M.; Woolley, D.G.; Lambercy, O.; Gassert, R.; Wenderoth, N. Characterization and wearability evaluation of a fully portable wrist exoskeleton for unsupervised training after stroke. *J Neuroeng Rehabil* **2020**. https://doi.org/10.1186/s12984-020-00749-4.
105. Lee, S.H.; Park, G.; Cho, D.Y.; Kim, H.Y.; Lee, J.Y.; Kim, S.; Park, S.B.; Shin, J.H. Comparisons between end-effector and exoskeleton rehabilitation robots regarding upper extremity function among chronic stroke patients with moderate-to-severe upper limb impairment. *Sci Rep* **2020**. https://doi.org/10.1038/s41598-020-58630-2.
106. Burns, M.; Zavoda, Z.; Nataraj, R.; Pochiraju, K.; Vinjamuri, R. HERCULES: A Three Degree-of-Freedom Pneumatic Upper Limb Exoskeleton for Stroke Rehabilitation(). *Annu Int Conf IEEE Eng Med Biol Soc* **2020**. https://doi.org/10.1109/EMBC44109.2020.9176549.
107. Cheng, N.; Phua, K.; Lai, H.S.; Tam, P.; Tang, K.; Cheng, K.; Yeow, R.H.; Ang, K.; Guan, C.; Lim, J. Brain-Computer Interface-Based Soft Robotic Glove Rehabilitation for Stroke. *IEEE Transactions on Biomedical Engineering* **2020**, *67*. https://doi.org/10.1109/TBME.2020.2984003.
108. Park, S.; Fraser, M.; Weber, L.; Meeker, C.; Bishop, L.; Geller, D.; Stein, J.; Ciocarlie, M. User-Driven Functional Movement Training with a Wearable Hand Robot after Stroke. *IEEE Transactions on Neural Systems and Rehabilitation Engineering* **2020**, *28*. https://doi.org/10.1109/TNSRE.2020.3021691.
109. Pierella, C.; Pirondini, E.; Kinany, N.; Coscia, M.; Giang, C.; Miehlbradt, J.; Magnin, C.; Nicolo, P.; Dalise, S.; Sgherri, G.; et al. A multimodal approach to capture post-stroke temporal dynamics of recovery. *Journal of Neural Engineering* **2020**, *17*. https://doi.org/10.1088/1741-2552/ab9ada.
110. O'Neill, C.; Proietti, T.; Nuckols, K.; Clarke, M.; Hohimer, C.; Cloutier, A.; Lin, D.; Walsh, C. Inflatable Soft Wearable Robot for Reducing Therapist Fatigue during Upper Extremity Rehabilitation in Severe Stroke. *IEEE Robotics and Automation Letters* **2020**, *5*. https://doi.org/10.1109/LRA.2020.2982861.
111. Yurkewich, A.; Kozak, I.; Hébert, D.; Wang, R.; Mihailidis, A. Hand Extension Robot Orthosis (HERO) Grip Glove: Enabling independence amongst persons with severe hand impairments after stroke. *Journal of NeuroEngineering and Rehabilitation* **2020**, *17*. https://doi.org/10.1186/s12984-020-00659-5.
112. Bhagat, N.; Yozbatiran, N.; Sullivan, J.; Paranjape, R.; Losey, C.; Hernandez, Z.; Keser, Z.; Grossman, R.; Francisco, G.; O-Malley, M.; et al. Neural activity modulations and motor recovery following brain-exoskeleton interface mediated stroke rehabilitation. *NeuroImage: Clinical* **2020**, *28*. https://doi.org/10.1016/j.nicl.2020.102502.

113. Ansari, M.; Mohd Sobri, N.; Mahyuddin, M. Dynamic compensation controller with feedback linearization technique of 3 degree of freedom exoskeleton robotic arm for upper limb rehabilitation purpose. *Lecture Notes in Mechanical Engineering* **2020**. https://doi.org/10.1007/978-981-13-9539-0_53.
114. Stewart, A.; Pretty, C.; Chen, X. A portable assist-as-need upper-extremity hybrid exoskeleton for FES-induced muscle fatigue reduction in stroke rehabilitation. *BMC Biomed Eng* **2019**. https://doi.org/10.1186/s42490-019-0028-6.
115. Singh, N.; Saini, M.; Anand, S.; Kumar, N.; Srivastava, M.; Mehndiratta, A. Robotic Exoskeleton for Wrist and Fingers Joint in Post-Stroke Neuro-Rehabilitation for Low-Resource Settings. *IEEE Transactions on Neural Systems and Rehabilitation Engineering* **2019**, *27*. https://doi.org/10.1109/TNSRE.2019.2943005.
116. Chen, T.; Casas, R.; Lum, P. An Elbow Exoskeleton for Upper Limb Rehabilitation with Series Elastic Actuator and Cable-Driven Differential. *IEEE Transactions on Robotics* **2019**, *35*. https://doi.org/10.1109/TRO.2019.2930915.
117. Ambrosini, E.; Russold, M.; Gfoehler, M.; Puchinger, M.; Weber, M.; Becker, S.; Krakow, K.; Immick, N.; Augsten, A.; Rossini, M.; et al. A Hybrid robotic system for arm training of stroke survivors: Concept and first evaluation. *IEEE Transactions on Biomedical Engineering* **2019**, *66*. https://doi.org/10.1109/TBME.2019.2900525.
118. Mochizuki, G.; Centen, A.; Resnick, M.; Lowrey, C.; Dukelow, S.; Scott, S. Movement kinematics and proprioception in post-stroke spasticity: Assessment using the Kinarm robotic exoskeleton. *Journal of NeuroEngineering and Rehabilitation* **2019**, *16*. https://doi.org/10.1186/s12984-019-0618-5.
119. Goltapeh, A.; Behzadipour, S.; Hajihosseinali, M. Design and Construction of a Planar Robotic Exoskeleton for Assessment of Upper Limb Movements. 2019. https://doi.org/10.1109/ICRoM48714.2019.9071807.
120. Zhang, L.; Li, J.; Ji, S.; Su, P.; Tao, C.; Ji, R. Design and human-machine compatibility analysis of Co-Exos II for upper-limb rehabilitation. *Assembly Automation* **2019**, *39*. https://doi.org/10.1108/AA-09-2018-0127.
121. Chen, J.; Hu, D.; Sun, W.; Tu, X.; He, J. A novel telerehabilitation system based on bilateral upper limb exoskeleton robot. 2019. https://doi.org/10.1109/RCAR47638.2019.9044072.
122. Miller, K.; Gallina, A.; Neva, J.; Ivanova, T.; Snow, N.; Ledwell, N.; Xiao, Z.; Menon, C.; Boyd, L.; Garland, S. Effect of repetitive transcranial magnetic stimulation combined with robot-assisted training on wrist muscle activation post-stroke. *Clinical Neurophysiology* **2019**, *130*. https://doi.org/10.1016/j.clinph.2019.04.712.
123. Santra, P.; Mukhopadhyay, A.; Debnath, B.; Herbert, M.; Bhaumik, S. Active Upper Arm Exoskeleton - Design and Kinematic Analysis. 2019. https://doi.org/10.1109/TENSYMP46218.2019.8971152.
124. Phan, T.; Nguyen, H.; Vermillion, B.; Lee, S. Passive elbow movement assistant (PEMA): A portable exoskeleton to compensate angle-dependent tone profile of the elbow joint post-stroke. In Proceedings of the IEEE International Conference on Rehabilitation Robotics, 2019, Vol. 2019-June. https://doi.org/10.1109/ICORR.2019.8779365.
125. Oliveira, A.; Rose, C.; Warburton, K.; Ogden, E.; Whitford, B.; Lee, R.; Deshpande, A. Exploring the capabilities of harmony for upper-limb stroke therapy. In Proceedings of the IEEE International Conference on Rehabilitation Robotics, 2019, Vol. 2019-June. https://doi.org/10.1109/ICORR.2019.8779558.
126. Irshaidat, M.; Soufian, M.; Al-Ibadi, A.; Nefti-Meziani, S. A novel elbow pneumatic muscle actuator for exoskeleton arm in post-stroke rehabilitation. 2019. https://doi.org/10.1109/ROBOSOFT.2019.8722813.
127. Carvalho, R.; Dias, N.; Cerqueira, J. Brain-machine interface of upper limb recovery in stroke patients rehabilitation: A systematic review. *Physiotherapy Research International* **2019**, *24*. https://doi.org/10.1002/pri.1764.
128. Brahim, B.; Saad, M.; Rahman, M.; Ochoa-Luna, C. Cartesian Trajectory Tracking of a 7-DOF Exoskeleton Robot Based on Human Inverse Kinematics. *IEEE Transactions on Systems, Man, and Cybernetics: Systems* **2019**, *49*. https://doi.org/10.1109/TSMC.2017.2695003.
129. Zhang, L.; Li, J.; Su, P.; Song, Y.; Dong, M.; Cao, Q. Improvement of human-machine compatibility of upper-limb rehabilitation exoskeleton using passive joints. *Robotics and Autonomous Systems* **2019**, *112*. https://doi.org/10.1016/j.robot.2018.10.012.
130. Meng, Q.; Xie, Q.; Shao, H.; Cao, W.; Wang, F.; Wang, L.; Yu, H.; Li, S. Pilot Study of a Powered Exoskeleton for Upper Limb Rehabilitation Based on the Wheelchair. *BioMed Research International* **2019**, *2019*. https://doi.org/10.1155/2019/9627438.
131. Brahim, B.; Saad, M.; Ochoa-Luna, C.; Rahman, M.; Brahmi, A. Novel adaptive iterative observer based on integral backstepping control of a wearable robotic exoskeleton. *International Journal of Computer Applications in Technology* **2019**, *60*. https://doi.org/10.1504/IJCAT.2019.100132.
132. Taha, Z.; P. P. Abdul Majeed, A.; Abdullah, M.; Azmi, K.; Zakaria, M.; Abdul Ghani, A.; Hassan, M.; Mohd Razman, M. The control of an upper extremity exoskeleton for stroke rehabilitation by means of a hybrid active force control. *Advances in Intelligent Systems and Computing* **2019**, *751*. https://doi.org/10.1007/978-3-319-78452-6_30.
133. Bernocchi, P.; Mulè, C.; Vanoglio, F.; Taveggia, G.; Luisa, A.; Scalvini, S. Home-based hand rehabilitation with a robotic glove in hemiplegic patients after stroke: a pilot feasibility study. *Top Stroke Rehabil* **2018**. https://doi.org/10.1080/10749357.2017.1389021.
134. Wang, D.; Meng, Q.; Meng, Q.; Li, X.; Yu, H. Design and Development of a Portable Exoskeleton for Hand Rehabilitation. *IEEE Transactions on Neural Systems and Rehabilitation Engineering* **2018**, *26*. https://doi.org/10.1109/TNSRE.2018.2878778.

135. Semrau, J.; Herter, T.; Scott, S.; Dukelow, S. Vision of the upper limb fails to compensate for kinesthetic impairments in subacute stroke. *Cortex* **2018**, *109*. https://doi.org/10.1016/j.cortex.2018.09.022.
136. Zheng, J.; Shi, P.; Yu, H. A Virtual Reality Rehabilitation Training System Based on Upper Limb Exoskeleton Robot. 2018, Vol. 1. https://doi.org/10.1109/IHMSC.2018.00058.
137. Puchinger, M.; Kurup, N.; Keck, T.; Zajc, J.; Russold, M.; Gfoehler, M. The Retrainer Light-Weight Arm Exoskeleton: Effect of Adjustable Gravity Compensation on Muscle Activations and Forces. In Proceedings of the Proceedings of the IEEE RAS and EMBS International Conference on Biomedical Robotics and Biomechatronics, 2018, Vol. 2018-August. https://doi.org/10.1109/BIOROB.2018.8487218.
138. Shuxiang, S.; Wang, Z.; Guo, J.; Fu, Q.; Li, N. Design of the speech control system for a upper limb rehabilitation robot based on wavelet de-noising. 2018. https://doi.org/10.1109/ICMA.2018.8484626.
139. Manna, S.; Dubey, V. Comparative study of actuation systems for portable upper limb exoskeletons. *Medical Engineering and Physics* **2018**, *60*. https://doi.org/10.1016/j.medengphy.2018.07.017.
140. Kuczynski, A.; Kirton, A.; Semrau, J.; Dukelow, S. Bilateral reaching deficits after unilateral perinatal ischemic stroke: A population-based case-control study. *Journal of NeuroEngineering and Rehabilitation* **2018**, *15*. https://doi.org/10.1186/s12984-018-0420-9.
141. Lessard, S.; Pansodtee, P.; Robbins, A.; Trombadore, J.; Kurniawan, S.; Teodorescu, M. A Soft Exosuit for Flexible Upper-Extremity Rehabilitation. *IEEE Transactions on Neural Systems and Rehabilitation Engineering* **2018**, *26*. https://doi.org/10.1109/TNSRE.2018.2854219.
142. Bogue.; R. Rehabilitation robots. *Industrial Robot* **2018**, *45*. https://doi.org/10.1108/IR-03-2018-0046.
143. Anam, K.; Rosyadi, A.; Sujanarko, B. The Design of a Low-Cost Therapy Robot for Hand Rehabilitation of a Post-Stroke Patient. 2018. https://doi.org/10.1109/CENIM.2018.8710833.
144. Ying, G.; Elamvazhuthi, I.; Khan, M.; Parasuraman, S. Development of EMG based Upper Arm Exoskeleton. 2018. https://doi.org/10.1109/RTECC.2018.8625671.
145. Ono, Y.; Wada, K.; Seki, N.; Imanishi Ito, M.; Tani Minakuchi, M.; Kono, M.; Tominaga, T. Hand Motor Rehabilitation of Patients with Stroke Using Physiologically Congruent Neurofeedback. 2018. https://doi.org/10.1109/SMC.2018.00016.
146. Ogden, E.; Chiu, D.; Clearman, R.; Card, C.; Deshpande, A. Evaluation of the harmony exoskeleton as an upper extremity rehabilitation tool after stroke. 2018. https://doi.org/10.1109/WEROB.2017.8383834.
147. Wu, Q.; Wang, X.; Chen, B.; Wu, H. Development of a Minimal-Intervention-Based Admittance Control Strategy for Upper Extremity Rehabilitation Exoskeleton. *IEEE Transactions on Systems, Man, and Cybernetics: Systems* **2018**, *48*. https://doi.org/10.1109/TSMC.2017.2771227.
148. Wolbrecht, E.; Rowe, J.; Chan, V.; Ingemanson, M.; Cramer, S.; Reinkensmeyer, D. Finger strength, individuation, and their interaction: Relationship to hand function and corticospinal tract injury after stroke. *Clinical Neurophysiology* **2018**, *129*. https://doi.org/10.1016/j.clinph.2018.01.057.
149. Silveira, A.; Abreu de Souza, M.; Fernandes, B.; Nohama, P. From the past to the future of therapeutic orthoses for upper limbs rehabilitation. *Research on Biomedical Engineering* **2018**, *34*. https://doi.org/10.1590/2446-4740.170084.
150. Boheng, W.; Chen, C.; Bo, Z.; Liang, L.; Xiang, G. Design and implementation of shoulder exoskeleton robot. *Lecture Notes in Computer Science* **2018**, *11357 LNAI*. https://doi.org/10.1007/978-3-030-05204-1_24.
Washabaugh, E.; Treadway, E.; Brent Gillespiec, R.; David Remy, C.; Krishnan, C. Self-powered robots to reduce motor slacking during upper-extremity rehabilitation: A proof of concept study. *Restorative Neurology and Neuroscience* **2018**, *36*. https://doi.org/10.3233/RNN-180830.
152. Lambelet, C.; Lyu, M.; Woolley, D.; Gassert, R.; Wenderoth, N. The eWrist - A wearable wrist exoskeleton with sEMG-based force control for stroke rehabilitation. *IEEE Int Conf Rehabil Robot* **2017**. https://doi.org/10.1109/ICORR.2017.8009334.
153. Prange-Lasonder, G.B.; Radder, B.; Kottink, A.I.R.; Melendez-Calderon, A.; Buurke, J.H.; Rietman, J.S. Applying a soft-robotic glove as assistive device and training tool with games to support hand function after stroke: Preliminary results on feasibility and potential clinical impact. *IEEE Int Conf Rehabil Robot* **2017**. https://doi.org/10.1109/ICORR.2017.8009444.
154. Frolov, A.A.; Mokienko, O.; Lyukmanov, R.; Biryukova, E.; Kotov, S.; Turbina, L.; Nadareyshvily, G.; Bushkova, Y. Post-stroke Rehabilitation Training with a Motor-Imagery-Based Brain-Computer Interface (BCI)-Controlled Hand Exoskeleton: A Randomized Controlled Multicenter Trial. *Front Neurosci* **2017**. https://doi.org/10.3389/fnins.2017.00400.
155. Housley, S.N.; Wu, D.; Richards, K.; Belagaje, S.; Ghovanloo, M.; Butler, A.J. Improving Upper Extremity Function and Quality of Life with a Tongue Driven Exoskeleton: A Pilot Study Quantifying Stroke Rehabilitation. *Stroke Res Treat* **2017**. https://doi.org/10.1155/2017/3603860.
156. Kenzie, J.; Semrau, J.; Hill, M.; Scott, S.; Dukelow, S. A composite robotic-based measure of upper limb proprioception. *Journal of NeuroEngineering and Rehabilitation* **2017**, *14*. https://doi.org/10.1186/s12984-017-0329-8.

157. Nam, H.; Koh, S.; Kim, Y.; Beom, J.; Lee, W.; Lee, S.U.; Kim, S. Biomechanical Reactions of Exoskeleton Neurorehabilitation Robots in Spastic Elbows and Wrists. *IEEE Transactions on Neural Systems and Rehabilitation Engineering* **2017**, *25*. https://doi.org/10.1109/TNSRE.2017.2714203.
158. Nam, H.; Koh, S.; Beom, J.; Kim, Y.; Park, J.; Koh, E.S.; Chung, S.; Kim, S. Recovery of proprioception in the upper extremity by robotic mirror therapy: A clinical pilot study for proof of concept. *Journal of Korean Medical Science* **2017**, *32*. https://doi.org/10.3346/jkms.2017.32.10.1568.
159. Bertani, R.; Melegari, C.; De Cola, M.; Bramanti, A.; Bramanti, P.; Calabrò, R. Effects of robot-assisted upper limb rehabilitation in stroke patients: a systematic review with meta-analysis. *Neurological Sciences* **2017**, *38*. https://doi.org/10.1007/s10072-017-2995-5.
160. Lee, D.; Bae, S.; Jang, S.; Chang, P. Design of a clinically relevant upper-limb exoskeleton robot for stroke patients with spasticity. In Proceedings of the IEEE International Conference on Rehabilitation Robotics, 2017. https://doi.org/10.1109/ICORR.2017.8009317.
161. Ambrosini, E.; Ferrante, S.; Zajc, J.; Bulgheroni, M.; Baccinelli, W.; D'Amico, E.; Schauer, T.; Wiesener, C.; Russold, M.; Gfoehler, M.; et al. The combined action of a passive exoskeleton and an EMG-controlled neuroprosthesis for upper limb stroke rehabilitation: First results of the RETRAINER project. In Proceedings of the IEEE International Conference on Rehabilitation Robotics, 2017. https://doi.org/10.1109/ICORR.2017.8009221.
162. Sullivan, J.; Bhagat, N.; Yozbatiran, N.; Paranjape, R.; Losey, C.; Grossman, R.; Contreras-Vidal, J.; Francisco, G.; O-Malley, M. Improving robotic stroke rehabilitation by incorporating neural intent detection: Preliminary results from a clinical trial. In Proceedings of the IEEE International Conference on Rehabilitation Robotics, 2017. https://doi.org/10.1109/ICORR.2017.8009233.
163. Bian, H.; Chen, Z.; Wang, H.; Zhao, T. Mechanical design of EFW Exo II: A hybrid exoskeleton for elbow-forearm-wrist rehabilitation. In Proceedings of the IEEE International Conference on Rehabilitation Robotics, 2017. https://doi.org/10.1109/ICORR.2017.8009328.
164. Zeiaee, A.; Soltani-Zarrin, R.; Langari, R.; Tafreshi, R. Design and kinematic analysis of a novel upper limb exoskeleton for rehabilitation of stroke patients. In Proceedings of the IEEE International Conference on Rehabilitation Robotics, 2017. https://doi.org/10.1109/ICORR.2017.8009339.
165. Just, F.; Özen, O.; Tortora, S.; Riener, R.; Rauter, G. Feedforward model based arm weight compensation with the rehabilitation robot ARMin. In Proceedings of the IEEE International Conference on Rehabilitation Robotics, 2017. https://doi.org/10.1109/ICORR.2017.8009224.
166. Klamroth-Marganska, V.; Baur, K.; Nagle, A.; Riener, R.; Toigo, M. Strength training of the upper extremity in virtual reality with an exoskeleton robot. In Proceedings of the International Conference on Virtual Rehabilitation, ICVR, 2017, Vol. 2017-June. https://doi.org/10.1109/ICVR.2017.8007500.
167. Song, W.K.; Song, J.Y. Improvement of upper extremity rehabilitation Robotic Exoskeleton, NREX. 2017. https://doi.org/10.1109/URAI.2017.7992674.

Frullo, J.; Elinger, J.; Pehlivan, A.; Fitle, K.; Nedley, K.; Francisco, G.; Sergi, F.; O-Malley, M. Effects of assist-as-needed upper extremity robotic therapy after incomplete spinal cord injury: A parallel-group controlled trial. *Frontiers in Neurorobotics* **2017**, *11*. https://doi.org/10.3389/fnbot.2017.00026.

169. Proietti, T.; Guigon, E.; Roby-Brami, A.; Jarrassé, N. Modifying upper-limb inter-joint coordination in healthy subjects by training with a robotic exoskeleton. *Journal of NeuroEngineering and Rehabilitation* **2017**, *14*. https://doi.org/10.1186/s12984-017-0254-x.
170. Kuczynski, A.; Carlson, H.; Lebel, C.; Hodge, J.; Dukelow, S.; Semrau, J.; Kirton, A. Sensory tractography and robot-quantified proprioception in hemiparetic children with perinatal stroke. *Human Brain Mapping* **2017**, *38*. https://doi.org/10.1002/hbm.23530.
171. Tageldeen, M.; Nallagownden, N.; Elamvazhuthi, I.; Ganesan, T. Design and control of an upper arm exoskeleton using Fuzzy logic techniques. 2017. https://doi.org/10.1109/ROMA.2016.7847838.
172. Brahim, B.; Saad, M.; Ochoa-Luna, C.; Abdelkrim, B.; Rahman, M. Adaptive iterative observer based on integral backstepping control for upper extremity exoskelton robot. 2017. https://doi.org/10.1109/ICMIC.2016.7804240.
173. Li, Q.; Xiong, C.H.; Liu, K. Eye gaze tracking based interaction method of an upper-limb exoskeletal rehabilitation robot. *Lecture Notes in Computer Science* **2017**, *10462 LNAI*. https://doi.org/10.1007/978-3-319-65289-4_33.
174. Yuan, N.; Thielbar, K.; Zhang, L.Q.; Kamper, D. Use of an actuated glove to facilitate hand rehabilitation after stroke. In Proceedings of the Biosystems and Biorobotics, 2017, Vol. 15. https://doi.org/10.1007/978-3-319-46669-9_91.
175. Kitisomprayoonkul, W.; Bhodhiassana, P.; Sangveraphunsiri, V. Upper extremity training with CUREs robot in subacute stroke: A pilot study. In Proceedings of the Biosystems and Biorobotics, 2017, Vol. 15. https://doi.org/10.1007/978-3-319-46669-9_54.
176. Saharan, L.; Sharma, A.; de Andrade, M.; Baughman, R.; Tadesse, Y. Design of a 3D printed lightweight orthotic device based on twisted and coiled polymer muscle: IGrab hand orthosis. In Proceedings of the Proceedings of SPIE - The International Society for Optical Engineering, 2017, Vol. 10164. https://doi.org/10.1117/12.2260266.
177. Tianyao, C.; Lum, P.S. Hand rehabilitation after stroke using a wearable, high DOF, spring powered exoskeleton. *Annu Int Conf IEEE Eng Med Biol Soc* **2016**. https://doi.org/10.1109/EMBC.2016.7590768.

178. Xiong, P.; Gao, S.; Liu, Z.; Hu, L.; Ding, X. A novel scheme of finger recovery based on symmetric rehabilitation: Specially for hemiplegia. In Proceedings of the Proceedings of the International Conference on Sensing Technology, ICST, 2016. https://doi.org/10.1109/ICSensT.2016.7796309.
179. Hu, Y.; Huang, Z.; Li, Z.; Yang, C. Biomechtronic design of a wearable upper dual-arm exoskeleton robot for lifting tasks. 2016. https://doi.org/10.1109/ICARM.2016.7606932.
180. Huang, J.; Tu, X.; He, J. Design and Evaluation of the RUPERT Wearable Upper Extremity Exoskeleton Robot for Clinical and In-Home Therapies. *IEEE Transactions on Systems, Man, and Cybernetics: Systems* **2016**, *46*. https://doi.org/10.1109/TSMC.2015.2497205.
181. Sheng, B.; Zhang, Y.; Meng, W.; Deng, C.; Xie, S. Bilateral robots for upper-limb stroke rehabilitation: State of the art and future prospects. *Medical Engineering and Physics* **2016**, *38*. https://doi.org/10.1016/j.medengphy.2016.04.004.
182. Longhi, M.; Merlo, A.; Prati, P.; Giacobbi, M.; Mazzoli, D. Instrumental indices for upper limb function assessment in stroke patients: A validation study. *Journal of NeuroEngineering and Rehabilitation* **2016**, *13*. https://doi.org/10.1186/s12984-016-0163-4.
183. Bouteraa, Y.; Ben Abdallah, I. Exoskeleton robots for upper-limb rehabilitation. 2016. https://doi.org/10.1109/SSD.2016.7473769.
184. Lew, B.; Alavi, N.; Randhawa, B.; Menon, C. An exploratory investigation on the use of closed-loop electrical stimulation to assist individuals with stroke to perform fine movements with their hemiparetic arm. *Frontiers in Bioengineering and Biotechnology* **2016**, *4*. https://doi.org/10.3389/fbioe.2016.00020.
185. Pirondini, E.; Coscia, M.; Marcheschi, S.; Roas, G.; Salsedo, F.; Frisoli, A.; Bergamasco, M.; Micera, S. Evaluation of the effects of the Arm Light Exoskeleton on movement execution and muscle activities: A pilot study on healthy subjects. *Journal of NeuroEngineering and Rehabilitation* **2016**, *13*. https://doi.org/10.1186/s12984-016-0117-x.
186. Fukuda, H.; Morishita, T.; Ogata, T.; Saita, K.; Hyakutake, K.; Watanabe, J.; Shiota, E.; Inoue, T. Tailor-made rehabilitation approach using multiple types of hybrid assistive limb robots for acute stroke patients: A pilot study. *Assistive Technology* **2016**, *28*. https://doi.org/10.1080/10400435.2015.1080768.
187. Hunt, J.; Artemiadis, P.; Lee, H. Development of a novel shoulder exoskeleton using parallel actuation and slip, 2016. https://doi.org/10.1115/DSCC2016-9894.
188. Proietti, T.; Crocher, V.; Roby-Brami, A.; Jarrassé, N. Upper-limb robotic exoskeletons for neurorehabilitation: A review on control strategies. *IEEE Reviews in Biomedical Engineering* **2016**, *9*. https://doi.org/10.1109/RBME.2016.2552201.
189. Pignolo, L.; Lucca, L.; Basta, G.; Sena, S.; Pugliese, M.; Sannita, W.; Dolce, G. A new treatment in the rehabilitation of the paretic upper limb after stroke: The ARAMIS prototype and treatment protocol. *Annali dell'Istituto Superiore di Sanita* **2016**, *52*. https://doi.org/10.4415/ANN_16_02_25.
190. Nycz, C.; Delph, M.; Fischer, G. Modeling and design of a tendon actuated soft robotic exoskeleton for hemiparetic upper limb rehabilitation. In Proceedings of the Annual International Conference of the IEEE Engineering in Medicine and Biology - Proceedings, 2015, Vol. 2015-November. https://doi.org/10.1109/EMBC.2015.7319243.
191. Marini, F.; Squeri, V.; Cappello, L.; Morasso, P.; Riva, A.; Doglio, L.; Masia, L. Adaptive wrist robot training in pediatric rehabilitation. In Proceedings of the IEEE International Conference on Rehabilitation Robotics, 2015, Vol. 2015-September. https://doi.org/10.1109/ICORR.2015.7281195.
192. Prange-Lasonder, G.; Smulders, L.; van Wijngaarden, J.; Lijbers, G.; Nijenhuis, S.; Veltink, P.; Buurke, J.; Stienen, A. User requirements for assistance of the supporting hand in bimanual daily activities via a robotic glove for severely affected stroke patients. In Proceedings of the IEEE International Conference on Rehabilitation Robotics, 2015, Vol. 2015-September. https://doi.org/10.1109/ICORR.2015.7281225.
193. Paredes, L.; Farina, D.; Shin, Y.I.; Turolla, A. Efficacy of torque versus myocontrol for active, robotic-assisted rehabilitation of the shoulder after stroke: An experimental study. In Proceedings of the International IEEE/EMBS Conference on Neural Engineering, NER, 2015, Vol. 2015-July. https://doi.org/10.1109/NER.2015.7146701.
194. Jarrassé, N.; Proietti, T.; Crocher, V.; Robertson, J.; Sahbani, A.; Morel, G.; Roby-Brami, A. Robotic exoskeletons: a perspective for the rehabilitation of arm coordination in stroke patients. *Front Hum Neurosci* **2014**. https://doi.org/10.3389/fnhum.2014.00947.
195. Jones, C.L.; Wang, F.; Morrison, R.; Sarkar, N.; Kamper, D.G. Design and Development of the Cable Actuated Finger Exoskeleton for Hand Rehabilitation Following Stroke. *IEEE ASME Trans Mechatron* **2014**. https://doi.org/10.1109/TMECH.2012.2224359.
196. Ushiba, J.; Morishita, A.; Maeda, T. A task-oriented brain-computer interface rehabilitation system for patients with stroke hemiplegia. 2014. https://doi.org/10.1109/VITAE.2014.6934416.
197. Xiao, Z.; Elnady, A.; Webb, J.; Menon, C. Towards a brain computer interface driven exoskeleton for upper extremity rehabilitation. In Proceedings of the Proceedings of the IEEE RAS and EMBS International Conference on Biomedical Robotics and Biomechatronics, 2014. https://doi.org/10.1109/biorob.2014.6913815.
198. Keller, U.; Riener, R. Design of the pediatric arm rehabilitation robot ChARMin. In Proceedings of the Proceedings of the IEEE RAS and EMBS International Conference on Biomedical Robotics and Biomechatronics, 2014. https://doi.org/10.1109/biorob.2014.6913832.

199. Klamroth-Marganska, V.; Blanco, J.; Campen, K.; Curt, A.; Dietz, V.; Ettlin, T.; Felder, M.; Fellinghauer, B.; Guidali, M.; Kollmar, A.; et al. Three-dimensional, task-specific robot therapy of the arm after stroke: A multicentre, parallel-group randomised trial. *The Lancet Neurology* **2014**, *13*. https://doi.org/10.1016/S1474-4422(13)70305-3.
200. Park, J.H.; Lee, K.S.; Jeon, K.H.; Kim, D.; Park, H.S. Low cost and light-weight multi-DOF exoskeleton for comprehensive upper limb rehabilitation. 2014. https://doi.org/10.1109/URAI.2014.7057415.
201. French, J.; Rose, C.; O-Malley, M. System characterization of MAHI Exo-II: A robotic exoskeleton for upper extremity rehabilitation, 2014. https://doi.org/10.1115/DSCC2014-6267.
202. Chong, Y.; Chay, K.; Lim, Y. Design and development of wearable upper extremities powered exoskeleton - A cost-effective rehabilitation system. 2014. https://doi.org/10.1109/IECBES.2014.7047598.
203. Godfrey, S.B.; Holley, R.J.; Lum, P.S. Clinical effects of using HEXORR (Hand Exoskeleton Rehabilitation Robot) for movement therapy in stroke rehabilitation. *Am J Phys Med Rehabil* **2013**. https://doi.org/10.1097/PHM.0b013e31829e7a07.
204. Weiss, P.; Heyer, L.; Munte, T.F.; Heldmann, M.; Schweikard, A.; Maehle, E. Towards a parameterizable exoskeleton for training of hand function after stroke. *IEEE Int Conf Rehabil Robot* **2013**. https://doi.org/10.1109/ICORR.2013.6650505.
205. Beekhuis, J.H.; Westerveld, A.J.; van der Kooij, H.; Stienen, A.H. Design of a self-aligning 3-DOF actuated exoskeleton for diagnosis and training of wrist and forearm after stroke. *IEEE Int Conf Rehabil Robot* **2013**. https://doi.org/10.1109/ICORR.2013.6650357.
206. Milot, M.H.; Spencer, S.J.; Chan, V.; Allington, J.P.; Klein, J.; Chou, C.; Bobrow, J.E.; Cramer, S.C.; Reinkensmeyer, D.J. A crossover pilot study evaluating the functional outcomes of two different types of robotic movement training in chronic stroke survivors using the arm exoskeleton BONES. *J Neuroeng Rehabil* **2013**. https://doi.org/10.1186/1743-0003-10-112.
207. Martinez, J.A.; Ng, P.; Lu, S.; Campagna, M.S.; Celik, O. Design of Wrist Gimbal: a forearm and wrist exoskeleton for stroke rehabilitation. *IEEE Int Conf Rehabil Robot* **2013**. https://doi.org/10.1109/ICORR.2013.6650459.
208. Ockenfeld, C.; Tong, R.; Susanto, E.; Ho, S.K.; Hu, X. Fine finger motor skill training with exoskeleton robotic hand in chronic stroke: Stroke rehabilitation. In Proceedings of the IEEE International Conference on Rehabilitation Robotics, 2013. https://doi.org/10.1109/ICORR.2013.6650392.
209. Song, W.K.; Song, W.J.; Jung, J.Y. NREX: NRC Robotic Exoskeleton. 2013. https://doi.org/10.1109/URAI.2013.6677309.
210. Frisoli, A.; Procopio, C.; Chisari, C.; Creatini, I.; Bonfiglio, L.; Bergamasco, M.; Rossi, B.; Chiara Carboncini, M. Positive effects of robotic exoskeleton training of upper limb reaching movements after stroke. *Journal of NeuroEngineering and Rehabilitation* **2012**, *9*. https://doi.org/10.1186/1743-0003-9-36.
211. Rahman, M.; Kittel-Ouimet, T.; Saad, M.; Kenné, J.P.; Archambault, P. Development and control of a robotic exoskeleton for shoulder, elbow and forearm movement assistance. *Applied Bionics and Biomechanics* **2012**, *9*. https://doi.org/10.1155/2012/956310.
212. Frisoli, A.; Sotgiu, E.; Procopio, C.; Bergamasco, M.; Rossi, B.; Chisari, C. Design and implementation of a training strategy in chronic stroke with an arm robotic exoskeleton. *IEEE Int Conf Rehabil Robot* **2011**. https://doi.org/10.1109/ICORR.2011.5975512.
213. Ho, N.S.; Tong, K.Y.; Hu, X.L.; Fung, K.L.; Wei, X.J.; Rong, W.; Susanto, E.A. An EMG-driven exoskeleton hand robotic training device on chronic stroke subjects: task training system for stroke rehabilitation. *IEEE Int Conf Rehabil Robot* **2011**. https://doi.org/10.1109/ICORR.2011.5975340.
214. Brokaw, E.B.; Black, I.; Holley, R.J.; Lum, P.S. Hand Spring Operated Movement Enhancer (HandSOME): a portable, passive hand exoskeleton for stroke rehabilitation. *IEEE Trans Neural Syst Rehabil Eng* **2011**. https://doi.org/10.1109/TNSRE.2011.2157705.
215. Crocher, V.; Jarrassé, N.; Sahbani, A.; Roby-Brami, A.; Morel, G. Changing human upper-limb synergies with an exoskeleton using viscous fields. In Proceedings of the Proceedings - IEEE International Conference on Robotics and Automation, 2011. https://doi.org/10.1109/ICRA.2011.5979626.
216. Pehlivan, A.; Celik, O.; O-Malley, M. Mechanical design of a distal arm exoskeleton for stroke and spinal cord injury rehabilitation. In Proceedings of the IEEE International Conference on Rehabilitation Robotics, 2011. https://doi.org/10.1109/ICORR.2011.5975428.
217. Moubarak, S.; Pham, M.; Moreau, R.; Redarce, T. Gravity compensation of an upper extremity exoskeleton. 2010. https://doi.org/10.1109/IEMBS.2010.5626036.
218. Balasubramanian, S.; Zhang, H.; Buchanan, S.; Austin, H.; Herman, R.; He, J. Cooperative and active assistance based interactive therapy. 2010. https://doi.org/10.1109/ICCME.2010.5558824.
219. Stienen, A.; Hekman, E.; Braak, H.; Aalsma, A.; Van Der Helm, F.; van der Kooij, H. Design of a rotational hydroelastic actuator for a powered exoskeleton for upper limb rehabilitation. *IEEE Transactions on Biomedical Engineering* **2010**, *57*. https://doi.org/10.1109/TBME.2009.2018628.
220. Vallery, H.; Veneman, J.; van Asseldonk, E.; Ekkelenkamp, R.; Buss, M.; van Der Kooij, H. Compliant actuation of rehabilitation robots. *IEEE Robotics Automation Magazine* **2008**. https://doi.org/10.1109/MRA.2008.927689.
221. Dinh, B.K.; Xiloyannis, M.; Cappello, L.; Antuvan, C.W.; Yen, S.C.; Masia, L. Adaptive backlash compensation in upper limb soft wearable exoskeletons. *Robotics and Autonomous Systems* **2017**. https://doi.org/https://doi.org/10.1016/j.robot.2017.03.012.

222. Xiloyannis, M.; Cappello, L.; Binh, K.D.; Antuvan, C.W.; Masia, L. Preliminary design and control of a soft exosuit for assisting elbow movements and hand grasping in activities of daily living. *Journal of Rehabilitation and Assistive Technologies Engineering* **2017**. https://doi.org/10.1177/2055668316680315.
223. Xu, W.; Cai, C.; Zou, Y. Cascade controller design and stability analysis in FES-aided upper arm stroke rehabilitation robotic system. *Nonlinear Dynamics* **2015**, *79*. https://doi.org/10.1007/s11071-014-1726-1.
Akgün, ; Demirbüken, I.; Timurtaş, E.; Pehlivan, M.K.; Pehlivan, A.; Polat, G.; Francisco, G.; Yozbatiran, N. Exoskeleton-assisted upper limb rehabilitation after stroke: a randomized controlled trial. *Neurological Research* **2024**, *46*, 1074–1082. https://doi.org/10.1080/01616412.2024.2381385.
225. Gasperina, D.; Gandolla, M.; Longatelli, V.; Panzenbeck, M.; Luciani, B.; Braghin, F.; Pedrocchi, A. AGREE: A Compliant-Controlled Upper-Limb Exoskeleton for Physical Rehabilitation of Neurological Patients. *IEEE Transactions on Medical Robotics and Bionics* **2023**, *5*, 143–154. https://doi.org/10.1109/tmrb.2023.3239888.
226. Qian, W.; Liao, J.; Lu, L.; Ai, L.; Li, M.; Xiao, X.; Guo, Z. CURER: A Lightweight Cable-Driven Compliant Upper Limb Rehabilitation Exoskeleton Robot. *IEEE/ASME Transactions on Mechatronics* **2023**, *28*, 1730–1741. https://doi.org/10.1109/tmech.2022.3224423.
227. Nam, C.; Zhang, B.; Chow, T.; Ye, F.; Huang, Y.; Guo, Z.; Li, W.; Rong, W.; Hu, X.; Poon, W.S. Home-based self-help telerehabilitation of the upper limb assisted by an electromyography-driven wrist/hand exoneuromusculoskeleton after stroke. *Journal of NeuroEngineering and Rehabilitation* **2021**, *18*. https://doi.org/10.1186/s12984-021-00930-3.
228. Ates, S.; Haarman, C.; Stienen, A. SCRIPT passive orthosis: design of interactive hand and wrist exoskeleton for rehabilitation at home after stroke. *Autonomous Robots* **2017**, *41*, 711–723. https://doi.org/10.1007/s10514-016-9589-6.
229. Bundy, D.; Souders, L.; Baranyai, K.; Leonard, L.; Schalk, G.; Coker, R.; Moran, D.; Huskey, T.; Leuthardt, E. Contralesional Brain–Computer Interface Control of a Powered Exoskeleton for Motor Recovery in Chronic Stroke Survivors. *Stroke* **2017**, *48*, 1908–1915. https://doi.org/10.1161/strokeaha.116.016304.
230. Wu, Q.; Zheng, L.; Zhu, Y.; Xu, Z.; Zhang, Q.; Wu, H. Development of a Reconfigurable 7-DOF Upper Limb Rehabilitation Exoskeleton With Gravity Compensation Based on DMP. *IEEE Transactions on Medical Robotics and Bionics* **2025**, *7*, 303–314. https://doi.org/10.1109/tmrb.2024.3517157.
231. Hamid, I.; Triwiyanto.; Utomo, B. Improving the Accuracy of Upper Limb Exoskeleton Movements Using Machine Learning Based on EMG for Stroke Patient Rehabilitation. *Jurnal Teknokes* **2025**. https://doi.org/10.35882/76cntw33.
232. Li, N.; Yang, Y.; Li, G.; Yang, T.; Wang, Y.; Chen, W.; Yu, P.; Xue, X.; Zhang, C.; Wang, W.; et al. Multi-Sensor Fusion-Based Mirror Adaptive Assist-as-Needed Control Strategy of a Soft Exoskeleton for Upper Limb Rehabilitation. *IEEE Transactions on Automation Science and Engineering* **2024**, *21*, 475–487. https://doi.org/10.1109/tase.2022.3225727.
233. Chen, Y.; Miao, S.; Ye, J.; Chen, G.; Cheng, J.; Du, K.; Li, X. Upper-Limb Rehabilitation with a Dual-Mode Individualized Exoskeleton Robot: A Generative-Model-Based Solution. *ArXiv* **2024**, *abs/2409.03193*. https://doi.org/10.48550/arxiv.2409.03193.
234. Pan, J.; Astarita, D.; Baldoni, A.; Dell'Agnello, F.; Crea, S.; Vitiello, N.; Trigili, E. NESM-: An Upper-Limb Exoskeleton With Compliant Actuators for Clinical Deployment. *IEEE Robotics and Automation Letters* **2022**, *7*, 7708–7715. https://doi.org/10.1109/lra.2022.3183926.
235. Rehmat, N.; Zuo, J.; Meng, W.; Liu, Q.; Xie, S.; Liang, H. Upper limb rehabilitation using robotic exoskeleton systems: a systematic review. *International Journal of Intelligent Robotics and Applications* **2018**, *2*, 283–295. https://doi.org/10.1007/s41315-018-0064-8.
236. De Caro, J.D.S.; Castillo, A.; Castañeda, M.; Quintero, M.; Paez, E.E.G.; Sampayo, I.; Hernandez, J.; Rahman, M. Cable driven exoskeleton for upper-limb rehabilitation: A design review. *Robotics Auton. Syst.* **2020**, *126*, 103445. https://doi.org/10.1016/j.robot.2020.103445.
237. Li, N.; Yang, T.; Yu, P.; Chang, J.; Zhao, L.; Zhao, X.; Elhajj, I.; Xi, N.; Liu, L. Bio-inspired upper limb soft exoskeleton to reduce stroke-induced complications. *Bioinspiration Biomimetics* **2018**, *13*. https://doi.org/10.1088/1748-3190/aad8d4.
238. Sarasola-Sanz, A.; Ray, A.; Insausti-Delgado, A.; Irastorza-Landa, N.; Mahmoud, W.; Brötz, D.; Bibián-Nogueras, C.; Helmhold, F.; Zrenner, C.; Ziemann, U.; et al. A hybrid brain-muscle-machine interface for stroke rehabilitation: Usability and functionality validation in a 2-week intensive intervention. *Frontiers in Bioengineering and Biotechnology* **2024**, *12*. https://doi.org/10.3389/fbioe.2024.1330330.
239. McConnell, A.; Moioli, R.; Brasil, F.; Vallejo, M.; Corne, D.; Vargas, P.; Stokes, A. Robotic devices and brain-machine interfaces for hand rehabilitation post-stroke. *Journal of rehabilitation medicine* **2017**, *49 6*, 449–460. https://doi.org/10.2340/16501977-2229.
240. Pei, S.; Wang, J.; Yang, Y.; Dong, A.; Guo, B.; Guo, J.; Yao, Y. A Human-Centered Kinematics Design Optimization of Upper Limb Rehabilitation Exoskeleton Based on Configuration Manifold. *IEEE Open Journal of the Computer Society* **2025**, *6*, 282–293. https://doi.org/10.1109/ojcs.2024.3465661.
241. Li, J.; Cao, Q.; Dong, M.; Zhang, C. Compatibility evaluation of a 4-DOF ergonomic exoskeleton for upper limb rehabilitation. *Mechanism and Machine Theory* **2021**, *156*, 104146. https://doi.org/10.1016/j.mechmachtheory.2020.104146.

242. Hsu, H.; Koh, C.L.; Yang, K.C.; Lin, Y.C.; Hsu, C.H.; Su, F.C.; Kuo, L. Effects of an assist-as-needed equipped Tenodesis-Induced-Grip Exoskeleton Robot (TIGER) on upper limb function in patients with chronic stroke. *Journal of NeuroEngineering and Rehabilitation* **2024**, *21*. https://doi.org/10.1186/s12984-023-01298-2.
243. Rusinowska, B.; Tybulczuk, B.; Białowąs, P. Clinical outcomes in post-stroke rehabilitation using upper-limb exoskeletons: review of latest reports. (Exoskeletons in post-stroke rehabilitation). *Medical Science Pulse* **2024**. https://doi.org/10.5604/01.3001.0054.9895.
244. Tang, Z.; Hong, X.; Lv, S.; Cui, Z.; Sun, H.; Shao, J. Hand rehabilitation exoskeleton system based on EEG spatiotemporal characteristics. *Expert Syst. Appl.* **2025**, *270*, 126574. https://doi.org/10.1016/j.eswa.2025.126574.
245. Wahyu, E.; Sumartini, S.; Widyo, P.; Herdiman, L. Exoskeleton Design Using Kinematics Analysis for Upper Limb Rehabilitation in Post-Stroke Patients. *E3S Web of Conferences* **2023**. https://doi.org/10.1051/e3sconf/202346502027.
246. Saldarriaga, A.; Gutierrez-Velasquez, E.; Colorado, H. Soft Hand Exoskeletons for Rehabilitation: Approaches to Design, Manufacturing Methods, and Future Prospects. *Robotics* **2024**, *13*, 50. https://doi.org/10.3390/robotics13030050.
247. Gupta, A.; Singh, A.; Verma, V.; Mondal, A.; Gupta, M. Developments and clinical evaluations of robotic exoskeleton technology for human upper-limb rehabilitation. *Advanced Robotics* **2020**, *34*, 1023–1040. https://doi.org/10.1080/01691864.2020.1749926.
248. Ambrosini, E.; Gasperina, S.; Gandolla, M.; Pedrocchi, A. Upper-Limb Exoskeletons for Stroke Rehabilitation. *IFMBE Proceedings* **2019**. https://doi.org/10.1007/978-3-030-31635-8_209.
249. Lo, H.; Xie, S. Exoskeleton robots for upper-limb rehabilitation: state of the art and future prospects. *Medical engineering physics* **2012**, *34 3*, 261–268. https://doi.org/10.1016/j.medengphy.2011.10.004.
250. Grimm, F.; Kraugmann, J.; Naros, G.; Gharabaghi, A. Clinical validation of kinematic assessments of post-stroke upper limb movements with a multi-joint arm exoskeleton. *Journal of NeuroEngineering and Rehabilitation* **2020**, *18*. https://doi.org/10.1186/s12984-021-00875-7.
251. Pei, S.; Wang, J.; Guo, J.; Yin, H.; Yao, Y. A Human-like Inverse Kinematics Algorithm of an Upper Limb Rehabilitation Exoskeleton. *Symmetry* **2023**, *15*, 1657. https://doi.org/10.3390/sym15091657.
252. Tran, P.; Jeong, S.; Herrin, K.; Desai, J. Review: Hand Exoskeleton Systems, Clinical Rehabilitation Practices, and Future Prospects. *IEEE Transactions on Medical Robotics and Bionics* **2021**, *3*, 606–622. https://doi.org/10.1109/tmrb.2021.3100625.
253. Barsotti, M.; Leonardis, D.; Loconsole, C.; Solazzi, M.; Sotgiu, E.; Procopio, C.; Chisari, C.; Bergamasco, M.; Frisoli, A. A full upper limb robotic exoskeleton for reaching and grasping rehabilitation triggered by MI-BCI. *2015 IEEE International Conference on Rehabilitation Robotics (ICORR)* **2015**, pp. 49–54. https://doi.org/10.1109/icorr.2015.7281174.
254. Othman, A.; Zakaria, N.A.C.; Hashim, N.; Mohamaddan, S. Exoskeleton Robotics Intervention as an Adjunctive Treatment in Enhancing Post-Stroke Upper Limb Neurorecovery. *2021 21st International Conference on Control, Automation and Systems (ICCAS)* **2021**, pp. 1391–1396. https://doi.org/10.23919/iccas52745.2021.9650066.
255. Mubin, O.; Alnajjar, F.; Jishtu, N.; Alsinglawi, B.; Mahmud, A.A. Exoskeletons With Virtual Reality, Augmented Reality, and Gamification for Stroke Patients' Rehabilitation: Systematic Review. *JMIR Rehabilitation and Assistive Technologies* **2019**, *6*. https://doi.org/10.2196/12010.
256. Islam, R.; Rahmani, M.; Rahman, M. A Novel Exoskeleton with Fractional Sliding Mode Control for Upper Limb Rehabilitation. *Robotica* **2020**, *38*, 2099–2120. https://doi.org/10.1017/s0263574719001851.

Xiao, F.; Gao, Y.; Wang, Y.; Zhu, Y.; Zhao, J. Design and evaluation of a 7-DOF cable-driven upper limb exoskeleton. *Journal of Mechanical Science and Technology* **2018**, *32*, 855–864. https://doi.org/10.1007/s12206-018-0136-y.

257. Vargas, M.; Mayorga, J.; Oscco, B.; Cuyotupac, V.; Nacarino, A.; Allcca, D.; Gamarra-Vásquez, L.; Tejada-Marroquin, G.; Reategui, M.; Maldonado-Gómez, R.; et al. Bio-Mechatronics Development of Robotic Exoskeleton System With Mobile-Prismatic Joint Mechanism for Passive Hand Wearable-Rehabilitation. *Emerging Science Journal* **2024**. https://doi.org/10.28991/esj-2024-08-06-02.
259. Stewart, A.; Pretty, C.; Adams, M.; Chen, X. Hybrid Exoskeletons for Upper Limb Stroke Rehabilitation. *Research Anthology on Rehabilitation Practices and Therapy* **2021**. https://doi.org/10.4018/978-1-5225-2993-4.ch004.
260. Nuñez-Quispe, J.; Figueroa, A.; Campusano, D.; Huamanchumo, J.; Soto, A.; Chate, E.; Acuña, J.; Lleren, J.; Albites-Sanabria, J.; Milián-Ccopa, L.P.; et al. Structural Design and Sliding Mode Control Approach of a 4-DoF Upper-Limb Exoskeleton for Post-stroke Rehabilitation. *Multibody Mechatronic Systems* **2021**. https://doi.org/10.1007/978-3-030-88751-3_22.
261. Pirondini, E.; Coscia, M.; Marcheschi, S.; Roas, G.; Salsedo, F.; Frisoli, A.; Bergamasco, M.; Micera, S. Evaluation of a New Exoskeleton for Upper Limb Post-stroke Neuro-rehabilitation: Preliminary Results **2014**. pp. 637–645. https://doi.org/10.1007/978-3-319-08072-7_91.
262. Haghshenas-Jaryani, M.; Patterson, R.; Bugnariu, N.; Wijesundara, M. A pilot study on the design and validation of a hybrid exoskeleton robotic device for hand rehabilitation. *Journal of hand therapy : official journal of the American Society of Hand Therapists* **2020**. https://doi.org/10.1016/j.jht.2020.03.024.

263. Chiri, A.; Vitiello, N.; Giovacchini, F.; Roccella, S.; Vecchi, F.; Carrozza, M. Mechatronic Design and Characterization of the Index Finger Module of a Hand Exoskeleton for Post-Stroke Rehabilitation. *IEEE/ASME Transactions on Mechatronics* **2012**, *17*, 884–894. https://doi.org/10.1109/tmech.2011.2144614.
264. Borzenko, E.; Zhdanov, D.; Makarov, R.; Seleznev, A.; Utukin, V.; Bureev, A. Mechatronic Hand Exoskeleton for Rehabilitation after Stroke. *Mekhatronika, Avtomatizatsiya, Upravlenie* **2024**. https://doi.org/10.17587/mau.25.101-107.
265. Xu, P.; Xia, D.; Li, J.; Zhou, J.; Xie, L. Execution and perception of upper limb exoskeleton for stroke patients: a systematic review. *Intelligent Service Robotics* **2022**, *15*, 557–578. https://doi.org/10.1007/s11370-022-00435-5.
266. Bouteraa, Y.; Abdallah, I.B.; Elmogy, A. Training of Hand Rehabilitation Using Low Cost Exoskeleton and Vision-Based Game Interface. *Journal of Intelligent Robotic Systems* **2019**, *96*, 31–47. https://doi.org/10.1007/s10846-018-0966-6.
267. Cisnal, A.; Pérez-Turiel, J.; Fraile, J.; Sierra, D.; De La Fuente, E. RobHand: A Hand Exoskeleton With Real-Time EMG-Driven Embedded Control. Quantifying Hand Gesture Recognition Delays for Bilateral Rehabilitation. *IEEE Access* **2021**, *9*, 137809–137823. https://doi.org/10.1109/access.2021.3118281.
268. Castiblanco, J.; Mondragón, I.; Alvarado-Rojas, C.; Colorado, J. Assist-As-Needed Exoskeleton for Hand Joint Rehabilitation Based on Muscle Effort Detection. *Sensors (Basel, Switzerland)* **2021**, *21*. https://doi.org/10.3390/s21134372.
269. Hsu, H.; Yang, K.C.; Yeh, C.; Lin, Y.C.; Lin, K.R.; Su, F.; Kuo, L. A Tenodesis-Induced-Grip exoskeleton robot (TIGER) for assisting upper extremity functions in stroke patients: a randomized control study. *Disability and Rehabilitation* **2021**, *44*, 7078–7086. https://doi.org/10.1080/09638288.2021.1980915.
270. Calabró, R.; Morone, G.; Naro, A.; Gandolfi, M.; Liotti, V.; D'aurizio, C.; Straudi, S.; Focacci, A.; Pournajaf, S.; Aprile, I.; et al. Robot-Assisted Training for Upper Limb in Stroke (ROBOTAS): An Observational, Multicenter Study to Identify Determinants of Efficacy. *Journal of Clinical Medicine* **2021**, *10*. https://doi.org/10.3390/jcm10225245.
271. Li, S.; Wang, Z.; Pang, Z.; Duan, Z.; Gao, M. Design and analysis of an upper limb exoskeleton robot for stroke rehabilitation. *2022 IEEE International Conference on Real-time Computing and Robotics (RCAR)* **2022**, pp. 573–578. https://doi.org/10.1109/rcar54675.2022.9872238.
272. Saita, K.; Morishita, T.; Hyakutake, K.; Ogata, T.; Fukuda, H.; Kamada, S.; Inoue, T. Feasibility of Robot-assisted Rehabilitation in Poststroke Recovery of Upper Limb Function Depending on the Severity. *Neurologia medico-chirurgica* **2020**, *60*, 217–222. https://doi.org/10.2176/nmc.oa.2019-0268.
273. Dudley, D.; Knarr, B.; Siu, K.; Peck, J.; Ricks, B.; Zuniga, J. Testing of a 3D printed hand exoskeleton for an individual with stroke: a case study. *Disability and Rehabilitation: Assistive Technology* **2019**, *16*, 209–213. https://doi.org/10.1080/17483107.2019.1646823.
274. Kim, H.; Miller, L.; Fedulow, I.; Simkins, M.; Abrams, G.; Byl, N.; Rosen, J. Kinematic Data Analysis for Post-Stroke Patients Following Bilateral Versus Unilateral Rehabilitation With an Upper Limb Wearable Robotic System. *IEEE Transactions on Neural Systems and Rehabilitation Engineering* **2013**, *21*, 153–164. https://doi.org/10.1109/tnsre.2012.2207462.
275. Keeling, A.; Piitz, M.; Semrau, J.; Hill, M.; Scott, S.; Dukelow, S. Robot enhanced stroke therapy optimizes rehabilitation (RESTORE): a pilot study. *Journal of NeuroEngineering and Rehabilitation* **2021**, *18*. https://doi.org/10.1186/s12984-021-00804-8.
276. Islam, R.; Spiewak, C.; Rahman, M.; Fareh, R. A Brief Review on Robotic Exoskeletons for Upper Extremity Rehabilitation to Find the Gap between Research Porotype and Commercial Type **2017**. *6*, 1–12. https://doi.org/10.4172/2168-9695.1000177.
277. Chen, W.; Li, G.; Li, N.; Wang, W.; Peng, Y.; Wang, R.; Xue, X.; Zhao, X.; Liu, L. Restoring Voluntary Bimanual Activities of Patients With Chronic Hemiparesis Through a Foot-Controlled Hand/Forearm Exoskeleton. *IEEE Transactions on Neural Systems and Rehabilitation Engineering* **2023**, *31*, 769–778. https://doi.org/10.1109/tnsre.2022.3233631.
278. Stewart, A.; Pretty, C.; Adams, M.; Chen, X. Review of Upper Limb Hybrid Exoskeletons. *IFAC-PapersOnLine* **2017**, *50*, 15169–15178. https://doi.org/10.1016/j.ifacol.2017.08.2266.
279. Tao, J.; Yu, S. Developing Conceptual PSS Models of Upper Limb Exoskeleton based Post-stroke Rehabilitation in China. *Procedia CIRP* **2019**. https://doi.org/10.1016/j.procir.2019.01.031.
280. Lejeune, T.; Nuic, D.; Dehem, S.; Previnaire, J.; Cuenot, C.; Debugne, T.; Kaps, J.; Paul, B.; Pean, V.; Perez, S.S.; et al. Hands-free Atalante exoskeleton in post-stroke gait and balance rehabilitation: a safety study. *Journal of NeuroEngineering and Rehabilitation* **2025**, *22*. https://doi.org/10.1186/s12984-025-01621-z.
281. Di Luzio, S.; Cordella, F.; Bravi, M.; Santacaterina, F.; Bressi, F.; Sterzi, S.; Zollo, L. Modification of Hand Muscular Synergies in Stroke Patients after Robot-Aided Rehabilitation. *Applied Sciences* **2022**. https://doi.org/10.3390/app12063146.
282. Frisoli, A. Exoskeletons for upper limb rehabilitation **2018**. pp. 75–87. https://doi.org/10.1016/b978-0-12-811995-2.00006-0.
283. Esposito, D.; Centracchio, J.; Andreozzi, E.; Savino, S.; Gargiulo, G.; Naik, G.; Bifulco, P. Design of a 3D-Printed Hand Exoskeleton Based on Force-Myography Control for Assistance and Rehabilitation. *Machines* **2022**. https://doi.org/10.3390/machines10010057.
284. Syringas, P.; Economopoulos, T.; Kouris, I.; Kakkos, I.; Papagiannis, G.; Triantafyllou, A.; Tselikas, N.; Matsopoulos, G.; Fotiadis, D. Rehabotics: A Comprehensive Rehabilitation Platform for Post-Stroke Spasticity, Incorporating a Soft Glove, a Robotic Exoskeleton Hand and Augmented Reality Serious Games. *ABSET 2023* **2023**. https://doi.org/10.3390/engproc2023050002.

285. Schabowsky, C.; Godfrey, S.; Holley, R.; Lum, P. Development and pilot testing of HEXORR: Hand EXOskeleton Rehabilitation Robot. *Journal of NeuroEngineering and Rehabilitation* **2010**, *7*, 36–36. https://doi.org/10.1186/1743-0003-7-36.
286. Bauer, G.; Pan, Y. Review of Control Methods for Upper Limb Telerehabilitation With Robotic Exoskeletons. *IEEE Access* **2020**, *8*, 203382–203397. https://doi.org/10.1109/access.2020.3036596.
287. Aggogeri, F.; Mikołajczyk, T.; O'Kane, J. Robotics for rehabilitation of hand movement in stroke survivors. *Advances in Mechanical Engineering* **2019**, *11*. https://doi.org/10.1177/1687814019841921.
288. Ren, Y.; Kang, S.; Park, H.; Wu, Y.N.; Zhang, L.Q. Developing a Multi-Joint Upper Limb Exoskeleton Robot for Diagnosis, Therapy, and Outcome Evaluation in Neurorehabilitation. *IEEE Transactions on Neural Systems and Rehabilitation Engineering* **2013**, *21*, 490–499. https://doi.org/10.1109/tnsre.2012.2225073.
289. Amici, C.; Borboni, A.; Faglia, R.; Fausti, D.; Magnani, P.L. A parallel compliant meso-manipulator for finger rehabilitation treatments: Kinematic and dynamic analysis. 2008, pp. 735 – 740. Cited by: 27, https://doi.org/10.1109/IROS.2008.4651029.
290. Amici, C.; Borboni, A.; Magnani, P.L.; Pomi, D. Kinematic analysis of a compliant, parallel and three-dimensional meso-manipulator generated from a planar structure **2009**. pp. 479 – 485. Cited by: 16, https://doi.org/10.1007/978-1-4020-8915-2_58.
291. Amici, C.; Borboni, A.; Faglia, R. A compliant PKM mesomanipulator: Kinematic and dynamic analyses. *Advances in Mechanical Engineering* **2010**, *2010*. Cited by: 27; All Open Access; Gold Open Access; Green Final Open Access; Green Open Access, https://doi.org/10.1155/2010/706023.
292. Tiboni, M.; Borboni, A.; Mor, M.; Pomi, D. An innovative pneumatic mini-valve actuated by SMA Ni-Ti wires: Design and analysis. *Proceedings of the Institution of Mechanical Engineers. Part I: Journal of Systems and Control Engineering* **2011**, *225*, 443 – 451. Cited by: 30, https://doi.org/10.1177/2041304110394531.
293. Villafañe, J.H.; Silva, G.B.; Fernández-Carnero, J. Effect of thumb joint mobilization on pressure pain threshold in elderly patients with thumb carpometacarpal osteoarthritis. *Journal of Manipulative and Physiological Therapeutics* **2012**, *35*, 110 – 120. Cited by: 49, https://doi.org/10.1016/j.jmpt.2011.12.002.
294. Aggogeri, F.; Borboni, A.; Faglia, R.; Merlo, A.; de Cristofaro, S. Precision Positioning Systems: An overview of the state of art. *Applied Mechanics and Materials* **2013**, *336-338*, 1170 – 1173. Cited by: 22, https://doi.org/10.4028/www.scientific.net/AMM.336-338.1170.
295. Aggogeri, F.; Borboni, A.; Faglia, R. Reliability roadmap for mechatronic systems. *Applied Mechanics and Materials* **2013**, *373-375*, 130 – 133. Cited by: 26, https://doi.org/10.4028/www.scientific.net/AMM.373-375.130.
296. Borboni, A.; Faglia, R. Stochastic evaluation and analysis of free vibrations in simply supported piezoelectric bimorphs. *Journal of Applied Mechanics* **2013**, *80*. Cited by: 27, https://doi.org/10.1115/1.4007721.
297. Villafañe, J.H.; Pillastrini, P.; Borboni, A. Manual therapy and neurodynamic mobilization in a patient with peroneal nerve paralysis: A case report. *Journal of Chiropractic Medicine* **2013**, *12*, 176 – 181. Cited by: 14; All Open Access; Green Accepted Open Access; Green Open Access, https://doi.org/10.1016/j.jcm.2013.10.007.
298. Villafañe, J.H.; Cleland, J.A.; Fernández De Las Peñas, C. Bilateral sensory effects of unilateral passive accessory mobilization in patients with thumb carpometacarpal osteoarthritis. *Journal of Manipulative and Physiological Therapeutics* **2013**, *36*, 232 – 237. Cited by: 46, https://doi.org/10.1016/j.jmpt.2013.05.008.
299. Villafañe, J.H.; Bishop, M.D.; Fernández De Las Peñas, C.; Langford, D.P. Radial nerve mobilisation had bilateral sensory effects in people with thumb carpometacarpal osteoarthritis: A randomised trial. *Journal of Physiotherapy* **2013**, *59*, 25 – 30. Cited by: 47; All Open Access; Gold Open Access; Green Accepted Open Access; Green Open Access, https://doi.org/10.1016/S1836-9553(13)70143-7.
300. Borboni, A.; Faglia, R.; Mor, M. Compliant device for hand rehabilitation of stroke patient. 2014, Vol. 1. Cited by: 8, https://doi.org/10.1115/ESDA2014-20081.
7Borboni, A.; Faglia, R. Characterization of a new SMA actuator. 2014, Vol. 87, pp. 1378 – 1381. Cited by: 12; All Open Access; Gold Open Access; Green Accepted Open Access; Green Open Access, https://doi.org/10.1016/j.proeng.2014.11.699.
301. Pandini, S.; Riccò, T.; Borboni, A.; Bodini, I.; Vetturi, D.; Cambiaghi, D.; Toselli, M.; Paderni, K.; Messori, M.; Pilati, F.; et al. Tailored one-way and two-way shape memory capabilities of poly(-Caprolactone)-based systems for biomedical applications. *Journal of Materials Engineering and Performance* **2014**, *23*, 2545 – 2552. Cited by: 18; All Open Access; Green Accepted Open Access; Green Open Access, https://doi.org/10.1007/s11665-014-1033-5.
303. Pandini, S.; Borboni, A.; Bodini, I.; Vetturi, D.; Cambiaghi, D.; Paderni, K.; Messori, M.; Toselli, M.; Riccò, T. Evaluation of the shape memory performances of poly(-caprolactone)- based tubular devices for potential biomedical applications. 2014, Vol. 1599, pp. 306 – 309. Cited by: 2; All Open Access; Green Accepted Open Access; Green Open Access, https://doi.org/10.1063/1.4876839.
304. Taveggia, G.; Villafañe, J.H.; Vavassori, F.; Lecchi, C.; Borboni, A.; Negrini, S. Multimodal treatment of distal sensorimotor polyneuropathy in diabetic patients: A randomized clinical trial. *Journal of Manipulative and Physiological Therapeutics* **2014**, *37*, 242 – 252. Cited by: 48, https://doi.org/10.1016/j.jmpt.2013.09.007.
305. Amici, C.; Borboni, A.; Tuveggia, G.; Legnani, G. Bioelectric prostheses: Review of classifications and control strategies. *Giornale Italiano di Medicina del Lavoro ed Ergonomia* **2015**, *37*, 39 – 44. Cited by: 3.

306. Borboni, A.; Lancini, M. Commanded motion optimization to reduce residual vibration. *Journal of Vibration and Acoustics* **2015**, *137*. Cited by: 28, https://doi.org/10.1115/1.4029575.
307. Fogliaresi, S.; Mulé, C.; Borboni, A.; Taveggia, G. The path to customized prosthesis: Role of the occupational therapist in the recovery of autonomy; Il percorso protesico personalizzato: Ruolo del T.O. Nel recupero dell'autonomia. *Giornale Italiano di Medicina del Lavoro ed Ergonomia* **2015**, *37*, 34 – 38. Cited by: 0.
308. Taveggia, G.; Borboni, A. Epidemiology of amputations: From medical causes to traumatic ones and at work; Epidemiologia delle amputazioni: Dalle cause mediche a quelle traumatiche e sul lavoro. *Giornale Italiano di Medicina del Lavoro ed Ergonomia* **2015**, *37*, 13 – 15. Cited by: 0.
309. Taveggia, G.; Ragusa, I.; Trani, V.; Cuva, D.; Angeretti, C.; Fontanella, M.M.; Panciani, P.P.; Borboni, A. Robotic tilt table reduces the occurrence of orthostatic hypotension over time in vegetative states. *International Journal of Rehabilitation Research* **2015**, *38*, 162 – 166. Cited by: 16, https://doi.org/10.1097/MRR.0000000000000104.
310. Villafañe, J.H.; Valdes, K.A.; Vanti, C.; Pillastrini, P.; Borboni, A. Reliability of handgrip strength test in elderly subjects with unilateral thumb carpometacarpal osteoarthritis. *Hand* **2015**, *10*, 205 – 209. Cited by: 33; All Open Access; Green Accepted Open Access; Green Open Access, https://doi.org/10.1007/s11552-014-9678-y.
311. Aggogeri, F.; Borboni, A.; Merlo, A.; Pellegrini, N.; Ricatto, R. Real-time performance of mechatronic PZT module using active vibration feedback control. *Sensors* **2016**, *16*. Cited by: 29; All Open Access; Gold Open Access; Green Accepted Open Access; Green Open Access, https://doi.org/10.3390/s16101577.
312. Aggogeri, F.; Borboni, A.; Pellegrini, N. Jerk trajectory planning for assistive and rehabilitative mechatronic devices. *International Review of Mechanical Engineering* **2016**, *10*, 543 – 551. Cited by: 11, https://doi.org/10.15866/ireme.v10i7.10015.
313. Borboni, A.; Mor, M.; Faglia, R. Gloreha-Hand Robotic Rehabilitation: Design, Mechanical Model, and Experiments. *Journal of Dynamic Systems, Measurement and Control* **2016**, *138*. Cited by: 122, https://doi.org/10.1115/1.4033831.
314. Rastegarpanah, A.; Sadaat, M.H.; Borboni, A. Parallel Robot for Lower Limb Rehabilitation Exercises. *Applied Bionics and Biomechanics* **2016**, *2016*. Cited by: 35; All Open Access; Gold Open Access; Green Accepted Open Access; Green Open Access, https://doi.org/10.1155/2016/8584735.
315. Taveggia, G.; Borboni, A.; Mulé, C.; Villafañe, J.H.; Negrini, S. Conflicting results of robot-assisted versus usual gait training during postacute rehabilitation of stroke patients: A randomized clinical trial. *International Journal of Rehabilitation Research* **2016**, *39*, 29 – 35. Cited by: 85; All Open Access; Green Accepted Open Access; Green Open Access; Hybrid Gold Open Access, https://doi.org/10.1097/MRR.0000000000000137.
316. Taveggia, G.; Borboni, A.; Salvi, L.; Mulé, C.; Fogliaresi, S.; Villafañe, J.H.; Casale, R. Efficacy of robot-assisted rehabilitation for the functional recovery of the upper limb in post-stroke patients: A randomized controlled study. *European Journal of Physical and Rehabilitation Medicine* **2016**, *52*, 767 – 773. Cited by: 63.
317. Villafañe, J.H.; Valdes, K.A.; Buraschi, R.; Martinelli, M.; Bissolotti, L.M.; Negrini, S. Reliability of the handgrip strength test in elderly subjects with parkinson disease. *Hand* **2016**, *11*, 54 – 58. Cited by: 47, https://doi.org/10.1177/1558944715614852.
318. Abagnale, C.; Aggogeri, F.; Borboni, A.; Strano, S.; Terzo, M. Dead-zone effect on the performance of state estimators for hydraulic actuators. *Meccanica* **2017**, *52*, 2189 – 2199. Cited by: 7, https://doi.org/10.1007/s11012-016-0563-3.
319. Borboni, A.; Villafañe, J.H.; Mullè, C.; Valdes, K.A.; Faglia, R.; Taveggia, G.; Negrini, S. Robot-Assisted Rehabilitation of Hand Paralysis After Stroke Reduces Wrist Edema and Pain: A Prospective Clinical Trial. *Journal of Manipulative and Physiological Therapeutics* **2017**, *40*, 21 – 30. Cited by: 38; All Open Access; Bronze Open Access; Green Accepted Open Access; Green Open Access, https://doi.org/10.1016/j.jmpt.2016.10.003.
320. Negrini, S.; Serpelloni, M.; Amici, C.; Gobbo, M.; Silvestro, C.; Buraschi, R.; Borboni, A.; Crovato, D.; Lopomo, N.F. Use of wearable inertial sensor in the assessment of Timed-Up-and-Go Test: Influence of device placement on temporal variable estimation. *Lecture Notes of the Institute for Computer Sciences, Social-Informatics and Telecommunications Engineering, LNICST* **2017**, *192*, 310 – 317. Cited by: 29, https://doi.org/10.1007/978-3-319-58877-3_40.
321. Rastegarpanah, A.; Sadaat, M.H.; Borboni, A.; Stolkin, R. Application of a parallel robot in lower limb rehabilitation: A brief capability study. 2017. Cited by: 11, https://doi.org/10.1109/RAHA.2016.7931865.
322. Tageldeen, M.K.; Elamvazhuthi, I.; Nallagownden, P.A.; Ganesan, T.; Borboni, A. Development of a 3D virtual platform for an upper arm robotic exoskeleton. 2017. Cited by: 4, https://doi.org/10.1109/ROMA.2016.7847819.
323. Villafañe, J.H.; Valdes, K.A.; Imperio, G.; Borboni, A.; Cantero Téllez, R.; Galeri, S.; Negrini, S. Neural manual vs. robotic assisted mobilization to improve motion and reduce pain hypersensitivity in hand osteoarthritis: Study protocol for a randomized controlled trial. *Journal of Physical Therapy Science* **2017**, *29*, 801 – 806. Cited by: 8; All Open Access; Gold Open Access; Green Accepted Open Access; Green Open Access, https://doi.org/10.1589/jpts.29.801.
324. Al-Quraishi, M.S.; Elamvazhuthi, I.; Daud, S.A.; Parasuraman, S.; Borboni, A. Eeg-based control for upper and lower limb exoskeletons and prostheses: A systematic review. *Sensors* **2018**, *18*. Cited by: 130; All Open Access; Gold Open Access; Green Accepted Open Access; Green Open Access, https://doi.org/10.3390/s18103342.

325. Borboni, A.; Faglia, R. Robust design of a shape memory actuator with slider and slot layout and passive cooling control. *Microsystem Technologies* **2018**, *24*, 1379 – 1389. Cited by: 7, https://doi.org/10.1007/s00542-016-2998-9.
326. Borboni, A.; Serpelloni, M.; Borghetti, M.; Amici, C.; Aggogeri, F.; Fausti, D.; Antonini, M.; Mor, M.; Sardini, E.; Faglia, R. Hand robotic rehabilitation: From hospital to home. *Mechanisms and Machine Science* **2018**, *49*, 877 – 884. Cited by: 3, https://doi.org/10.1007/978-3-319-61276-8_93.
327. Mikolajczyk, T.; Kłodowski, A.; Mikołajewska, E.; Walkowiak, P.; Berjano, P.L.; Villafañe, J.H.; Aggogeri, F.; Borboni, A.; Fausti, D.; Petrogalli, G. Design and control of system for elbow rehabilitation: Preliminary findings. *Advances in Clinical and Experimental Medicine* **2018**, *27*, 1661 – 1669. Cited by: 21; All Open Access; Gold Open Access; Green Accepted Open Access; Green Open Access, https://doi.org/10.17219/acem/74556.
328. Rastegarpanah, A.; Rakhodaei, H.; Sadaat, M.H.; Rastegarpanah, M.; Marturi, N.; Borboni, A.; Loureiro, R.C. Path-planning of a hybrid parallel robot using stiffness and workspace for foot rehabilitation. *Advances in Mechanical Engineering* **2018**, *10*. Cited by: 16; All Open Access; Gold Open Access; Green Accepted Open Access; Green Open Access, https://doi.org/10.1177/1687814017754159.
329. Sadaat, M.H.; Rastegarpanah, A.; Abdullah, C.Z.; Rakhodaei, H.; Borboni, A.; Maddalena, M. Path's slicing analysis as a therapist's intervention tool for robotic rehabilitation. *Mechanisms and Machine Science* **2018**, *49*, 901 – 910. Cited by: 1, https://doi.org/10.1007/978-3-319-61276-8_96.
330. Tiboni, M.; Borboni, A.; Faglia, R.; Pellegrini, N. Robotics rehabilitation of the elbow based on surface electromyography signals. *Advances in Mechanical Engineering* **2018**, *10*. Cited by: 36; All Open Access; Gold Open Access; Green Accepted Open Access; Green Open Access, https://doi.org/10.1177/1687814018754590.
331. Villafañe, J.H.; Taveggia, G.; Galeri, S.; Bissolotti, L.M.; Mullè, C.; Imperio, G.; Valdes, K.A.; Borboni, A.; Negrini, S. Efficacy of Short-Term Robot-Assisted Rehabilitation in Patients With Hand Paralysis After Stroke: A Randomized Clinical Trial. *Hand* **2018**, *13*, 95 – 102. Cited by: 70; All Open Access; Green Accepted Open Access; Green Open Access, https://doi.org/10.1177/1558944717692096.
332. Aggogeri, F.; Borboni, A.; Pellegrini, N.; Adamini, R. Design and development of a mechanism for lower limb movement. *International Journal of Mechanical Engineering and Robotics Research* **2019**, *8*, 911 – 920. Cited by: 4; All Open Access; Green Accepted Open Access; Green Open Access, https://doi.org/10.18178/ijmerr.8.6.911-920.
333. Ceresoli, F.; Aggogeri, F.; Amici, C.; Borboni, A.; Faglia, R.; Pellegrini, N.; Tiboni, M.; Antonini, M.; Fausti, D.; Mor, M.; et al. Differential system for limb rehabilitation. *Mechanisms and Machine Science* **2019**, *65*, 3 – 10. Cited by: 9, https://doi.org/10.1007/978-3-030-00329-6_1.
334. Borboni, A.; Giuseppe, C.; Pellegrini, N. Reference Frame Identification and Distributed Control Strategies in Human-Robot Collaboration. *Mechanisms and Machine Science* **2020**, *84*, 93 – 102. Cited by: 2, https://doi.org/10.1007/978-3-030-48989-2_11.
335. Ragni, F.; Amici, C.; Borboni, A.; Faglia, R.; Cappellini, V.; Pedersini, P.; Villafañe, J.H. Effects of soft tissue artifact in the measurement of hand kinematics. *International Review of Mechanical Engineering* **2020**, *14*, 230 – 242. Cited by: 9, https://doi.org/10.15866/ireme.v14i4.18253.
336. Al-Quraishi, M.S.; Elamvazhuthi, I.; Tang, T.B.; Al-Qurishi, M.S.; Parasuraman, S.; Borboni, A. Detection of Lower Limb Movements using Sensorimotor Rhythms. 2021. Cited by: 4, https://doi.org/10.1109/ICIAS49414.2021.9642696.
337. Al-Quraishi, M.S.; Elamvazhuthi, I.; Tang, T.B.; Al-Qurishi, M.S.; Parasuraman, S.; Borboni, A. Multimodal Fusion Approach Based on EEG and EMG Signals for Lower Limb Movement Recognition. *IEEE Sensors Journal* **2021**, *21*, 27640 – 27650. Cited by: 47, https://doi.org/10.1109/JSEN.2021.3119074.
338. Al-Quraishi, M.S.; Elamvazhuthi, I.; Tang, T.B.; Al-Qurishi, M.S.; Parasuraman, S.; Borboni, A. Lower limb Movements' Classifications using Hemodynamic Response:fNIRS Study. 2021, pp. 76 – 81. Cited by: 4, https://doi.org/10.1109/IECBES48179.2021.9398783.
339. Pagani, R.; Nuzzi, C.; Ghidelli, M.; Borboni, A.; Lancini, M.; Legnani, G. Cobot user frame calibration: Evaluation and comparison between positioning repeatability performances achieved by traditional and vision-based methods. *Robotics* **2021**, *10*. Cited by: 22; All Open Access; Gold Open Access; Green Accepted Open Access; Green Open Access, https://doi.org/10.3390/robotics10010045.
340. Sconza, C.; Negrini, F.; Di Matteo, B.; Borboni, A.; Boccia, G.; Petrikonis, I.; Stankevicˇius, E.; Casale, R. Robot-assisted gait training in patients with multiple sclerosis: A randomized controlled crossover trial. *Medicina (Lithuania)* **2021**, *57*. Cited by: 30; All Open Access; Gold Open Access; Green Accepted Open Access; Green Open Access, https://doi.org/10.3390/medicina57070713.
341. Al-Quraishi, M.S.; Elamvazhuthi, I.; Tang, T.B.; Al-Qurishi, M.S.; Adil, S.H.; Ebrahim, M.; Borboni, A. Decoding the User's Movements Preparation From EEG Signals Using Vision Transformer Architecture. *IEEE Access* **2022**, *10*, 109446 – 109459. Cited by: 14; All Open Access; Gold Open Access; Green Accepted Open Access; Green Open Access, https://doi.org/10.1109/ACCESS.2022.3213996.
342. Amici, C.; Mosso, M.; Verité, F.; Tiboni, M.; Borboni, A.; Negrini, S. Trunk Movement Analysis with FDA: Exploratory Study for the Healthy Subject. 2022. Cited by: 0, https://doi.org/10.1109/MESA55290.2022.10004476.

343. Amici, C.; Cappellini, V.; Ragni, F.; Formicola, R.; Borboni, A.; Piovanelli, B.; Negrini, S.; Candiani, G. Trunk Flexion-Extension in Healthy Subjects: Preliminary Analysis of Movement Profiles. *Mechanisms and Machine Science* **2022**, *106 MMS*, 155 – 163. Cited by: 0, https://doi.org/10.1007/978-3-030-76147-9_17.
344. Borboni, A.; Elamvazhuthi, I.; Cusano, N. EEG-Based Empathic Safe Cobot. *Machines* **2022**, *10*. Cited by: 9; All Open Access; Gold Open Access; Green Final Open Access; Green Open Access, https://doi.org/10.3390/machines10080603.
345. Formicola, R.; Ragni, F.; Borboni, A.; Amici, C. Design Process of Medical Devices for Robotic Rehabilitation: An Open Innovation-Inspired Approach. *Smart Innovation, Systems and Technologies* **2022**, *247*, 575 – 584. Cited by: 7, https://doi.org/10.1007/978-981-16-3844-2_51.
346. Tiboni, M.; Borboni, A.; Verité, F.; Bregoli, C.; Amici, C. Sensors and Actuation Technologies in Exoskeletons: A Review. *Sensors* **2022**, *22*. Cited by: 139; All Open Access; Gold Open Access; Green Accepted Open Access; Green Open Access, https://doi.org/10.3390/s22030884.
347. Formicola, R.; Amici, C.; Mor, M.; Bissolotti, L.M.; Borboni, A. Design of Medical Devices with Usability in Mind: A Theoretical Proposal and Experimental Case Study Using the LEPRE Device. *Designs* **2023**, *7*. Cited by: 9; All Open Access; Gold Open Access; Green Accepted Open Access; Green Open Access, https://doi.org/10.3390/designs7010009.
348. Borboni, A.; Arbore, A.; Elamvazhuthi, I. EWA: A Single Size Self-adapting Upper Limb Exoskeleton Without Adjustment. *Mechanisms and Machine Science* **2024**, *159 MMS*, 37 – 51. Cited by: 0, https://doi.org/10.1007/978-3-031-60618-2_4.
349. Borboni, A.; Arbore, A.; Elamvazhuthi, I. EWA 2: A Single Size Self-adapting Exoskeleton Without Adjustment for the Upper Limb. *Mechanisms and Machine Science* **2024**, *164 MMS*, 555 – 562. Cited by: 0, https://doi.org/10.1007/978-3-031-64569-3_63.
350. Zanoletti, F.; Borboni, A.; Pignolo, L.; Vatrano, M. Integrating Clinical Expertise in the Design of Home-Based Smart Rehabilitation Devices for Motor and Cognitive Therapy in Patients with Neurological Disorders. *Mechanisms and Machine Science* **2025**, *179*, 257 – 264. Cited by: 0, https://doi.org/10.1007/978-3-031-91151-4_29.
351. Zanoletti, F.; Borboni, A. Descriptive Ontology for Home-Based Smart Rehabilitation Environments for Motor and Cognitive Therapy in Patients with Neurological Disorders. *Mechanisms and Machine Science* **2025**, *190*, 260 – 267. Cited by: 0, https://doi.org/10.1007/978-3-032-02106-9_29.
352. Bertozzi, L.; Valdes, K.A.; Vanti, C.; Negrini, S.; Pillastrini, P.; Villafañe, J.H. Investigation of the effect of conservative interventions in thumb carpometacarpal osteoarthritis: Systematic review and meta-analysis. *Disability and Rehabilitation* **2015**, *37*, 2025 – 2043. Cited by: 109, https://doi.org/10.3109/09638288.2014.996299.
353. Bruno, M.C.; Amadei, F.; Basile, G.; Sabbatini, M.; Renò, F.; Colò, G.; Surace, M.F.; Villafañe, J.H.; Leigheb, M. Brachial plexus schwannomas. *Chirurgia (Turin)* **2025**, *38*, 326 – 330. Cited by: 0, https://doi.org/10.23736/S0394-9508.25.05907-8.
354. Cantero Téllez, R.; Valdes, K.A.; O'Brien, V.; Berjano, P.L.; Pedersini, P.; Villafañe, J.H. Building Legitimacy by Valuing the Expert Opinion in the Conservative Treatment of Thumb Carpometacarpal Joint Osteoarthritis. *Topics in Geriatric Rehabilitation* **2019**, *35*, 156 – 159. Cited by: 2, https://doi.org/10.1097/TGR.0000000000000226.
355. Martínez-Pozas, O.; Sánchez-Romero, E.A.; Beltran-Alacreu, H.; Arribas-Romano, A.; Cuenca-Martínez, F.; Villafañe, J.H.; Fernández-Carnero, J. Effects of Orthopedic Manual Therapy on Pain Sensitization in Patients with Chronic Musculoskeletal Pain: An Umbrella Review with Meta-Meta-analysis. *American Journal of Physical Medicine and Rehabilitation* **2023**, *102*, 879 – 885. Cited by: 46, https://doi.org/10.1097/PHM.0000000000002239.
356. Mingo, D.B.D.; Ramírez, G.P.; Garcia, A.M.; Alberdi, M.T.; Muñoz, D.N.; Abad, M.P.; Rossettini, G.; Villafañe, J.H.; Miralles, C.A.; Jou, M.D.V.; et al. Annular Ligament Instability in Lateral Elbow Pathology: Objective Confirmation Through a Cadaveric Study. *Muscles* **2025**, *4*. Cited by: 0; All Open Access; Gold Open Access; Green Accepted Open Access; Green Open Access, https://doi.org/10.3390/muscles4030039.
357. Pedersini, P.; Villafañe, J.H.; Piovanelli, B.; Cappellini, V.; Buraschi, R.; Pollet, J.; Negrini, S. P 106 - Intra- and inter-operator reliability of a novel hand protocol during grip movement in healthy subjects. *Gait and Posture* **2018**, *65*, 409 – 410. Cited by: 1, https://doi.org/10.1016/j.gaitpost.2018.07.029.
358. Pedersini, P.; López-Royo, M.P.; Herrero, P.; Cantero Téllez, R.; Valdes, K.A.; Touche, R.L.; Fernández-Carnero, J.; Villafañe, J.H. Prevalence of Myofascial Trigger Points in the First Dorsal Interosseous Muscle in Patients With Thumb Carpometacarpal Osteoarthritis Compared to Healthy Controls. *Topics in Geriatric Rehabilitation* **2021**, *37*, 214 – 221. Cited by: 6, https://doi.org/10.1097/TGR.0000000000000331.
359. Ribeiro, I.L.; Camargo, P.R.; Alburquerque-Sendín, F.; Madeleine, P.; Fernández De Las Peñas, C.; Salvini, T.F. Topographical pressure pain sensitivity maps of the shoulder region in individuals with subacromial pain syndrome. *Manual Therapy* **2016**, *21*, 134 – 143. Cited by: 22, https://doi.org/10.1016/j.math.2015.07.002.
360. Villafañe, J.H.; Silva, G.B.; Fernández-Carnero, J. Short-term effects of neurodynamic mobilization in 15 patients with secondary thumb carpometacarpal osteoarthritis. *Journal of Manipulative and Physiological Therapeutics* **2011**, *34*, 449 – 456. Cited by: 50, https://doi.org/10.1016/j.jmpt.2011.05.016.

361. Villafañe, J.H.; Silva, G.B.; Angulo-Díaz-Parreño, S.; Fernández-Carnero, J. Hypoalgesic and motor effects of Kaltenborn mobilization on elderly patients with secondary thumb carpometacarpal osteoarthritis: A randomized controlled trial. *Journal of Manipulative and Physiological Therapeutics* **2011**, *34*, 547 – 556. Cited by: 57, https://doi.org/10.1016/j.jmpt.2011.08.005.
362. Villafañe, J.H.; Silva, G.B.; Bishop, M.D.; Fernández-Carnero, J. Radial nerve mobilization decreases pain sensitivity and improves motor performance in patients with thumb carpometacarpal osteoarthritis: A randomized controlled trial. *Archives of Physical Medicine and Rehabilitation* **2012**, *93*, 396 – 403. Cited by: 65, https://doi.org/10.1016/j.apmr.2011.08.045.
363. Villafañe, J.H.; Valdes, K.A. Reliability of pinch strength testing in elderly subjects with unilateral thumb carpometacarpal osteoarthritis. *Journal of Physical Therapy Science* **2014**, *26*, 993 – 995. Cited by: 50; All Open Access; Gold Open Access; Green Accepted Open Access; Green Open Access, https://doi.org/10.1589/jpts.26.993.
364. Villafañe, J.H.; Valdes, K.A.; Bertozzi, L.; Negrini, S. Minimal clinically important difference of grip and pinch strength in women with thumb carpometacarpal osteoarthritis when compared to healthy subjects. *Rehabilitation Nursing* **2017**, *42*, 139 – 145. Cited by: 61, https://doi.org/10.1002/rnj.196.
365. Zhang, T.; Zheng, K.; Tao, H.; Liu, J. A Soft Wearable Modular Assistive Glove Based on Novel Miniature Foldable Pouch Motor Unit. *Advanced Intelligent Systems* **2025**, *7*. Cited by: 4; All Open Access, Gold Open Access, Green Open Access, https://doi.org/10.1002/aisy.202500274.
366. Tong, M.; Cheung, M.; Lei, Y.; Villarroel, M.; He, L. Vision-Based Hand Function Evaluation with Soft Robotic Rehabilitation Glove. *Sensors* **2026**, *26*. Cited by: 0; All Open Access, Gold Open Access, Green Open Access, https://doi.org/10.3390/s26010138.
367. Lu, J.; Lv, X.; Liu, J.; Liu, H.; Huang, T. Fully flexible wearable pouch pneumatic artificial muscle based on asymmetric expansion. *Chinese Journal of Mechanical Engineering (English Edition)* **2026**, *39*. Cited by: 11; All Open Access, Gold Open Access, https://doi.org/10.1016/j.cjme.2025.100085.
368. Bhat, S.K.; Doreswamy, D.; Hegde, A.H.; Nukarapu, V.K.; Bhat, S.; Puneeth, S.; Das, V.; Bayezeed, A.A.; Bongale, A.M. Numerical and experimental methods for the assessment of a human finger-inspired soft pneumatic actuator for gripping applications. *MethodsX* **2025**, *14*, 103111. https://doi.org/https://doi.org/10.1016/j.mex.2024.103111.
